# A flavin-inspired covalent organic framework for photocatalytic alcohol oxidation†

**DOI:** 10.1039/d1sc04143f

**Published:** 2021-11-15

**Authors:** Stefan Trenker, Lars Grunenberg, Tanmay Banerjee, Gökcen Savasci, Laura M. Poller, Katharina I. M. Muggli, Frederik Haase, Christian Ochsenfeld, Bettina V. Lotsch

**Affiliations:** Max Planck Institute for Solid State Research Heisenbergstr. 1 70569 Stuttgart Germany b.lotsch@fkf.mpg.de; Department of Chemistry, University of Munich (LMU) Butenandtstr. 5-13 81377 Munich Germany; Center for Nanoscience Schellingstr. 4 80799 Munich Germany; Department of Chemistry, Birla Institute of Technology and Science Pilani, Pilani Campus Rajasthan 333031 India; Karlsruhe Institute of Technology (KIT), IFG – Institute for Functional Interfaces Hermann-von-Helmholtz-Platz 1, 76344 Eggenstein-Leopoldshafen Germany; e-conversion Cluster of Excellence Lichtenbergstr. 4a, 85748 Garching Germany

## Abstract

Covalent organic frameworks (COFs) offer a number of key properties that predestine them to be used as heterogeneous photocatalysts, including intrinsic porosity, long-range order, and light absorption. Since COFs can be constructed from a practically unlimited library of organic building blocks, these properties can be precisely tuned by choosing suitable linkers. Herein, we report the construction and use of a novel COF (FEAx-COF) photocatalyst, inspired by natural flavin cofactors. We show that the functionality of the alloxazine chromophore incorporated into the COF backbone is retained and study the effects of this heterogenization approach by comparison with similar molecular photocatalysts. We find that the integration of alloxazine chromophores into the framework significantly extends the absorption spectrum into the visible range, allowing for photocatalytic oxidation of benzylic alcohols to aldehydes even with low-energy visible light. In addition, the activity of the heterogeneous COF photocatalyst is less dependent on the chosen solvent, making it more versatile compared to molecular alloxazines. Finally, the use of oxygen as the terminal oxidant renders FEAx-COF a promising and “green” heterogeneous photocatalyst.

## Abstract 2

In this manuscript, we report the development of a novel alloxazine COF inspired by naturally occurring flavin cofactors for photoredox catalysis.

## Graphical abstract

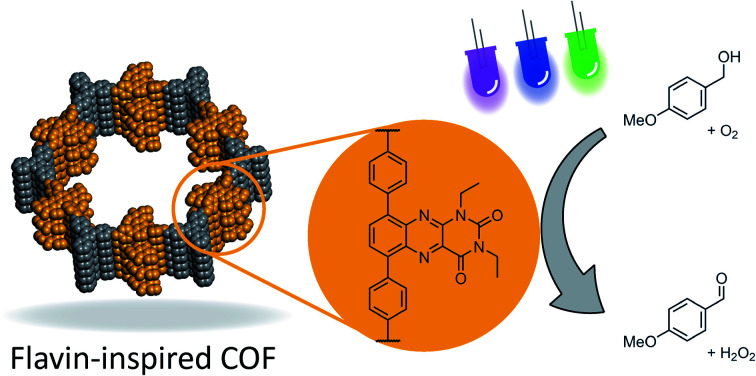

## Introduction

Metal-free photocatalysis is a promising strategy to address the ever-growing demand for green fuels and fine chemicals. Covalent organic frameworks (COFs), constructed from building blocks composed of earth abundant and light elements, are an emerging class of crystalline and porous polymers with significant potential in this regard. COFs have been explored as heterogeneous photocatalysts for solar hydrogen evolution,^1,2^ CO_2_ reduction,^3^ H_2_O_2_ generation,^4^ for example, and recent examples of C–H functionalization,^5–7^ sulfoxidation,^7–10^ and amine oxidation^7,11^ highlight their usefulness as photoredoxcatalysts. This catalytic versatility is mainly owed to the modular building principle underlying COF chemistry. Therefore, by choosing appropriate building blocks, structural and electronic characteristics of the final material such as pore size^12^ and optoelectronic properties^13^ – and thus ultimately its reactivity – can be tuned to the desired effect. Integration of suitable linker functionalities into the framework is therefore of prime importance in this regard, as recently exemplified by the induction of chirality^14^ or redox-activity^15^ to the COF backbone.

Photoredox catalysis is particularly useful in organic chemistry to overcome the activation energy of a particular reaction, to enable milder reaction conditions, or to grant access to orthogonal reaction products and pathways which are not accessible by classical methods. However, photoredox catalysis is often conducted using precious transition-metal complexes.^16–19^ In recent times though, a number of metal-free approaches using organic chromophores have been reported: fluorenone,^20^ acridinium ions,^21,22^ and eosin Y^23^ are just a few examples.

**Chart 1:**
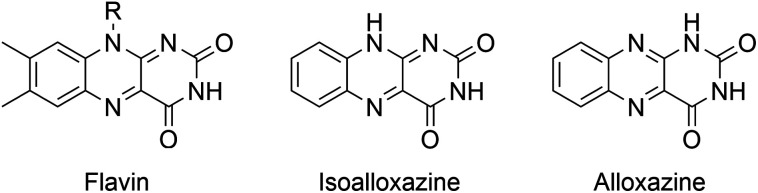
Molecular structure of flavin and (iso)alloxazine. For riboflavin R = ribityl.

Mostly owing to their ability to participate in both one- and two-electron redox reactions, flavins, derived from the vitamin riboflavin, represent a particularly interesting family of organic photocatalysts (Chart 1). Depending on the substitution pattern, flavin derivatives can be used for a plethora of catalytic reactions, such as esterifications,^24^ alkene hydrogenation,^25^ or oxidation of amines,^26–28^ sulfides,^26,27,29–32^ and alcohols.^32–40^

Alloxazines, isomers of the isoalloxazine heterocycle inherent to flavins,^41^ have attracted less attention in comparison. Nevertheless, alloxazines have been shown to be superior singlet oxygen sensitizers,^42^ and more efficient photocatalysts in [2 + 2] cycloaddition reactions.^43,44^ Also, alloxazines are easier to synthesize and more photostable than isoalloxazines.^45^ Despite their versatility, alloxazines and isoalloxazines have been primarily explored as homogeneous catalysts, limiting their practical applicability with regard to product–catalyst separation and recyclability. Several immobilization approaches have been studied to circumvent this problem, including anchoring flavins to mesoporous silica,^32,46^ TiO_2_,^47^ BiOCl,^48^ or polydopamine.^49^ In these examples, however, the heterogeneous support seldom actively participates in the catalytic reactions.

Herein, we use an alloxazine building block in a bottom-up approach to construct a bio-inspired covalent organic framework that acts as a heterogeneous material with intrinsic photocatalytic activity. Direct comparison with similar homogeneous photocatalysts shows that this heterogenization approach not only leads to retention, but rather to the enhancement of the applicability towards “green” photocatalysis. To the best of our knowledge, this is the first report on a metal-free COF photocatalyst based on a bio-mimetic chromophore which is capable of selectively oxidizing benzylic alcohols to aldehydes using oxygen as the terminal oxidant.^50,51^

## Results and discussion

**Fig. 1:**
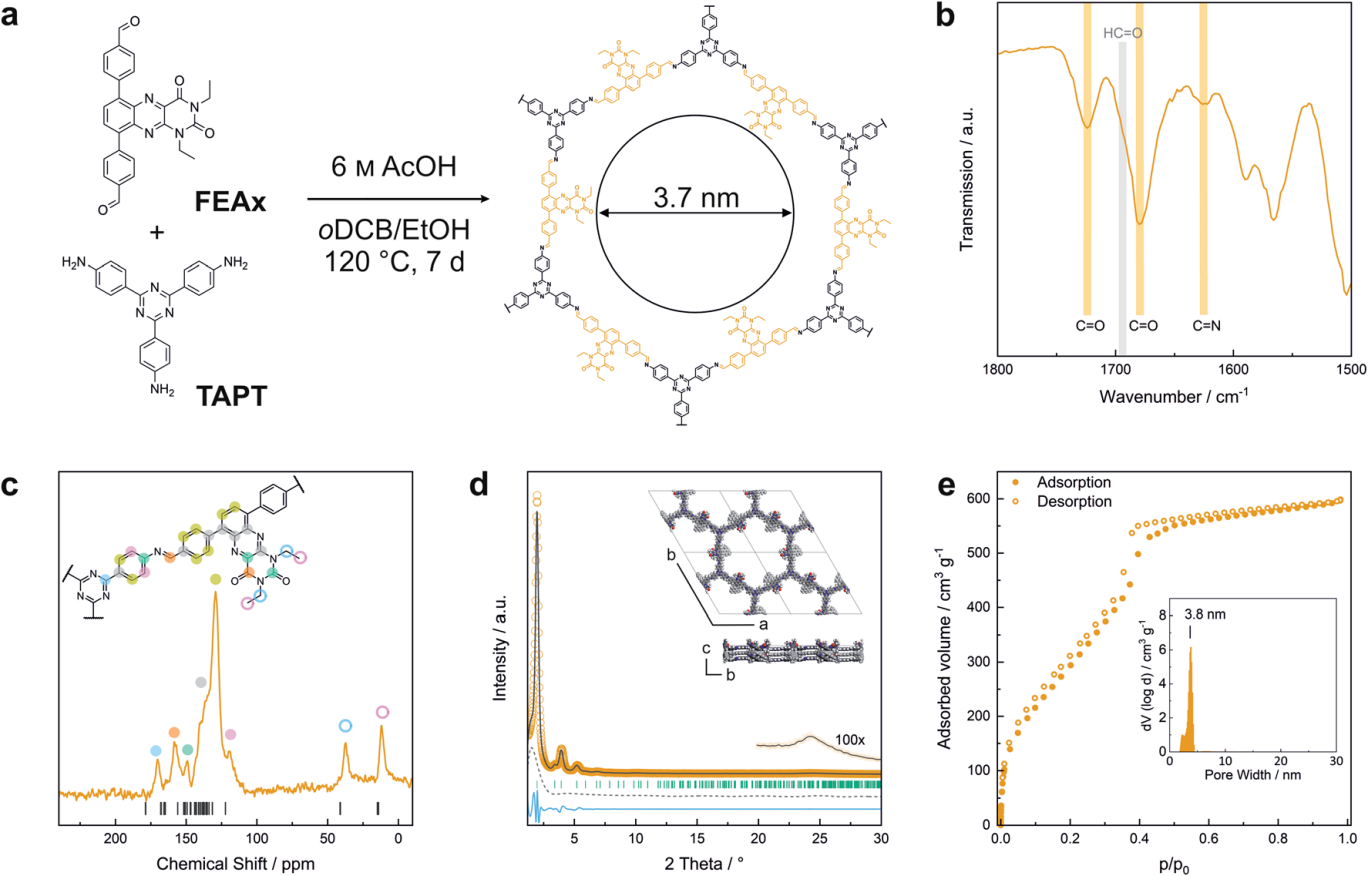
(a) Synthesis and molecular structure of FEAx-COF. (b) FTIR spectrum of FEAx-COF showing the presence of carbonyl and imine bands and the absence of an aldehyde band. (c) 13C ssNMR spectrum of FEAx-COF together with the corresponding assignments and calculated shifts. (d) XRPD pattern of FEAx-COF and illustration of the structural model used for refinement. The second COF layer is depicted in grey for better visualization. Experimental data shown in orange, Pawley refinement in grey, difference in blue, peak positions in green, and refined background as grey dashes. (e) Argon sorption isotherm of FEAx-COF at 87 K. Filled and open symbols represent the adsorption and the desorption branches, respectively. The inset shows the pore size distribution obtained from a QSDFT kernel for cylindrical pores.

FEAx-COF was synthesized by condensation of 1,3-diethyl-6,9-bis-(4-formylphenyl)alloxazine (FEAx) with 2,4,6-tris(4-aminophenyl)-1,3,5-triazine (TAPT) under solvothermal conditions (Fig. 1a). The FEAx building block was obtained from 4,7-dibromo-2,1,3-benzothiadiazole as described in the ESI.† The ethyl substituents at N-1 and N-3 (Fig. 1a) proved to be essential for the synthesis of FEAx-COF by providing both high solubility and photostability of the building block by preventing phototautomerism.^45,52,53^ Attempts to synthesize an analogous non-alkylated COF failed, potentially due to strong intermolecular hydrogen bonding (Fig. S5†). The successful condensation of FEAx and TAPT was confirmed by Fourier transform infrared (FTIR) spectroscopy, as evident from the appearance of the imine signal at 1624 cm^−1^ (*ν*_C=N (stretch)_) and concomitant disappearance of both amine (*ν*_N–H_ = 3200–3500 cm^−1^) and aldehyde (*ν*_C=O_ = 1692 cm^−1^) stretching vibrations of the starting materials (Fig. 1b and S7†). ^13^C solid-state nuclear magnetic resonance (ssNMR) further proved the successful condensation by an absence of aldehyde carbonyl ^13^C resonances at ∼190 ppm in the COF and the appearance of the imine ^13^C signal at 157 ppm (Fig. 1c).^54^ The distinct triazine carbon signal at 170 ppm, the signals from the ethyl groups at 12 and 37 ppm, together with the 1678 cm^−1^ and 1724 cm^−1^ bands in the FTIR spectra corresponding to the carbonyl groups of the alloxazine heterocycle prove the retention of the molecular structure of both FEAx and TAPT in the framework (Fig. S8†). Quantum-chemical calculations on the B97-2/pcsSeg-2//PBE0-D3/def2-TZVP level of theory corroborate the ^13^C NMR assignments (Fig. S30†).^55–60^ The ^1^H ssNMR spectrum of FEAx-COF shows aromatic protons around 7.6 ppm and two distinct aliphatic signals at 3.6 and 1.2 ppm corresponding to methylene and methyl groups, respectively (Fig. S8†). To understand the structural details and morphology of FEAx-COF, X-ray powder diffraction (XRPD), gas sorption, scanning electron microscopy (SEM), and transmission electron microscopy (TEM) analyses were performed. The XRPD pattern (Fig. 1d) shows an intense reflection at 2*θ* = 1.98°, assigned to the 100 plane (space group *P*3̄). In addition, a number of distinct reflections at 2*θ* = 3.41° (110), 3.93° (200), 5.20° (210), and 6.81° (220) are visible, together with a broad stacking reflection at 24.3°. Based on the geometrical considerations of the starting materials and their expected connectivity in the framework, a unit cell with the space group *P*3̄ was constructed, with cell parameters closely matching those obtained from Pawley refinement of the powder pattern (*R*_wp_ 8.0%). The obtained refined unit cell parameters are *a* = *b* = 51.84 Å, *c* = 7.06 Å, *α* = *β* = 90°, *γ* = 120°. An eclipsed stacking model accounting for only minimal relative layer offsets gave best fits between experimental and simulated data (Fig. S6†). Argon sorption analysis of FEAx-COF carried out at 87 K shows a type IV isotherm, which is typical for mesoporous materials (Fig. 1e).^61^ The Brunauer–Emmett–Teller (BET) surface area and pore volume were determined to be 1139 m^2^ g^−1^ and 0.76 cm^3^ g^−1^, respectively. A pore size distribution (PSD) was calculated from the sorption isotherm using the quenched solid density functional theory (QSDFT) kernel for argon at 87 K on carbon with cylindrical pores. The PSD shows a maximum at 3.8 nm, in agreement with the calculated pore size of 3.7 nm. PSD analysis thus further excludes the possibility of AB- (calculated pore size = 1.5 nm) and ABC-stacking (calculated pore size = 0.8 nm) of the layers (Fig. S6†).

SEM images of FEAx-COF show micrometer-sized, agglomerated spherical particles (Fig. S10†). TEM images visualize the hexagonal pores of the COF structure when viewed along the [001] zone axis (Fig. S11†) and Fast-Fourier Transform (FFT) analysis indicates a periodicity of 3.6 nm, in accordance with the experimental sorption and XRPD data.

With the synthesized COF in hand, we probed its activity as a sustainable catalyst for the selective photocatalytic oxidation of alcohols to aldehydes under aerobic, aqueous conditions. To determine if the COF is principally capable of such a reaction, the redox properties of FEAx-COF were investigated using cyclic voltammetry. The voltammogram of a COF-modified FTO working electrode shows an irreversible reduction peak with an onset potential (*E*_red_, onset) ≈ −0.73 V (Fig. S4†) *vs.* saturated calomel electrode (SCE). Using the experimentally obtained optical band gap (*E*_g,opt_) of 2.25 eV (Fig. S12†), the position of the conduction band (*E*_CB_) and the valence band (*E*_VB_) edges were estimated to be −3.97 eV and −6.22 eV *vs.* vacuum, respectively, following the empirical equations *E*_CB_ = −(*E*_red,onset_*vs.* SCE + 4.7) eV and *E*_VB_ = *E*_CB_ − *E*_g,opt_.^62–64^ Thus, both electron transfer to molecular oxygen (
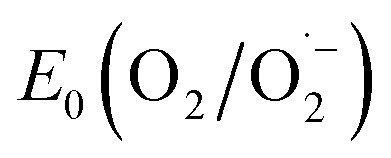
 = −0.33 V *vs.* NHE ≙ −0.57 *vs.* SCE),^65^ and oxidation of electron-rich organic substrates such as 4-methoxybenzyl alcohol (MBA, *E*_ox_ = 1.48 V *vs.* SCE) – a model reaction in flavin research^36,37,66,67^ – is thermodynamically feasible with FEAx-COF (*E*_VB_ 1.52 V *vs.* SCE).^68^

Indeed, irradiating the reaction mixture containing MBA and FEAx-COF in oxygenated acetonitrile/water with blue light (*λ*_max_ = 463 nm) for 17 h selectively oxidized MBA to 4-methoxybenzaldehyde (MBAld) with a yield of 44% (Table 1, entry 1).

**Table 1:** Photocatalytic oxidation of MBA by FEAx-COF

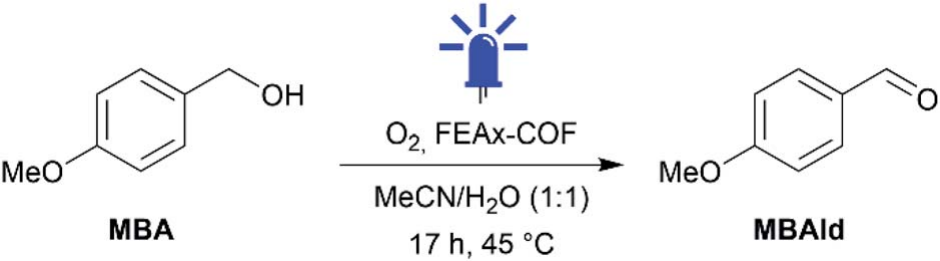		
Entry	Variation from standard conditions^a^	Yield^b^ (%)
1	—	44
2	No FEAx-COF	Traces
3	No irradiation	Traces
4	Under argon atmosphere	3
5	In water	22
6	In acetonitrile	70
7	Additional N(EtOH)_3_	17
8	Additional DABCO^c^	Traces
9	Additional *t*-BuOH	69

a Standard reaction conditions: 20 mM MBA, 1.5 mg FEAx-COF, 463 nm LEDs, MeCN/water (1 mL, 1 : 1), 45 °C, O_2_, stirring.

b Yield after 17 h determined *via* HPLC-MS.

c 1,4-Diazabicyclo[2.2.2]octan.

Interestingly, the photooxidation reaction proceeds with a high selectivity of 96% for MBAld, suggesting the capability of FEAx-COF as a selective photocatalyst. Notably, only 4-methoxybenzoic acid (MBAcid) was detected as the minor side product (Fig. S15†). Control experiments additionally confirmed that the presence of COF and irradiation of the reaction mixture are essential for the reaction to proceed (Table 1, entries 2 and 3). The presence of oxygen was also observed to be necessary for the reaction, indicating that O_2_ acts as a sacrificial electron acceptor (Table 1, entry 4).

We then tried to optimize the reaction yield of the photocatalytic system. The use of pure water and acetonitrile as solvents led to yields of 22% and 70%, respectively (Table 1, entries 5 and 6), which we attribute to the enhanced dispersibility of the rather hydrophobic COF in organic media, potentially enhancing the availability of active sites.

To gain mechanistic insights into the photocatalytic oxidation by FEAx-COF, a range of additional experiments was conducted. The addition of triethanolamine – N(EtOH)_3_ – or DABCO as competing electron donors drastically reduced the yield (Table 1, entries 7 and 8), hinting at direct oxidation of the benzylic alcohol by the photoexcited COF. As the presence of molecular oxygen is necessary for the reaction to proceed (*vide supra*), we tried to probe the possible formation and participation of the different reactive oxygen species, namely, singlet oxygen, hydroxyl or superoxide radicals in the photocatalytic transformation.^69^ Since neither the addition of hydroxyl radical scavenger *tert*-butanol (Table 1, entry 9), nor the absence of water (Table 1, entry 6) reduced the yield of MBAld, we expect hydroxyl radicals to only play a non-productive – if any – role in the catalytic cycle.

In order to detect possible singlet oxygen and superoxide species, we carried out electron paramagnetic resonance (EPR) spectroscopic measurements. When illuminating FEAx-COF in the presence of 5,5-dimethyl-1-pyrroline *N*-oxide (DMPO) as a spin-trap for the superoxide ion 
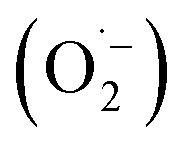
, we observed a 1 : 2 : 2 : 1 signal typical for the DMPO–OH adduct, formed by the decomposition of unstable DMPO–OOH, proving the presence and hence the formation of 
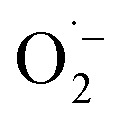
 during the catalytic cycle (Fig. S16†).^70^

When using 2,2,6,6-tetramethylpiperidine (TEMP) as the spin trapping agent for the detection of singlet oxygen, a 1 : 1 : 1 signal characteristic for (2,2,6,6-tetramethylpiperidin-1-yl)oxyl (TEMPO) was observed.^71^ Compared to the control measurement without illumination, the intensity of this signal increased after irradiation with blue light, suggesting that ^1^O_2_ is also generated alongside 
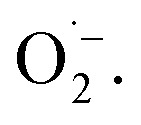
 Since TEMPO can also be formed in an alternative electron transfer reaction, we further corroborated the generation of singlet oxygen by oxidizing α-terpinene in the presence of FEAx-COF photocatalytically (Fig. S17†).^72,73^ The formation of ascaridole clearly proves the presence of singlet oxygen, and in accordance with the oxidative power of FEAx-COF we also detected *p*-cymene and other products of electron transfer reactions.

The productive role of singlet oxygen in the oxidation of MBA was tested by using deuterated solvents for the photocatalysis experiment with FEAx-COF. We could observe a slightly increased yield of 55% (*vs.* 44%) compared to standard reaction conditions when using a mixture of acetonitrile-*d*_3_ and D_2_O (Table S2,† entry 10), which we attribute to the prolonged lifetime of ^1^O_2_ in deuterated solvents.^74,75^ On the other hand, a decreased yield of 27% is observed in the presence of singlet oxygen scavenging sodium azide (Table S2,† entry 11). The retention of photocatalytic activity in the presence of a ^1^O_2_ scavenger also demonstrates that singlet oxygen is not the sole active oxygen species. This indicates the coexistence of 
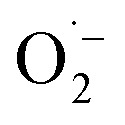
 and ^1^O_2_, which is also known for flavin^76^ and covalent triazine framework photocatalysts in aerobic oxidations, for example.^77–79^ However, we consider the generation of ^1^O_2_*via* energy transfer from photoexcited FEAx-COF to be negligible, since we did not encounter photooxidation of furfuryl alcohol even though furans are known for their reactivity towards ^1^O_2_ (Table S3,† entry 6).^80^ Instead, it is proposed that a second, but minor pathway for the oxidation of MBA to MBAld by singlet oxygen is enabled through electron transfer reactions with superoxide radicals, namely reoxidation of 
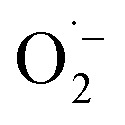
 to ^1^O_2_ by electron holes, or disproportionation of 
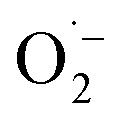
 to ^1^O_2_ and H_2_O_2_ (Fig. S18†).^20,81,82^

**Fig. 2:**
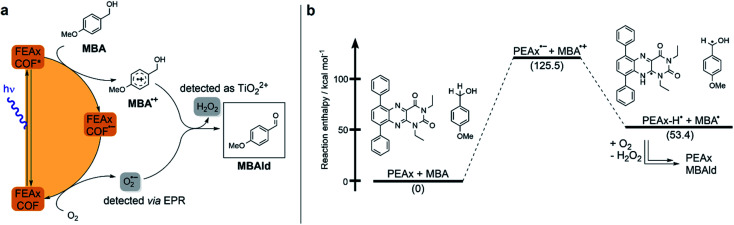
(a) Proposed mechanism for the photocatalytic oxidation of MBA by FEAx-COF. (b) Calculated reaction enthalpies for a possible pathway in the oxidation of MBA by model compound PEAx.

Based on these results and literature reports on aerobic photocatalysis with flavins,^67^ a plausible mechanism for the photooxidation of MBA by FEAx-COF can be compiled (Fig. 2a). The benzyl alcohol substrate is proposed to be oxidized by the photoexcited state of FEAx-COF, with the resulting radical anionic COF species reducing dioxygen to a superoxide radical. Through subsequent electron and proton transfers, 
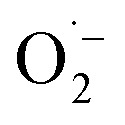
 and the substrate radical cation MBA˙^+^ eventually give the final products H_2_O_2_ and MBAld. Indeed, H_2_O_2_ was detected in the reaction filtrate using titanyl sulfate as the reagent, which led to the immediate formation of orange peroxotitanyl species (Fig. S28†).^83,84^

The reductive quenching of FEAx-COF in the mechanism elaborated above is in line with mechanistic investigations on MBA photooxidation by flavins.^66,67^ In addition, quantum-chemical calculations on PEAx (1,3-diethyl-6,9-diphenylalloxazine) as a molecular model system representative of the extended COF structure corroborate the proposed mechanism. The comparison of stabilization energies for the anionic and cationic state on the PBE0 D3/def2 TZVP level of theory (Table S4†) show the destabilization of the radical cation and the stabilization of the anion in the gas phase by +173.6 kcal mol^−1^ and −34.7 kcal mol^−1^, respectively. This indicates a reductive quenching of FEAx-COF to FEAx-COF˙^−^ as the more likely step than the oxidative quenching to FEAx-COF˙^+^.^85,86^

Furthermore, the reaction enthalpy for the photooxidation of MBA by FEAx-COF was estimated on the PBE0-D3/def2-TZVP level of theory with solvation effects being considered using the implicit solvation model COSMO with a value of 36.64 as the dielectric constant to represent acetonitrile (Table S5†).^87^ Following the mechanism proposed for FEAx-COF, PEAx is believed to be reduced to the radical anion PEAx˙^−^ after photoexcitation, while MBA is oxidized to MBA˙^+^ in return (Fig. 2b). The energy gained from the reduction is not enough to compensate for the formation of MBA˙^+^, rendering this single electron transfer endothermic by +125.5 kcal mol^−1^. Thus, considering the energy of the incident photons of 463 nm ≈ 62 kcal mol^−1^, a proton-coupled electron transfer (PCET) leading to PEAx-H˙ and MBA˙, with an associated reaction enthalpy of +53.4 kcal mol^−1^, seems more probable. Given the aerobic reaction conditions, it is expected that MBA˙ is further oxidized to MBAld either by a second photoexcited PEAx molecule, or by 
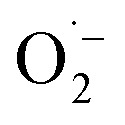
, the latter of which results from reoxidation of the intermediate semiquinone radical anion PEAx˙^−^ by dioxygen.^67^

**Chart 2:**
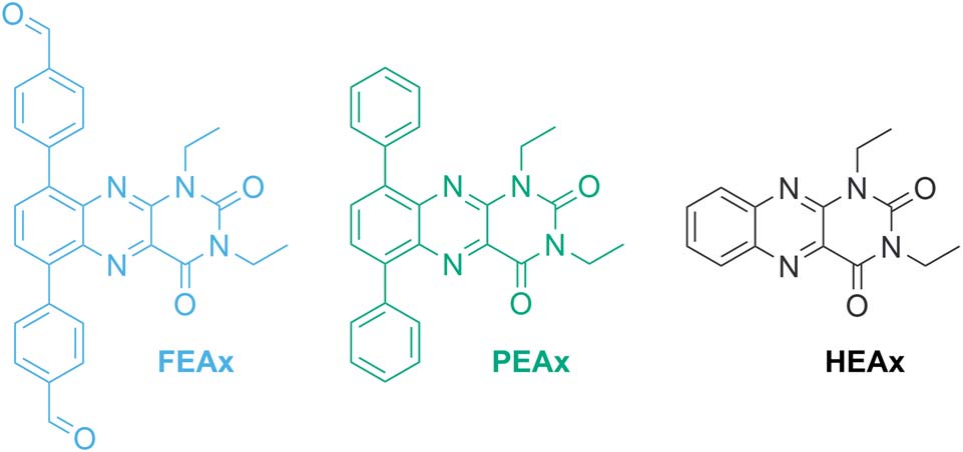
Molecular structure of alloxazine model compounds.

**Fig. 3:**
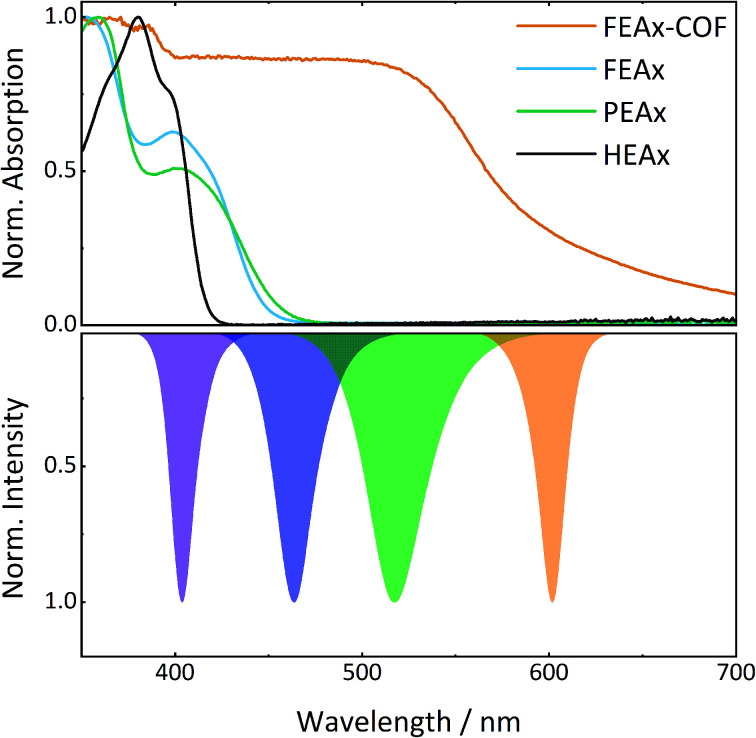
UV-vis spectra of model compounds and FEAx-COF (top) in comparison to LED emission spectra (bottom).

The photocatalytic activity of FEAx-COF in the oxidation of MBA was then compared to three different molecular alloxazine model systems – 1,3-diethylalloxazine HEAx, PEAx, and the FEAx linker (Chart 2). One important distinctive feature in the FEAx-COF system is the enhanced conjugation, which broadens its absorption profile and extends it up to 650 nm, with an absorption edge around 550 nm (Fig. 3). On the contrary, the light absorption of neither of the mentioned molecular alloxazines extends beyond the blue region of the visible spectrum.

Consequently, FEAx-COF surpasses the activity of the molecular compounds when illuminated with blue LEDs of 463 nm – especially when using acetonitrile as the solvent (Fig. S22†). In a 1 : 1 mixture of acetonitrile and water, HEAx and FEAx-COF perform similarly (Fig. S22†). However, when using low energy green light (517 nm), the reaction yield still remains at 20% with FEAx-COF, while no product formation is observed with HEAx, PEAx, or FEAx (Fig. S21†). Under illumination with orange LEDs, no oxidation takes place in either case.

To allow for sufficient light absorption by all four photocatalysts, this comparative study was conducted with violet LEDs (*λ*_max_ = 404 nm). The dissolved alloxazines HEAx, PEAx, and FEAx, gave yields of 78%, 39%, and 87% after 17 h, respectively (Table 2), which is either lower or in the range of the heterogeneous catalyst FEAx-COF (79%). To investigate possible photodegradation effects of the catalysts under prolonged illumination, we repeated this experiment after illuminating the oxygenated reaction mixtures for 72 hours prior to substrate addition.‡‡This “preillumination” experiment aims at simulating repeated photocatalytic cycles with the photocatalysts under investigation. Since the molecular alloxazines are homogeneous catalysts, actual cycling including catalyst recovery is impractical. To assess the remaining activity of the catalysts, we add MBA after 72 h – otherwise, it would have been completely oxidized to MBAld or even MBAcid. Interestingly, pre-illuminated HEAx and FEAx show clearly decreased yields of 40% and 55%, respectively, whereas PEAx (45%) and FEAx-COF (73%) do not show significant signs of lower activity (Table 2). This hints to higher photostability in the latter cases. In fact, UV-vis spectroscopy indicates more pronounced bleaching of the molecular alloxazines compared to FEAx-COF (Fig. S23†).

**Table 2:** Photocatalytic efficiency and photostability of FEAx-COF and model compounds in the oxidation of MBA under irradiation with violet light^a^

Entry	Catalyst	Yield^b^ (%)	Yield^b^^,^^c^ (%) after preillumination
1	HEAx	78	40
2	PEAx	39	45
3	FEAx	87	55
4	FEAx-COF	79	73

a Reaction conditions: 20 mM MBA, 1.5 mg FEAx-COF or 2 mM model compound, 404 nm LEDs, MeCN (1 mL), O_2_.

b Yield after 17 h determined *via* HPLC-MS.

c Samples illuminated prior to photocatalysis experiments (72 h, 404 nm, MeCN, O_2_).

When further assessing the photocatalytic activities of the molecular alloxazines under illumination with violet LEDs but in different solvents, we get significantly diverging reaction courses. For HEAx, we find higher turnover in a 1 : 1 acetonitrile/water mixture compared to pure acetonitrile, whereas FEAx and PEAx show decreased activity (Fig. S31†).

Inspired by these findings, we performed pulsed-field-gradient NMR experiments to determine the relative diffusion coefficients for FEAx and HEAx as a measure for their aggregation behaviour. According to the Stokes–Einstein equation, the diffusion coefficient is reciprocally related to the hydrodynamic radius of a diffusing species, which changes upon self-aggregation of the molecules.^88^ We find that HEAx exhibits a higher degree of aggregation in pure acetonitrile compared to a 1 : 1 acetonitrile/water mixture (Fig. S32†). On the contrary, FEAx shows higher aggregation in the aqueous solvent mixture. Although both molecular catalysts apparently show opposite aggregation behaviour in the respective solvents, a comparison with the photocatalytic yields of MBAld indicates an inverse correlation between aggregation and photocatalytic efficacy for both catalysts (Fig. S32†). In this regard, both FEAX and HEAX follow the behaviour of structurally related flavins as reported earlier by Dadová *et al.* and Feldmeier *et al.*^37,67^ Notably, this effect strongly reduces the yield of MBAld with the molecular catalysts FEAx (water) and HEAx (MeCN) to <5% when using blue LEDs, while FEAx-COF affords >20% of MBAld in either case (Fig. S22†). Incorporation of the alloxazine unit in the COF thus provides two benefits: suppressing solvent-induced aggregation while maintaining the accessibility of the active sites within the ordered porous structure.

The photocatalytic activity of FEAx-COF was further compared to a COF not comprising alloxazine chromophores. By using a terphenyl linker instead of FEAx for the construction of this reference material, we were able to obtain a COF with similar characteristics such as crystallinity, pore size, and surface area (Fig. S36†). However, the absence of alloxazine chromophores in the terphenyl COF leads to a hypsochromic shift of about 100 nm. After illumination with blue light for 24 h, FEAx-COF afforded 67% of MBAld, which is significantly higher compared to the terphenyl COF (15%). These results nicely illustrate that the photocatalytic activity of FEAx-COF mainly arises from the incorporation of alloxazine units.

After photocatalysis, the FEAx-COF sample was fully characterized to check for possible decomposition. As seen from the XRPD pattern, the framework crystallinity is largely, yet not completely retained, in line with the strongly oxidizing conditions during catalysis (Fig. S24†). Sorption analysis evidences the preservation of mesopores but reveals a significantly diminished surface area which we attribute to a partial amorphization of FEAx-COF. The FTIR and ssNMR data show the appearance of weak aldehyde signals which point to slight degradation effects, while the overall molecular connectivity and hence the structure of the framework remains largely unchanged (Fig. S25†). Further, SEM imaging illustrates the retention of the morphology of FEAx-COF (Fig. S26†).

In addition to its applicability for MBA photooxidation in different solvents and under varying irradiation wavelengths, FEAx-COF can also be used as a photocatalyst for an extended substrate scope. Since the reaction mechanism is based on an electron transfer from the substrate to the electron hole of FEAx-COF (*vide supra*), the scope is limited to substrates with oxidation potentials below *E*_VB_ (1.52 *vs.* SCE). Consequently, electron-poor alcohols such as 4-nitrobenzyl alcohol (*E*_ox_ = 2.84 *vs.* SCE), unsubstituted benzylic alcohol (*E*_ox_ = 1.94 *vs.* SCE), or furfuryl alcohol (*E*_ox_ = 1.73 *vs.* SCE) are not oxidized to the respective aldehydes in significant amounts (Table S3,† entries 1–6). On the other hand, FEAx-COF oxidizes 2-thiophenemethanol (*E*_ox_ = 0.72 *vs.* SCE) with yields similar to MBA (Table S3,† entries 5 and 7). Further, the photocatalytic activity of FEAx-COF is not limited to aromatic alcohols. Indeed, we could demonstrate the applicability of FEAx-COF also as a photocatalyst for the sulfoxidation of 2-methoxythioanisol (Table S3,† entry 10) and for the C–H oxidation of substrates such as xanthene and 4-methylanisol (Table S3,† entries 8 and 9).

## Conclusions

We report the first COF composed of photoactive, yet photostable alloxazine building blocks that can be used efficiently as a photocatalyst in aerobic oxidations. By virtue of not only anchoring alloxazines to, but rather incorporating them into the heterogeneous support, we obtain a COF that strongly absorbs visible light. Consequently, the photocatalytic efficacy of FEAx-COF equals or even exceeds the performance of a series of comparable molecular alloxazine photocatalysts, while simultaneously proving more stable. Notably, FEAx-COF catalyzes the oxidation of MBA even under illumination with low energy green light. More generally, its heterogeneous nature prevents disadvantageous aggregation of catalytic sites and allows for better product–catalyst separation and recycling. Overall, the construction of alloxazine COFs nicely illustrates the synthetic possibilities of the underlying reticular chemistry and broadens the scope of bio-inspired, metal-free heterogeneous photocatalysis.

## Data availability

Further data are stored on the DaRUS data repository (https://darus.uni-stuttgart.de/) and are accessible upon request.

## Author contributions

S. T. led the project, performed syntheses and experiments, and wrote the manuscript with input from L. G. and T. B. L. G. performed the electrochemical characterization of FEAx-COF, PFG-NMR diffusion measurements, and supported HPLC-MS measurements and mechanistic interpretation. G. S. and C. O. performed quantum-chemical calculations. K. I. M. and L. M. P. assisted in syntheses and experiments. F. H. and S. T. conceptualized FEAx-COF. B. V. L., T. B. and C. O. conceived and supervised the research, discussed the data, and co-wrote the paper. All authors read and commented on the manuscript.

## Conflicts of interest

There are no conflicts to declare.

## Supplementary Material

SC-012-D1SC04143F-s001

SC-012-D1SC04143F-s002
