# Supplementary material for: A flavin-inspired covalent organic framework for photocatalytic alcohol oxidation
Source: Chem Sci. 2021 Nov 15;12(45):15143–50. doi: 10.1039/d1sc04143f (PMC8612393; doi:10.1039/d1sc04143f)
Supplement: SC-012-D1SC04143F-s001 [file SC-012-D1SC04143F-s001.pdf]

Electronic Supplementary Material (ESI) for Chemical Science.
This journal is © The Royal Society of Chemistry 2021

Supporting Information

A Flavin-inspired Covalent Organic Framework for Photocatalytic Alcohol Oxidation

Stefan Trenker,[a,b,c] Lars Grunenberg,[a,b] Tanmay Banerjee,[d] Gökcen Savasci,[a,b,c,e] Laura M. Poller,[b] Katharina I. M. Muggli,[b] Frederik Haase,[e] Christian Ochsenfeld,[a,b,c,f] and Bettina V. Lotsch*,[a,b,c,f]

[a] Max Planck Institute for Solid State Research, Heisenbergstr. 1, 70569 Stuttgart, Germany.

[b] Department of Chemistry, University of Munich (LMU), Butenandtstr. 5-13, 81377 Munich, Germany.

[c] Center for Nanoscience, Schellingstr. 4, 80799 Munich, Germany.

[d] Department of Chemistry, Birla Institute of Technology and Science Pilani, Pilani Campus, Rajasthan - 333031, India.

[e] Karlsruhe Institute of Technology (KIT), IFG - Institute for Functional Interfaces, Hermann-von-Helmholtz-Platz 1, 76344 Eggenstein-Leopoldshafen, Germany.

[f] e-conversion Cluster of Excellence, Lichtenbergstr. 4a, 85748 Garching, Germany.

Table of Contents

Materials and Methods S2

Synthetic Procedures S7

Additional Data S10

Quantum Chemical Calculations S27

Crystal Structures S32

NMR Spectra S37

Materials and Methods

If not stated otherwise, all chemicals were obtained from commercial sources and used without further purification.

Physisorption analysis

Argon sorption measurements were performed at 87 K with a QUANTACHROME INSTRUMENTS Autosorb iQ MP. Samples of about 20 mg were preheated *in vacuo* (10^{-7} mbar) at 120 °C for 12 h. ASiQwin Version 3.01 was used for data analysis. Pore size distributions were evaluated using the carbon QSDFT kernel for cylindrical pores for both the adsorption and desorption branch. Expected pore sizes were derived from structural models in Materials Studio v6.0.0.

Mass spectrometry

Experiments were performed on a THERMO FINNIGAN MAT 90 or MAT 95 mass spectrometer using electrospray ionization (ESI). m/z values were calculated using PERKIN ELMER ChemDraw® Professional Version 16.0.0.82 (68).

Infrared spectroscopy

Infrared spectroscopy was conducted using a PERKIN ELMER Spektrum BX II FT-IR equipped with an ATR unit (Smith Detection Dura-Sample IIR diamond). Background correction was done before sample measurements. Depicted spectra are the mean of five scans.

Elemental analysis

Elemental analysis (C, H, N) was conducted on an ELEMENTAR vario EL using Helium as carrier gas.

UV-Vis

If not stated otherwise, UV-Vis spectra of molecular compounds were measured as solutions in acetonitrile on a Agilent Cary 60 UV-Vis spectrometer. Spectra of solid FEAx-COF were measured on a Jasco V-650 spectrophotometer.

Photoluminescence (PL)

Steady-state luminescence data was collected at RT using an EDINBURGH FLS980 spectrometer. The samples were excited by a housed 450 W xenon lamp whose light passed through a single grating (1800 l/mm, 250 nm blaze) Czerny-Turner monochromator and finally a bandwidth slit. The sample emission was passed through a double grating (1200 l/mm, 500 nm blaze) Czerny-Turner monochromator and detected by a peltier-cooled HAMAMATSU R928P photomultiplier tube.

X-ray powder diffraction (XRPD)

XRPD patterns were recorded at room temperature on a BRUKER D8 Discovery with Ni-filtered CuKα-radiation (1.5406 Å) and a position-sensitive LynxEye detector. Materials Studio v6.0.0 was used for structural modelling, XRD pattern simulations, and Pawley Refinement.

Single-crystal X-ray diffraction

Single-crystal X-ray diffraction data were collected at room temperature on a BRUKER D8 Venture diffractometer equipped with a rotating anode generator with Mo Kα radiation (λ = 0.71073 Å). The diffraction intensities were integrated using the SAINT software package and a multiscan absorption correction was applied with SADABS-2016/2 (BRUKER, 2016/2). The crystal structure was solved using intrinsic phasing (SHELXT)[1] and refined against F^2 by applying the full-matrix least-squares method (SHELXL)[2] using the software OLEX2[3]. Hydrogen atoms were inserted at idealized positions and refined using a riding model. All non-hydrogen atoms were refined anisotropically using full-matrix least-squares. Illustration of molecular structures with thermal ellipsoids was done with ORTEP-3 Version 2014.1.

Thermogravimetric analysis (TGA)

For TGA, the samples were filled into corundum crucibles. Measurements were carried out using a Netzsch STA 449 F5 Jupiter with an argon flow of 20 mL/min in a temperature range between 20 and 900 °C and a heating rate of 5 K/min. Data handling was performed with the Netzsch Proteus® software package.

Scanning electron microscopy

Images were recorded on a ZEISS Merlin SEM with an EHT voltage of 1.2 kV.

Transmission electron microscopy

The sample was slightly ground and distributed onto a holey carbon/copper grid. TEM was performed with a PHILIPS CM 30 ST microscope (300 kV, LaB6 cathode).

Electron paramagnetic resonance (EPR)

EPR spectra were measured with a BRUKER EMXnano. Experiments were conducted with 2.0 mg FEAx-COF in MeCN/water (1 mL, 1:1) after sonication for 5 min and oxygenation for 5 minutes. 0.1 mmol of the stated spin trap was added before illumination with blue LEDs (λ = 463 nm) for 5-15 minutes.

Supercritical CO_2 drying

COF samples were kept soaked in ethanol prior to supercritical CO_2 extraction on a Leica EM CPD300 critical point dryer with ethanol as exchange liquid.

Nuclear magnetic resonance spectroscopy

Solid-state nuclear magnetic resonance experiments on pristine FEAx-COF were performed on Bruker Neo NMR spectrometer operating at a ^{1}H Larmor frequency of 700 MHz using a 1.3 mm triple-resonance MAS probe and a spinning frequency of 55.55 kHz. The ^{13}C spectrum was measured as a direct-polarization ^{13}C experiment with a total of 4096 scans and a delay of 25 s. The COF sample was dried at 60 °C *in vacuo* prior to measurement.

Solid-state nuclear magnetic resonance experiments on FEAx-COF after catalysis were performed on Bruker Neo 600 MHz instrument using a 3.2 mm outer diameter ZrO_2 rotor and a BL3.2 MAS double resonance probe at a spinning frequency between 20 and 24 kHz. The ^{13}C spectrum was acquired by direct excitation accompanied by high power proton decoupling in a total of 3320 scans, and was subjected to background subtraction.

Spectra of soluble samples were recorded using a Bruker AV400TR or a Jeol Eclipse 400+ spectrometer. Chemical shifts are denoted on the scale in parts per million (ppm), calibrated to residual non-deuterated solvent (^{1}H-NMR: 7.26 for $CDCl_3$, 2.50 for DMSO-d_6) or solvent carbon resonances (^{13}C-NMR: $CDCl_3$: 77.16 for $CDCl_3$, 39.52 for DMSO-d_6). Multiplicities are denoted as: s = singlet, d = duplet, t = triplet, q = quartet, m = multiplet, or as a combination thereof. Spectra were analyzed and processed using MestReNova version 10.0.2-15465.

Diffusion coefficient determination

The pulsed field gradient NMR technique (PFG NMR) was applied to determine the relative diffusion coefficients D_{TMS}/D_M for M = FEAx or HEAx in acetonitrile-d_3 and acetonitrile-d_3/water-d_2 1:1 as a measure for the aggregation behaviour, as shown in Eq. 1.[4] Eq. 1 contains the diffusion coefficient D_t, numerical correction factors $c f_s$ and the hydrodynamic radius r_H of the sample (sa) and a standard (st). Tetramethylsilane (TMS) served as an internal standard for viscosity and temperature. The measurements were performed in 5 mm tubes on a Bruker Avance III 400 MHz spectrometer at 300 K with a stimulated-echo sequence[5] and default spoiler gradient (*diffSte* program, Bruker TopSpin). A gradient pulse with a lenght δ = 1 ms (*opt* shape) and a diffusion time Δ = 50 ms was applied. The gradient was varied linearly in 16 steps between 4.03 and 80.63 Gs/cm. Data processing was performed with the Bruker TopSpin 3.5 *Dynamics* module using automated peak picking and fitting of the signal Intensity *vs*. gradient *B* according to the Stejskal-Tanner equation (Eq. 2). Experiments were repeated at least three times with an appropriate number of scans (error bars represent the standard deviation).

$$\frac{D_t^{st}}{D_t^{sa}} = \frac{c^{sa} f_s^{sa} r_H^{sa}}{c^{st} f_s^{st} r_H^{st}} \qquad \text{(Eq. 1)}$$

$$\frac{I}{I_0} = e^{-BD} \qquad \text{(Eq. 2)}$$

Quantum-chemical calculations

Structures for all investigated molecular compounds were optimized on PBE0-D3/def2-TZVP level of theory.[6–9] Subsequent frequency calculations were performed on the same level of theory to ensure all minima to be true minima on the potential energy hypersurface. The same level of theory was used to calculate vertical radical stabilization energies as total energy differences between radical anionic, radical cationic, and neutral states of investigated model systems. Solvation effects have been considered using the implicit solvation model COSMO with a value of 36.64 as the dielectric constant to represent acetonitrile. Difference densities were calculated on the TD-PBE0/def2-TZVP//PBE0-D3/def2-TZVP level of theory, using the lowest vertical excitation. Calculations were performed using the Turbomole program package in version 7.3.[10]

NMR chemical shieldings were obtained on B97-2/pcsSeg-2 level of theory using the FermiONs++ program package.[11–13] NMR chemical shifts were obtained as differences of NMR chemical shieldings with respect to tetramethylsilane for carbon and hydrogen and nitromethane for nitrogen atoms on the same level of theory.

High pressure liquid chromatography with mass spectrometry (HPLC-MS)

High pressure liquid chromatography with mass spectrometry (HPLC-MS) was performed on an Agilent 1290 Infinity II LC system with an Agilent diode array detector (DAD; G7117B) connected to an Agilent InfinityLab LC/MSD XT single quadrupole mass spectrometer (G6135B) with a multimode ESI-APCI ionization source. Analysis of the combined signals was performed in Agilent ChemStation software.

Chromatographic separation was achieved on an Agilent Zorbax RRHD SB-C18 column (2.1x50 mm, 1.8 µm) at 40°C with mixtures of acetonitrile (MeCN), water and formic acid (FA), according to the solvent composition timetable (Table 1) and a total solvent flow of 0.7 mL/min. Absorption of the compounds was measured at 280±2 nm (reference: 325±10 nm) with DAD.

MS data was obtained using MM-APCI ionization (positive/negative 50:50) in scan mode for signals between 100-600 m/z.

Table S 1: Solvent composition timetable (HPLC-MS).

Time / min	Water / %	Water+1% FA / %	MeCN / %	MeCN+1% FA / %
0	72	8	18	2
2.00	45	5	45	5
2.01	4.5	0.5	85.5	9.5
2.50	4.5	0.5	85.5	9.5

Sample preparation

For a typical measurement, 25 µL of the sample suspension were diluted with acetonitrile/water 8:2 (975 µL) and filtered through a 13 mm 0.2 µm WWPTFE Pall Aerodisc® MS Syringe Filter. 1 µL of the filtrate was then injected.

Calibration

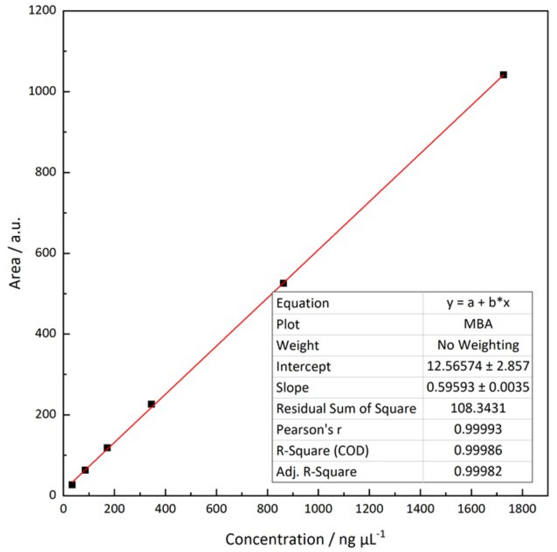

Figure S 1: The calibration line for MBA shows a linear response. Area is obtained by peak integration acquired by DAD-detector absorption at 280±2 nm (Reference 325±10 nm).

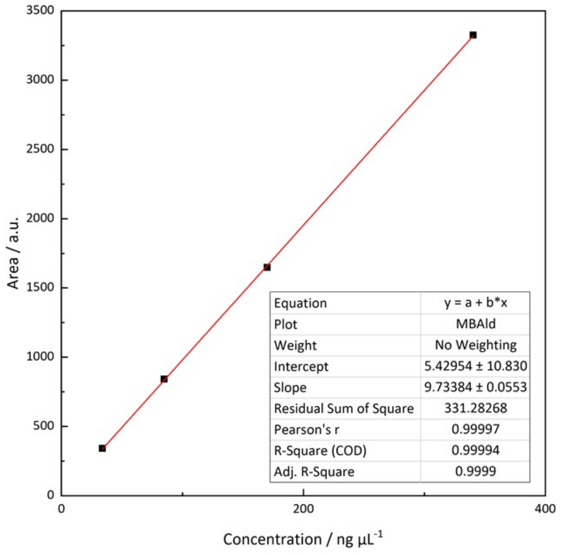

Figure S 2: The calibration line for MBAld shows a linear response. Area is obtained by peak integration acquired by DAD-detector absorption at 280±2 nm (Reference 325±10 nm).

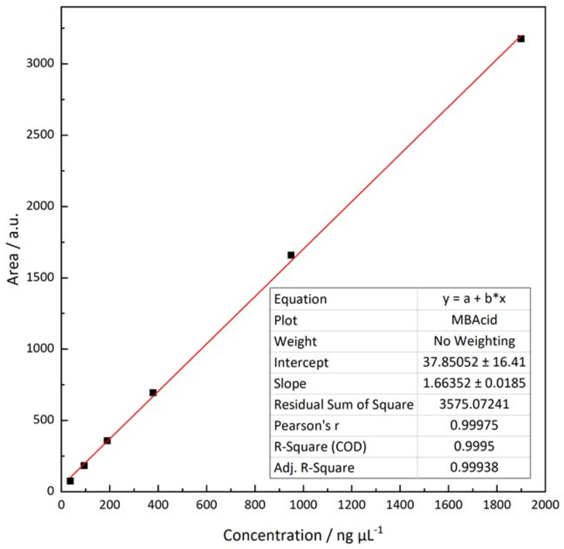

Figure S 3: The calibration line for MBAcid shows a linear response. Area is obtained by peak integration acquired by DAD-detector absorption at 280±2 nm (Reference 325±10 nm).

Photooxidations

For a typical photocatalytic experiment, 1.50 mg of FEAx-COF was suspended in 1 mL solvent in a 0.5–2.0 mL Biotage® microwave reaction vial and sonicated for 5 minutes in a water bath. In the case of molecular alloxazine photocatalysts, 2.2 µmol were used instead, which matches the expected amount of alloxazine units in 1.50 mg FEAx-COF. The reaction mixtures were oxygenated by oxygen bubbling for five minutes before addition of substrate and additional reagent. For reactions under Argon, the reaction mixture was instead degassed by four freeze-pump-thaw cycles after addition of substrate.

If not stated otherwise, reaction mixtures were illuminated inside a closed metal cylinder (diameter 10 cm, height 14.5 cm) with one meter of LED strip wrapped around the inside (60 diodes, 14.4 W/m, 12 V, 120° emission angle; see Figure S 27). The heat emission from the blue LEDs caused the reaction mixtures to heat up to 45 °C. In the case of differently colored LEDs, the photoreactors were cooled to room temperature using fans to account for potentially varying heat emission from the LEDs. If stated, this procedure was also applied to experiments with blue LEDs. Intensity measurements for every reactor were done at the same position central above the opening.

The yield and selectivity of the photocatalytic oxidation of 4-methoxybenzyl alcohol was determined *via* HPLC, using independent calibration curves for 4-methoxybenzyl alcohol, 4-methoxybenzaldehyde, and 4-methoxybenzoic acid. (Figure S 1-3) For other substrates (Table S 3), the conversion was determined *via* NMR spectroscopy.

Electrochemistry

Non-aqueous cyclic voltammetry experiments for FEAx-COF were conducted on a METROHM Autolab PGSTAT302N potentiostat with a COF-coated FTO working, a platinum wire counter and a non-aqueous Ag/$AgNO_3$ (0.01M) reference electrode, referenced to SCE using Ferrocene (Fc) as an internal standard ($E_{1/2,Fc}$ = 0.40 V vs. SCE).[14] COF films were grown solvothermally on FTO substrates (1 cm^2) by submersing them in the reaction mixture during solvothermal synthesis. Prior to the measurement, the electrochemical cell was purged with argon for 10 min.

Reduction onset potentials (E_{onset}) were extracted from the x-intercept of the linear fits in the voltammograms, according to a previous method.[15] Together with the optical bandgap ($E_{g,opt}$ = 2.25 eV) this value was used to estimate valence band (VB) and conduction band (CB) edges vs. the vacuum level[16] according to the following equations:

$E_{CB} = -(E_{onset\ vs.\ SCE} - E_{1/2,Fc} + 5.1)$ eV

$E_{VB} = E_{CB} - E_{g,opt}$

Energy levels of the material vs. vacuum level were calculated to E_{CB} = -3.97 eV and E_{VB} = -6.22 eV.

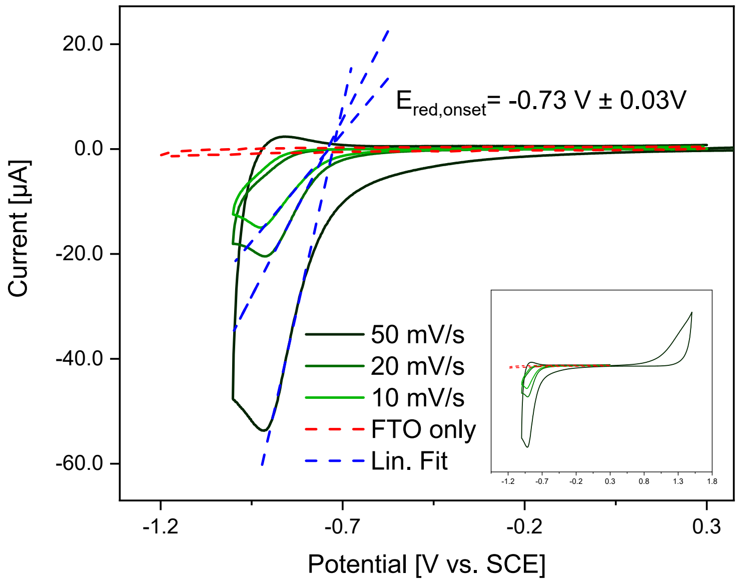

Figure S 4: Cyclic voltammograms of a COF-coated FTO working electrode in 0.1M NBu_4PF_6 in anhydrous acetonitrile at different scan rates. Red line shows background measurement without COF. Inset showing extended potential range.

Synthetic Procedures

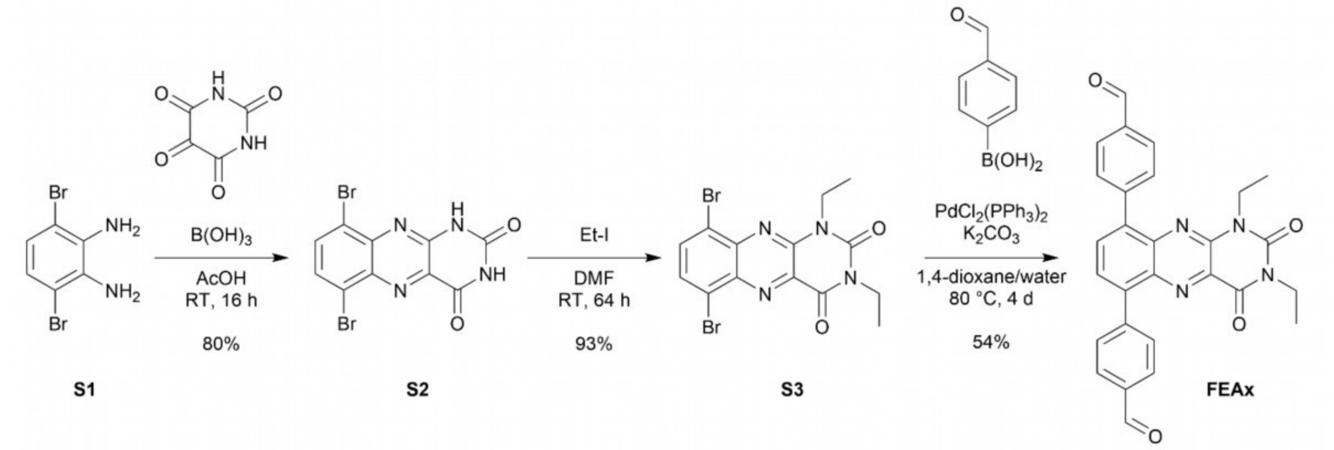

Scheme S 1: Synthetic route to the FEAx linker.

2,4,6-tris(4-aminophenyl)-1,3,5-triazine (TAPT) was synthesized according to the literature.[17]

3,6-dibromobenzene-1,2-diamine **S1** was synthesized according to the literature.[18]

Alloxazine was synthesized according to the literature.[19]

[1,1':4',1''-Terphenyl]-4,4''-dicarbaldehyde was synthesized according to the literature.[20]

Synthesis of 6,9-dibromoalloxazine **S2**

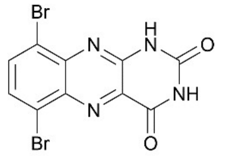

S2

Alloxan monohydrate (1.06 g, 6.50 mmol, 1.0 eq.) and boric acid (0.49 g, 7.80 mmol, 1.2 eq.) were dissolved in hot glacial acetic acid (48 mL). 3,6-Dibromobenzene-1,2-diamine **S1** (1.73 g, 6.50 mmol, 1.0 eq.) was dissolved in glacial acetic acid (12 mL) and added to the reaction mixture, resulting in a deep yellow solution. The mixture was stirred at room temperature for 16 h. The resulting solid was filtered off, washed with concentrated acetic acid (40 mL), and water (60 mL). Drying yielded 6,9-dibromoalloxazine **S2** (1.93 g, 5.18 mmol, 80%) as a yellow powder. ^{1}H NMR (400 MHz, DMSO-d_6) δ 12.27 (s, 1H), 11.89 (s, 1H), 8.17 (d, J = 8.2 Hz, 1H), 8.02 (d, J = 8.2 Hz, 1H) ppm. ^{13}C NMR (101 MHz, DMSO-d_6) δ 159.6, 149.9, 148.1, 141.1, 137.0, 136.2, 133.3, 131.6, 124.3, 120.5 ppm. MS (ESI-): m/z calc. for $C_{10}H_3Br_2N_4O_2^-$ (M-H$^+$): 370.86078; found: 370.86128. Elemental analysis calc. (%) for $C_{10}H_4Br_2N_4O_2$: C 32.29, H 1.08, N 15.06; found: C 32.32, H 1.20, N 14.86.

1,3-diethyl-6,9-dibromoalloxazine **S3**

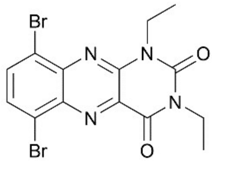

S3

6,9-dibromoalloxazine **S2** (0.69 g, 1.85 mmol, 1.0 eq.) and potassium carbonate (1.02 g, 7.40 mmol, 4.0 eq.) were suspended in anhydrous DMF (35 mL). Iodoethane (0.60 mL, 7.40 mmol, 4.0 eq.) was added dropwise and the reaction mixture was stirred at room temperature in the dark for 64 h. After slow addition of water (20 mL), the solvents were removed under reduced pressure. The residue was washed with water (30 mL) and dried *in vacuo* for 1 h, yielding 6,9-dibromo-1,3-diethylalloxazine **S3** (0.73 g, 1.71 mmol, 93%) as an ochre solid. ^{1}H-NMR (400 MHz, $CDCl_3$) δ 8.03 (d, J = 8.1 Hz, 1H, Ar-H), 7.91 (d, J = 8.1 Hz, 1H, Ar-H), 4.56 (q, J = 7.0 Hz, 2H, CH_2CH$_3$), 4.25 (q, J = 7.1 Hz, 2H, CH_2CH$_3$), 1.46 (t, J = 7.0 Hz, 3H, CH$_2$CH_3), 1.36 (t, J = 7.1 Hz, 3H, CH$_2$CH_3) ppm. ^{13}C-NMR (101 MHz, $CDCl_3$) δ 158.3, 149.7, 145.8, 142.0, 138.3, 136.7, 132.4, 130.9, 125.5, 122.0, 39.0, 38.2, 13.2, 12.7 ppm. MS (ESI+): m/z calc. for $C_{14}H_{13}Br_2N_4O_2^+$ (M+H$^+$): 428.93793; found: 428.93802. Elemental analysis calc. (%) for $C_{14}H_{12}Br_2N_4O_2$: C 39.28, H 2.83, N 13.09; found: C 37.71, H 2.36, N 13.21.

1,3-diethyl-6,9-bis-(4-formylphenyl)-alloxazine (FEAx)

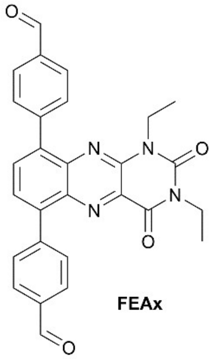

FEAx

6,9-dibromo-1,3-diethylalloxazine **S3** (858 mg, 2.00 mmol, 1.0 eq.), 4-Formylphenylboronic acid (930 mg, 6.02 mmol, 3.0 eq.), and potassium carbonate (1.66 g, 12.0 mmol, 6.0 eq.) were suspended in a mixture of 1,4-dioxane (35 mL) and water (1.8 mL). Bis(triphenylphosphine)palladium(II) dichloride (49.4 mg, 0.07 mmol, 0.03 eq.) was added, and the reaction mixture was stirred at 80 °C for 4 d. After cooling to room temperature, the mixture was filtered through a pad of celite and concentrated under reduced pressure. Purification by automated flash column chromatography (n-hexane/EtOAc 2:3 to 1:1) yielded FEAx (518 mg, 1.08 mmol, 54%) as a yellow solid. ^{1}H NMR (400 MHz, $CDCl_3$) δ 10.15 (s, 1H), 10.13 (s, 1H), 8.10 – 7.90 (m, 10H), 4.32 (q, *J* = 7.0 Hz, 2H), 4.22 (q, *J* = 7.1 Hz, 2H), 1.33 (t, *J* = 7.1 Hz, 3H), 1.31 (t, *J* = 7.0 Hz, 3H) ppm. ^{13}C NMR (101 MHz, $CDCl_3$) δ 192.2, 192.1, 159.0, 149.9, 144.6, 143.8, 143.3, 141.4, 141.1, 138.3, 137.9, 136.1, 135.9, 133.8, 131.7, 131.2, 129.8, 129.7, 129.5, 129.3, 38.3, 38.0, 13.2, 13.0 ppm. MP: 227 °C. HRMS (ESI+): m/z calc. for $C_{28}H_{23}N_4O_4^+$ (M+H$^+$): 479.17138; found: 479.17120. Elemental analysis calc. (%) for $C_{28}H_{22}N_4O_4$: C 70.28, H 4.63, N 11.71; found: C 69.66, H 4.46, N 11.25.

1,3-diethyl-6,9-diphenyl-alloxazine PEAx

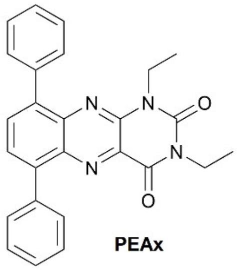

PEAx

In a Biotage® 20 mL microwave vial, 6,9-dibromo-1,3-diethylalloxazine **S3** (219 mg, 0.51 mmol, 1.0 eq.), Phenylboronic acid (187 mg, 1.50 mmol, 3.0 eq.), and potassium carbonate (417 mg, 3.02 mmol, 6.0 eq.), were suspended in a mixture of 1,4-dioxane (10 mL) and water (0.5 mL). Bis(triphenylphosphine)palladium(II) dichloride (14.2 mg, 0.02 mmol, 0.04 eq.) was added, and the reaction mixture was stirred at 80 °C for 3 d, then the heat was decreased to 50 °C for additional 19 h. After cooling to room temperature, the mixture was filtered through a pad of celite and concentrated under reduced pressure. Purification by automated flash column chromatography (*n*-hexane/EtOAc 19:1 to 1:1) yielded PEAx (518 mg, 1.08 mmol, 54%) as a yellow solid. ^{1}H NMR (400 MHz, $CDCl_3$) δ 8.02 (d, *J* = 7.5 Hz, 1H), 7.90 (d, *J* = 7.5 Hz, 1H), 7.87 – 7.82 (m, 2H), 7.76 – 7.70 (m, 2H), 7.58 – 7.42 (m, 6H), 4.33 (q, *J* = 7.0 Hz, 2H), 4.22 (q, *J* = 7.0 Hz, 2H), 1.33 (2 t, *J* = 7.0 Hz, 6H). ^{13}C NMR (101 MHz, $CDCl_3$) δ 159.3, 150.1, 144.1, 141.6, 141.5, 138.7, 138.3, 137.9, 137.5, 133.8, 131.1, 130.6, 129.2, 129.1, 128.4, 128.4, 128.1, 128.0, 38.2, 37.9, 13.3, 13.0. MP: 239 °C. MS (ESI+): m/z calc. for $C_{26}H_{23}N_4O_2^+$ [M+H$^+$]: 423.18155; found 423.18193. Elemental analysis calc. (%) for $C_{26}H_{22}N_4O_2$: C 73.92, H 5.25, N 13.26; found: C 72.30, H 5.22, N 12.55. Single-crystals were grown by slowly evaporating solutions of PEAx in $CHCl_3$.

1,3-diethyl-alloxazine HEAx

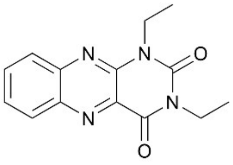

HEAx

To alloxazine (1.00 g, 4.70 mmol, 1 eq.) and potassium carbonate (2.60 g, 18.8 mmol, 4 eq.) in dry DMF (90 mL) was added iodoethane (1.52 mL, 18.8 mmol, 4 eq.). After stirring at room temperature in the dark for 18 h, the solvent was removed. The resulting solid was resuspended in water (60 mL), filtered off, and washed with water (2 x 40 mL). Drying *in vacou* yielded HEAx (1.16 g, 4.28 mmol, 91%) as a pale yellow powder. ^{1}H NMR (400 MHz, $CDCl_3$) δ 8.33 (d, *J* = 8.5 Hz, 1H), 8.03 (d, *J* = 8.6 Hz, 1H), 7.89 (t, *J* = 7.7 Hz, 1H), 7.75 (t, *J* = 7.7 Hz, 1H), 4.53 (q, *J* = 6.9 Hz, 2H), 4.26 (q, *J* = 7.0 Hz, 2H), 1.41 (t, *J* = 6.9 Hz, 3H), 1.36 (t, *J* = 7.0 Hz, 3H). ^{13}C NMR (101 MHz, $CDCl_3$) δ 159.6, 149.9,

145.0, 143.6, 140.1, 133.8, 131.0, 130.1, 129.1, 128.0, 38.2, 38.0, 13.3, 13.1 ppm. MP: 172 °C. MS (ESI+): m/z calc. for $C_{14}H_{15}N_4O_2^+$ (M+H$^+$): 271.11895; found: 271.11916. Elemental analysis calc. (%) for $C_{14}H_{14}N_4O_2$: C 62.21, H 5.22, N 20.73; found: C 62.12, H 5.00, N 20.43. Single-crystals were grown by slowly evaporating solutions of HEAx in $CHCl_3$.

6,9-bis-(4-formylphenyl)-alloxazine (FAx)

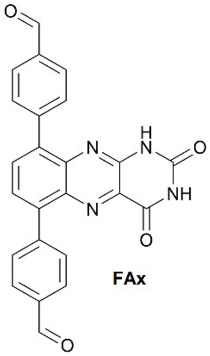

6,9-Dibromoalloxazine **S2** (0.40 g, 1.08 mmol, 1.0 eq.), 4-formylphenylboronic acid (0.50 g, 3.24 mmol, 3.0 eq.) and potassium carbonate (0.87 g, 6.48 mmol, 6.0 eq.) were suspended in 1,4-dioxane (28 mL) and degassed with three vacuum/argon cycles. Bis(triphenylphosphin)-palladium(II)chloride (38.3 mg, 0.054 mmol, 0.05 eq.) was added and the reaction mixture was heated to 80 °C for 21 h and subsequently to 90 °C for another 46 h under inert gas atmosphere. After cooling to ambient temperature, water (40 mL) was added and the solvent was removed. The product was filtered off, washed with water (100 mL), and dried in the desiccator overnight. 6,9-bis-(4-formylphenyl)-alloxazine (0.42 g, 0.99 mmol) was obtained as an impure brown solid. ^{1}H-NMR: (270 MHz, DMSO-d_6) δ 10.13 (s, 1H), 10.11 (s, 1H), 8.06-7.90 (m, ~15H), 7.72 (d, *J* = 7.5 Hz, 1H), 7.65 (d, *J* = 8.1 Hz, 1H) ppm. MS (ESI-): m/z calc. for $C_{24}H_{13}O_4N_4$ (M$^-$): 421.09423; found: 421.09457. Elemental analysis calc. (%) for $C_{24}H_{13}O_4N_4$: C 68.24, H 3.34, N 13.26; found: C 51.29, H 3.10, N 10.78.

FEAx-COF Synthesis

A Biotage® 5 mL microwave vial was charged with FEAx (27.0 mg, 0.056 mmol, 1.5 eq.) and TAPT (13.3 mg, 0.038 mmol, 1.0 eq.). The vial was temporarily sealed with a rubber septum and flushed three times *via* vacuum/argon cycles. 1,2-dichlorobenzene (1.8 mL) and ethanol (0.15 mL) were added, and the reactants were suspended *via* sonication for 5 minutes. The suspension was degassed *via* three vacuum/argon cycles. Aqueous acetic acid (100 µL, 6M) was added, the vial was sealed with a crimp cap and heated in a muffle furnace at 120 °C for 7 d. After cooling to room temperature, the combined solids of five parallelized batches were filtered off and washed with DMF (50 mL), THF (50 mL), DCM (30 mL), acetone (100 mL), and MeOH (30 mL). Soxhlet extraction with MeOH overnight followed by supercritical CO_2 drying yielded FEAx-COF (143 mg, 75%) as an orange powder. Elemental analysis calc. (%) for $C_{126}H_{90}N_{24}O_6$: C 74.32, H 4.45, N 16.51; found: C 68.82, H 4.37, N 14.92.

Syntheses in other solvent mixtures such as mesitylene/1,4-dioxane 7:2, 1,2-dichlorobenzene/2-propanol 7:6, and 1,2-dichlorobenzene/*n*-butanol 1:1, or in 1,2-dichlorobenzene/ethanol with varying acid concentrations gave less crystalline COF.

FAx-COF Synthesis

A Biotage® 5 mL microwave vial was charged with FAx (25.6 mg) and TAPT (10.8 mg). The vial was temporarily sealed with a rubber septum and flushed three times *via* vacuum/argon cycles. 1,2-dichlorobenzene (985 µL) and ethanol (85 µL) were added and the reactants were suspended *via* sonication for 3 minutes. The suspension was degassed *via* three vacuum/argon cycles. Aqueous acetic acid (30 µL, 6M) was added, the vial was sealed with a crimp cap and heated in a muffle furnace at 120 °C for 3 d. After cooling to room temperature, the solid was filtered off and washed with DMF (10 mL), THF (10 mL), DCM (10 mL). The product was dried in a desiccator overnight.
The BET surface area was determined to be 21.4 m²/g. XRPD patterns for this batch of FAx-COF and samples synthesized in different solvents are shown in Figure S 5.

Terphenyl-COF Synthesis

A Biotage® 5 mL microwave vial was charged with [1,1':4',1''-Terphenyl]-4,4''-dicarbaldehyde (16.1 mg, 0.056 mmol, 1.5 eq.) and TAPT (13.3 mg, 0.038 mmol, 1.0 eq.). The vial was temporarily sealed with a rubber septum and flushed three times *via* vacuum/argon cycles. 1,2-dichlorobenzene (1.8 mL) and ethanol (150 µL) were added and the reactants were suspended *via* sonication for 5 minutes. The suspension was degassed *via* three vacuum/argon cycles. Aqueous acetic acid (27 µL, 6M) was added, the vial was sealed with a crimp cap and heated in a muffle furnace at 120 °C for 7 d. After cooling to room temperature, the combined solids of three parallelized batches were filtered off and washed with DMF (30 mL), THF (20 mL), acetone (50 mL), and MeOH (20 mL). Soxhlet extraction with MeOH overnight followed by supercritical CO_2 drying yielded Terphenyl-COF (44.3 mg, 54%) as a yellow solid.

Additional Data

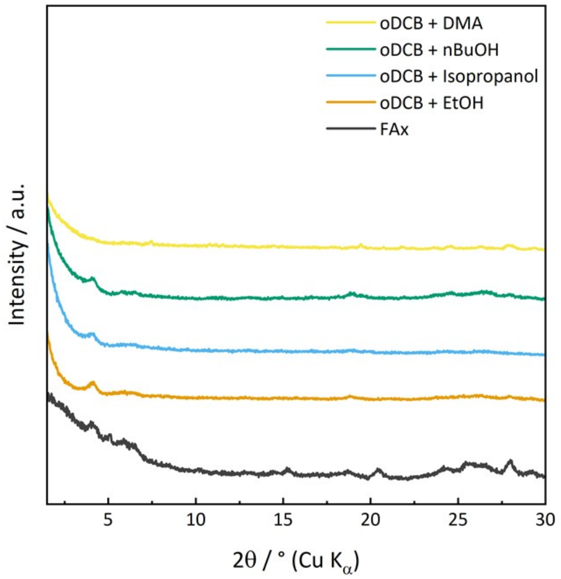

Figure S 5: XRPD data for the synthesis of non-ethylated FAx-COF in various solvents in comparison to the FAx linker.

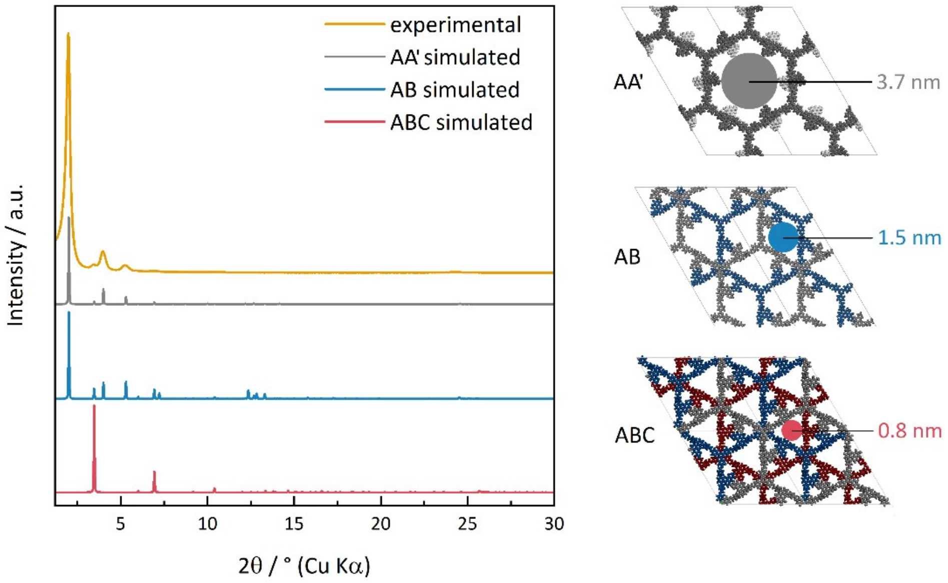

Figure S 6: Experimental powder diffractogram of FEAx-COF in comparison to simulated patterns of different stacking models. Colored circles illustrate the expected pore size for each stacking model.

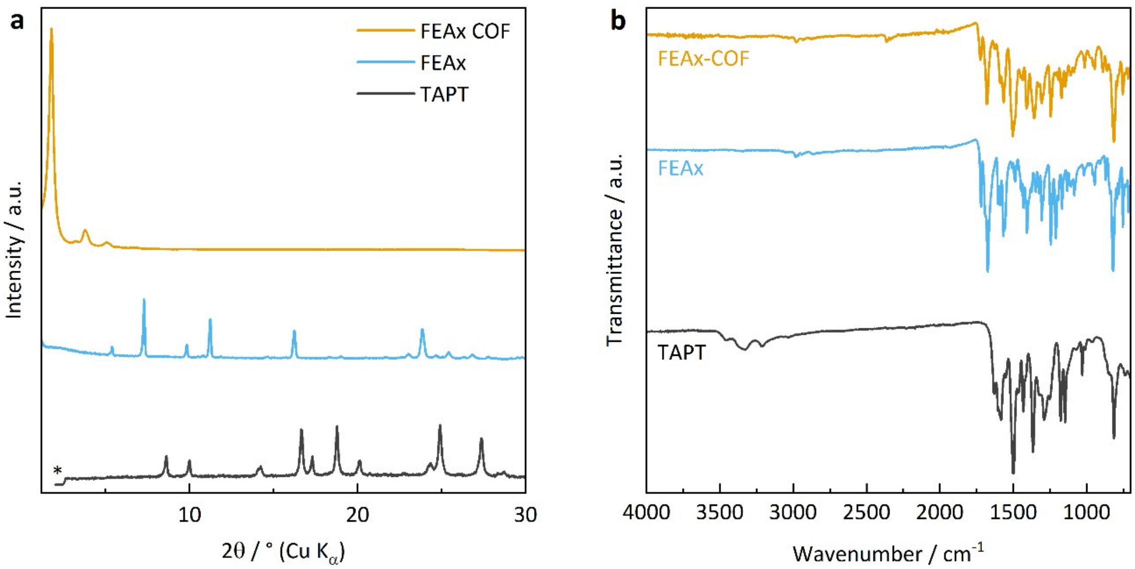

Figure S 7: Powder diffractogram (a) and FTIR spectrum (b) of FEAx-COF in comparison with its molecular linkers. The asterisk indicates data collection blocked by a beam stop.

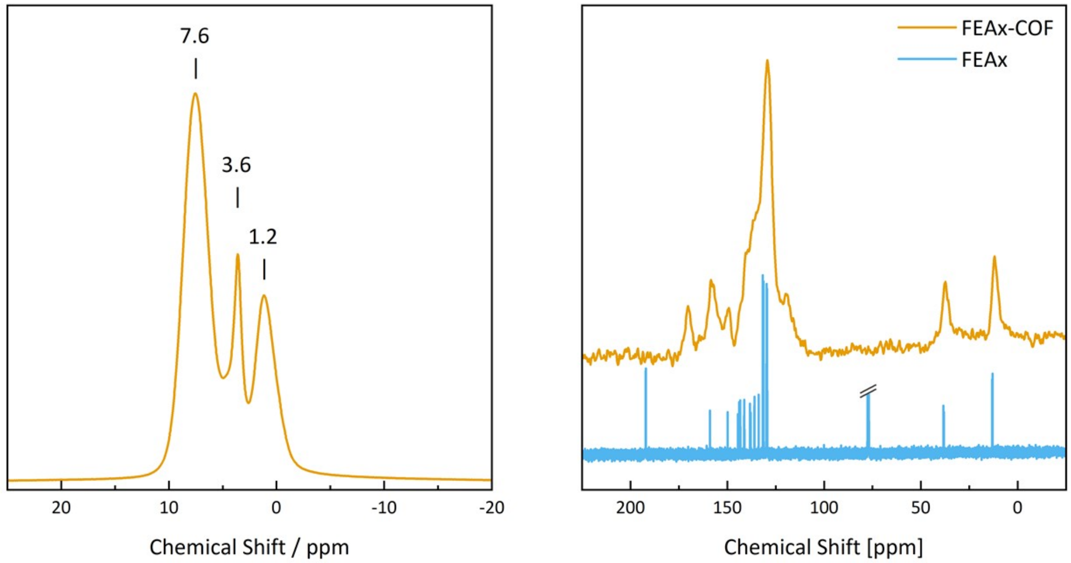

Figure S 8: ^{1}H-ssNMR spectrum of FEAx-COF (left) and ^{13}C-NMR spectra of FEAx-COF in comparison to the FEAx linker (right). FEAx-COF measured as a solid, FEAx linker measured as a solution in $CDCl_3$. Residual solvent peak at 77.16 ppm abbreviated for clarity.

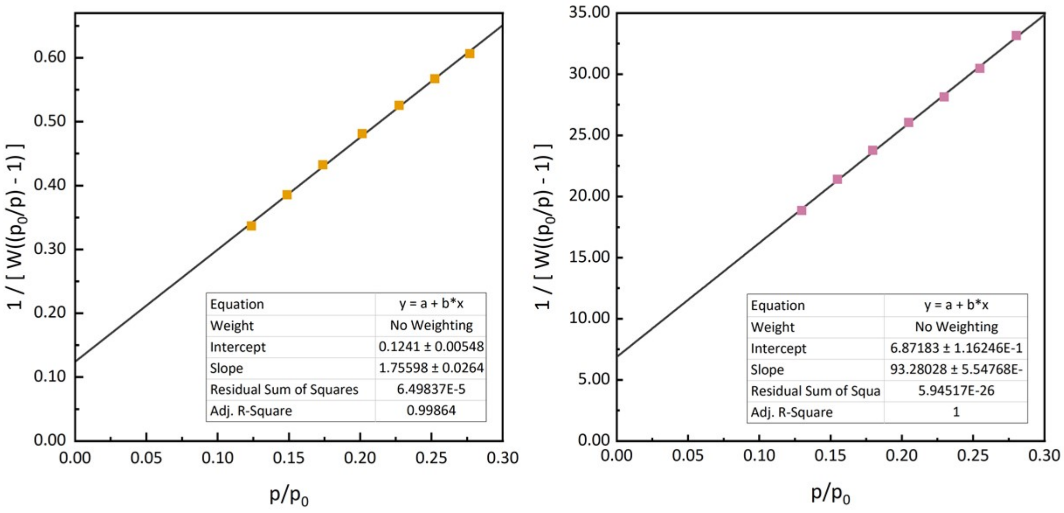

Figure S 9: BET plot for FEAx-COF (left) and non-ethylated FAx-COF (right).

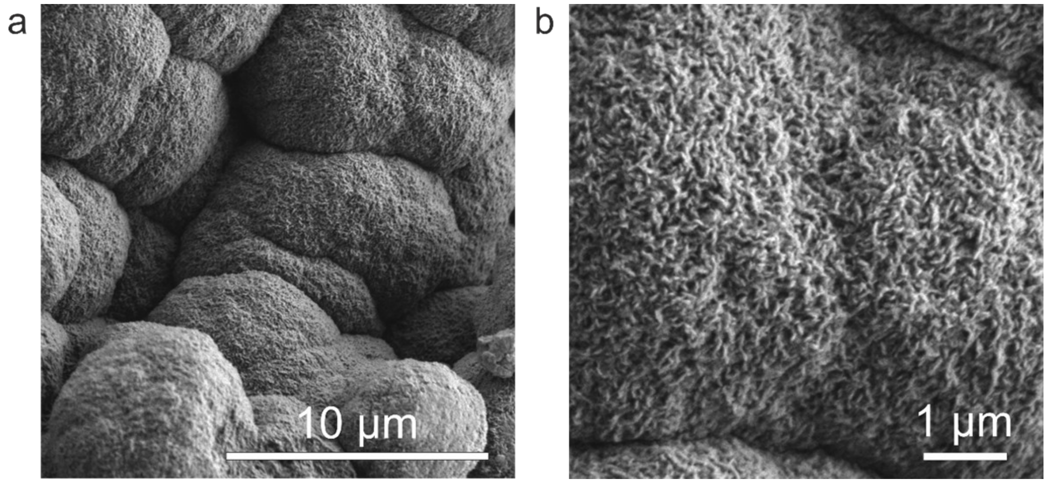

Figure S 10: SEM images of FEAx-COF showing aggregated, micrometer-sized particles with fringed surfaces. Image (b) shows a zoom onto a particle shown in (a).

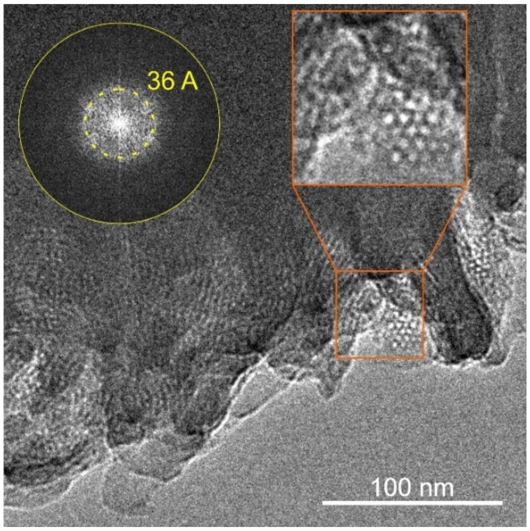

Figure S 11: TEM image of FEAx-COF. The magnification illustrates the hexagonal arrangement of the mesopores. The FFT of the TEM image underlines the periodicity of 3.6 nm.

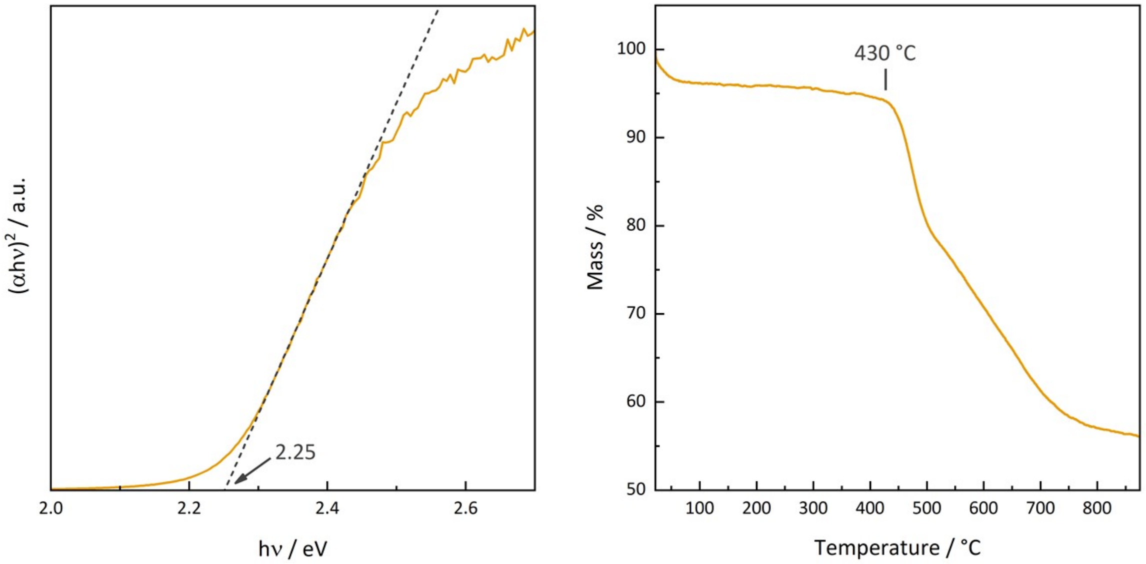

Figure S 12: Tauc plot (left) and thermal analysis under argon (right) for FEAx-COF.

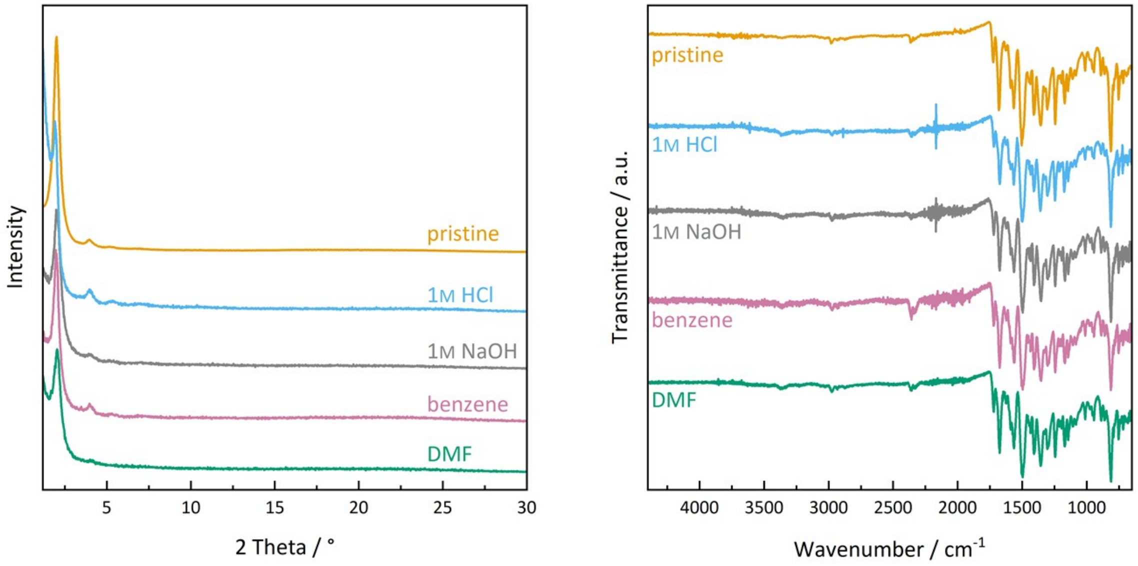

Figure S 13: XRPD (left) and IR data (right) for stability tests with FEAx-COF. COF suspended in the stated solvent and stirred for 24 h on a platform shaker at 150 rpm, then washed and dried ($scCO_2$).

Table S 2: Photocatalytic oxidation of MBA by FEAx-COF.

Entry	Variation from Standard Conditions[a]	Comment	17 h Yield / %	17 h Select. / %	24 h Yield / %	24 h Select. / %
1	none		44	97	67	96
2	no FEAx-COF		traces	100	traces	100
3	no irradiation		traces	100	traces	100
4	under Argon atmosphere		3	100	4	100
5	in water		22	94	25	93
6	in acetonitrile		70	94	84	92
7	additional $N(EtOH)_3$	competing e^- donor	17	98	-[d]	-[d]
8	additional DABCO[b]	competing e^- donor / $^1O_2$ scavenger	traces	100	1	100
9	additional *t*-BuOH	$OH^•$ scavenger	69	96	-[d]	-[d]
10	in MeCN-d_3/D_2O	longer $^1O_2$ lifetime	55	99	79	99
11	additional NaN_3	$^1O_2$ scavenger	27	95	-[d]	-[d]
12	additional BQ[c]	$O_2^{•-}$ scavenger	42	94	-[d]	-[d]
13	additional BQ,[c] no FEAx-COF	$O_2^{•-}$ scavenger	54	84	56	85
14	additional $CuCl_2$	competing e^- acceptor / $O_2^{•-}$ scavenger	9	100	13	100

[a] Standard reaction conditions: 20 mM MBA, 1.5 mg FEAx-COF, 463 nm LEDs, MeCN/water (1 mL, 1:1), O_2, stirring. Additional reagent: 0.02 mmol. Yield and selectivity determined *via* HPLC-MS. [b] 1,4-Diazabicyclo[2.2.2]octan. [c] 1,4-Benzoquinone. [d] Not measured.

The addition of selective scavengers to reaction mixtures containing FEAx-COF and MBA was used to pinpoint the reaction mechanism, for example by identifying reactive oxygen species. In addition to the experiments already discussed in the main text (Table S 2, entries 1-11), we also employed a number of potential superoxide radical scavengers, namely 1,4-benzoquinone (BQ) and $CuCl_2$. When adding BQ to the reaction mixture, the yield of MBAld after 17 h remains unchanged compared to the standard conditions (Table S 2, entries 1 and 12). However, even when illuminating a solution of BQ and MBA not containing FEAx-COF, we still get a yield of 54% (Table S 2, entry 13). Closer inspection of the course of MBAld formation indicates that addition of BQ leads to undesired alcohol oxidation *via* pathways not involving FEAx-COF photocatalysis.[21] Therefore, scavenging experiments with BQ are not applicable to deduce the role of $O_2^{•-}$ in our case. On the other hand, when using $CuCl_2$ as $O_2^{•-}$ scavenger[22] / competing electron acceptor,[23] we see a clear decrease in activity for FEAx-COF, which we attribute to a reduced generation of $O_2^{•-}$.

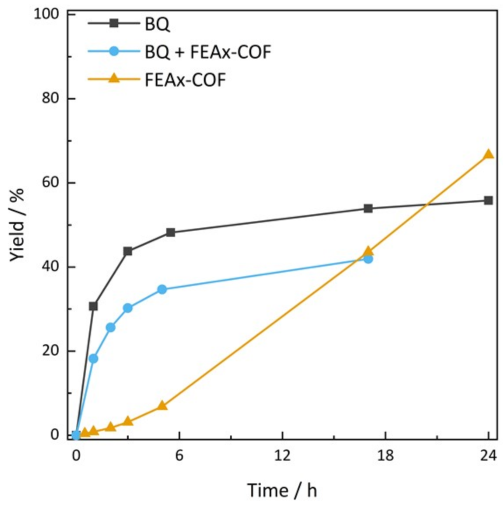

Figure S 14: Photocatalytic oxidation of MBA with benzoquinone as superoxide scavenger. Reaction conditions: 20 mM MBA, 1.5 mg FEAx-COF, 0.02 mmol BQ, 463 nm LEDs, MeCN/water (1 mL, 1:1), O_2, stirring. Yield determined *via* HPLC-MS.

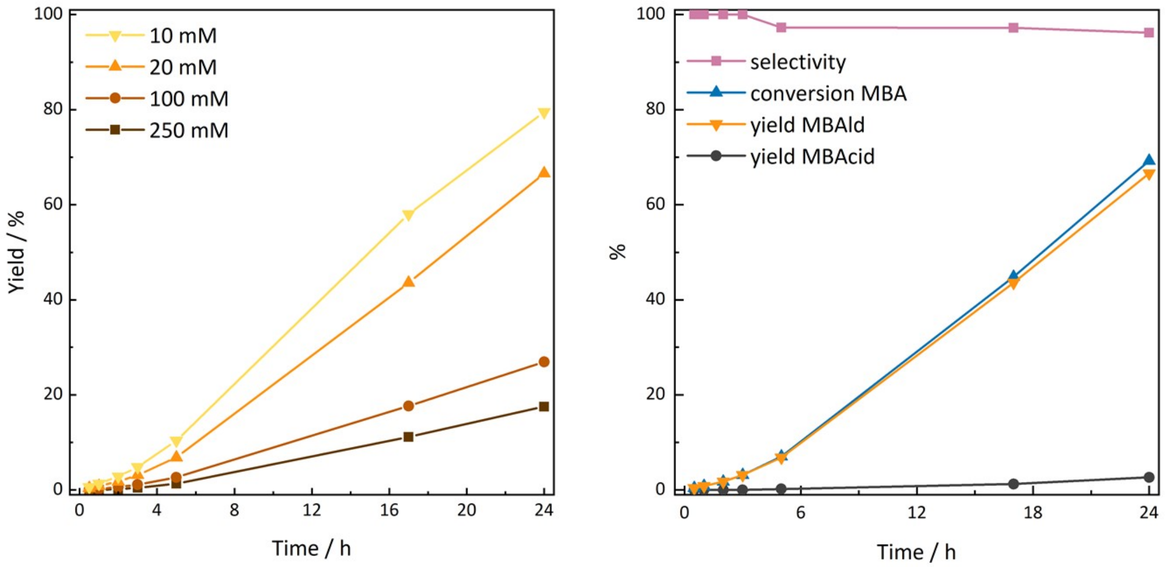

Figure S 15: Photocatalytic activity of FEAx-COF in MBA solutions with varying concentrations (left). Detailed reaction course for the 20 mM experiment as an example for a typical photocatalytic experiment with FEAx-COF (right). Reaction conditions: 1.5 mg FEAx-COF, 463 nm LEDs, MeCN/water (1 mL, 1:1), O_2, stirring. Yield, selectivity, and conversion determined *via* HPLC-MS.

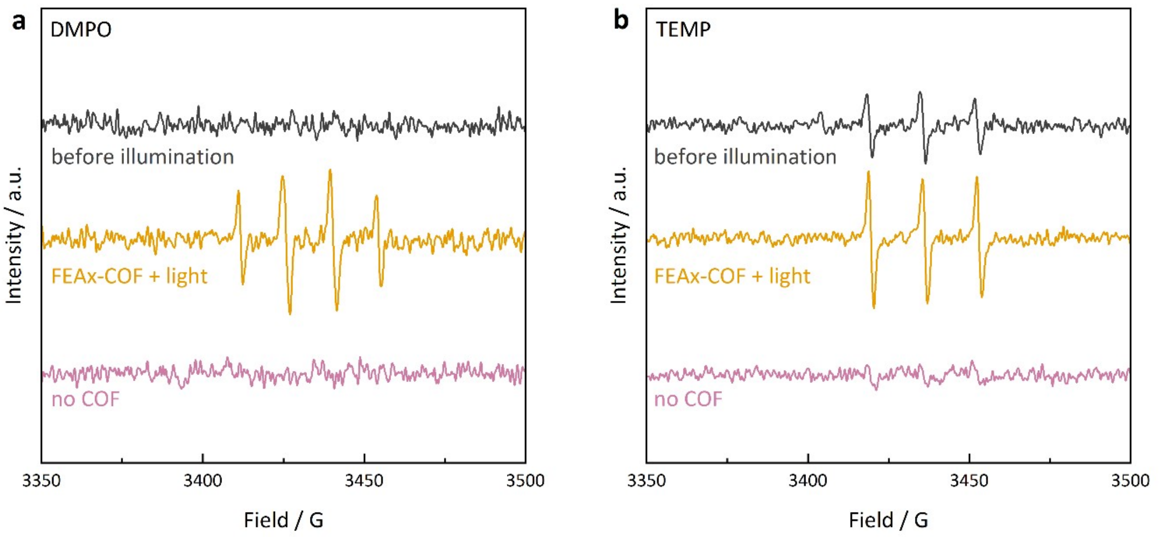

Figure S 16: Detection of reactive oxygen species with EPR spin-traps DMPO for superoxide (a) and TEMP for $^1O_2$ (b), respectively.

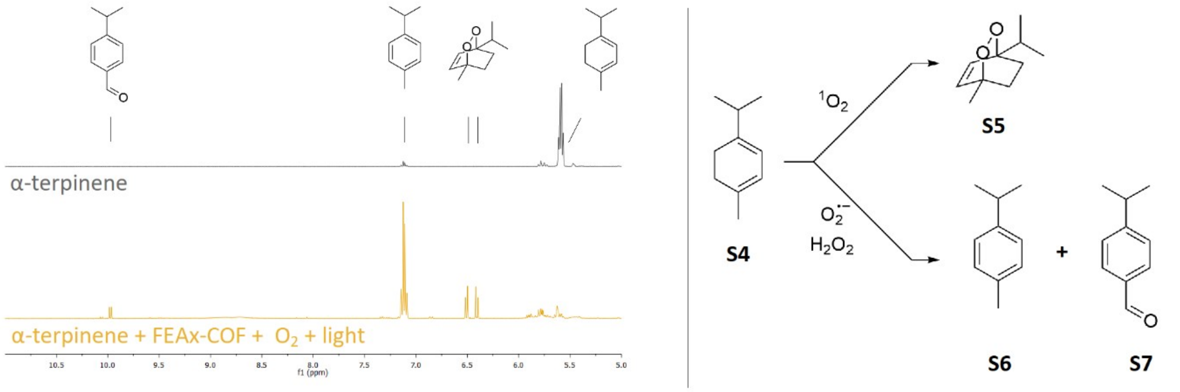

Figure S 17: The oxidation of α-terpinene **S4** by FEAx-COF shows both ascaridole **S5** from reaction with $^1O_2$ as well as oxidation products *p*-cymene **S6** and aldehyde **S7**. Reaction conditions: 1.5 mg FEAx COF, 50 mM α-terpinene in MeCN-d_3, O_2, RT, 404 nm LED irradiation.

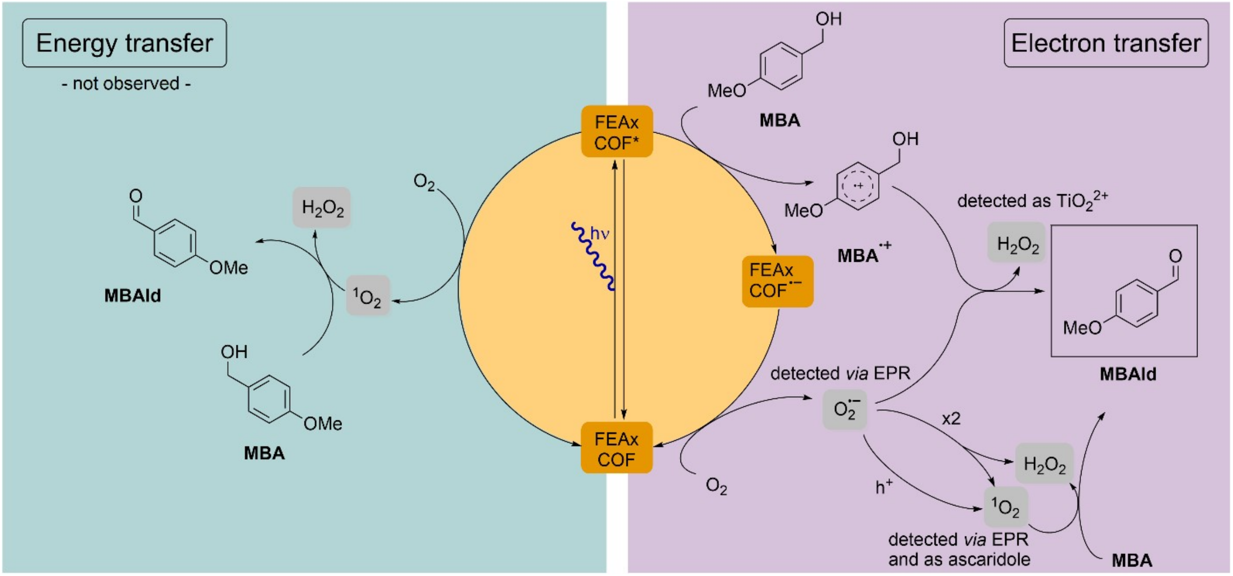

Figure S 18: Possible catalytic cycles for the photooxidation of MBA by FEAx-COF ν *via* energy transfer (left) and electron transfer (right).

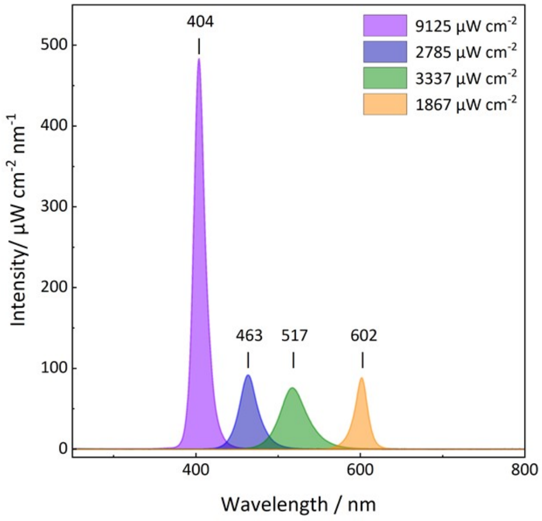

Figure S 19: Emission spectra of LED photoreactors used in this study. The annotations and the legend correspond to λ_{max} and integrated intensity, respectively.

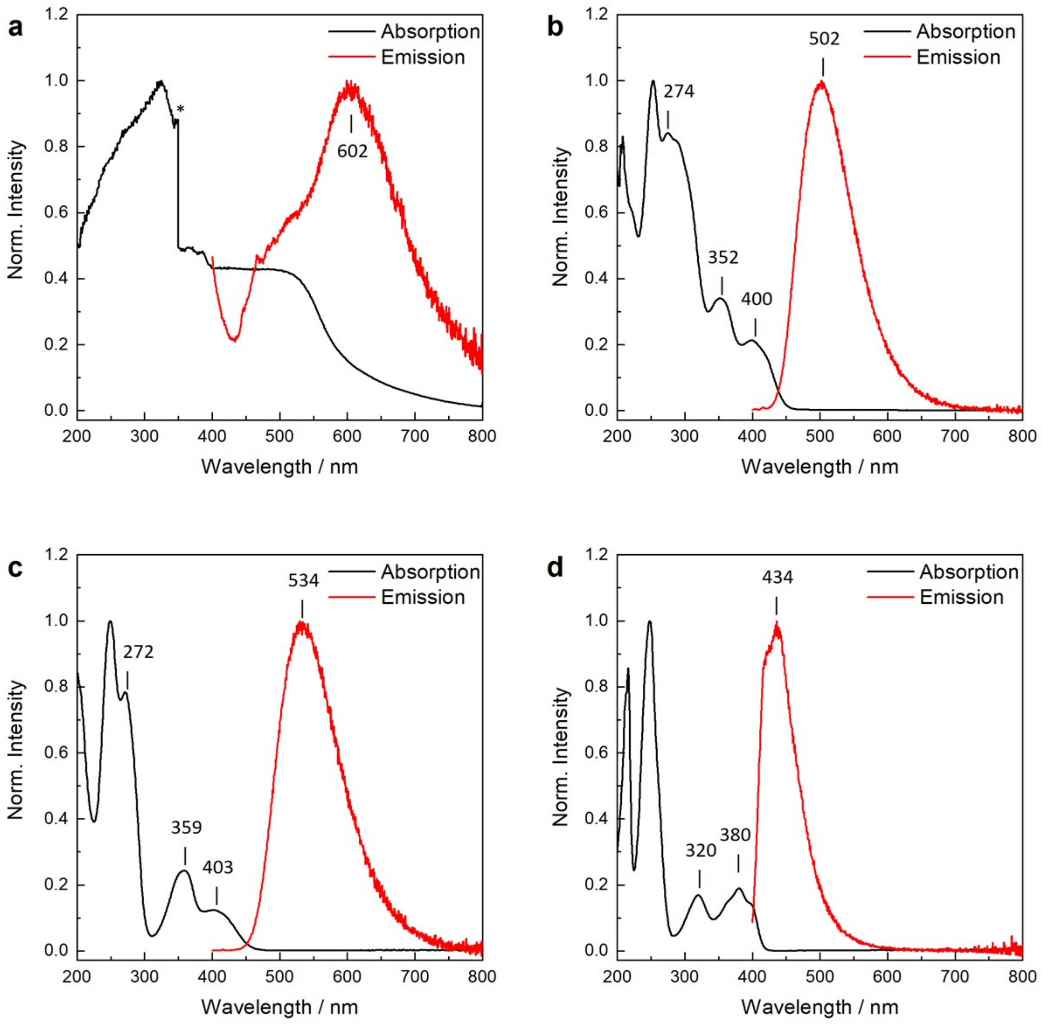

Figure S 20: Absorption and emission spectra of FEAx-COF (a) and the model compounds FEAx (b), PEAx (c) and HEAx (d). Model compound spectra were measured after dissolution in acetonitrile. FEAx-COF spectra were measured as powder. Asterisk indicates lamp change during solid-state UV-Vis measurement.

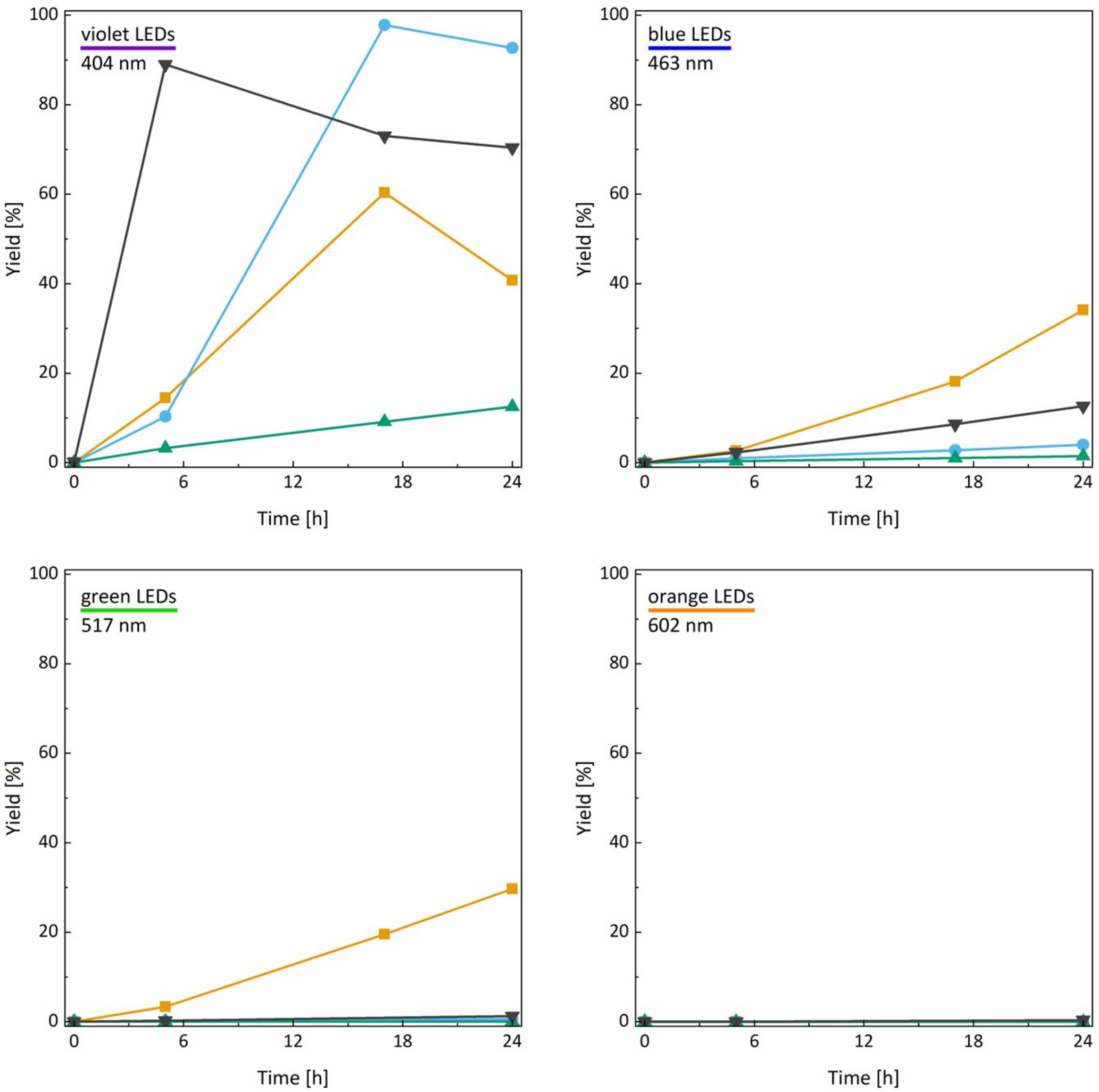

Figure S 21: Wavelength-dependent photocatalysis with FEAx COF (orange) and model compounds FEAx (blue), PEAx (green), and HEAx (grey). 20 mM MBA in MeCN/water (1:1, 1 mL), 2 mM molecular alloxazine or 1.5 mg FEAx-COF, O_2., room temperature. Yield determined *via* HPLC. Reactions under violet light show yields decreasing with progressing reaction time due to overoxidation of MBAld to MBAacid. See Figure S 19 for LED emission spectra.

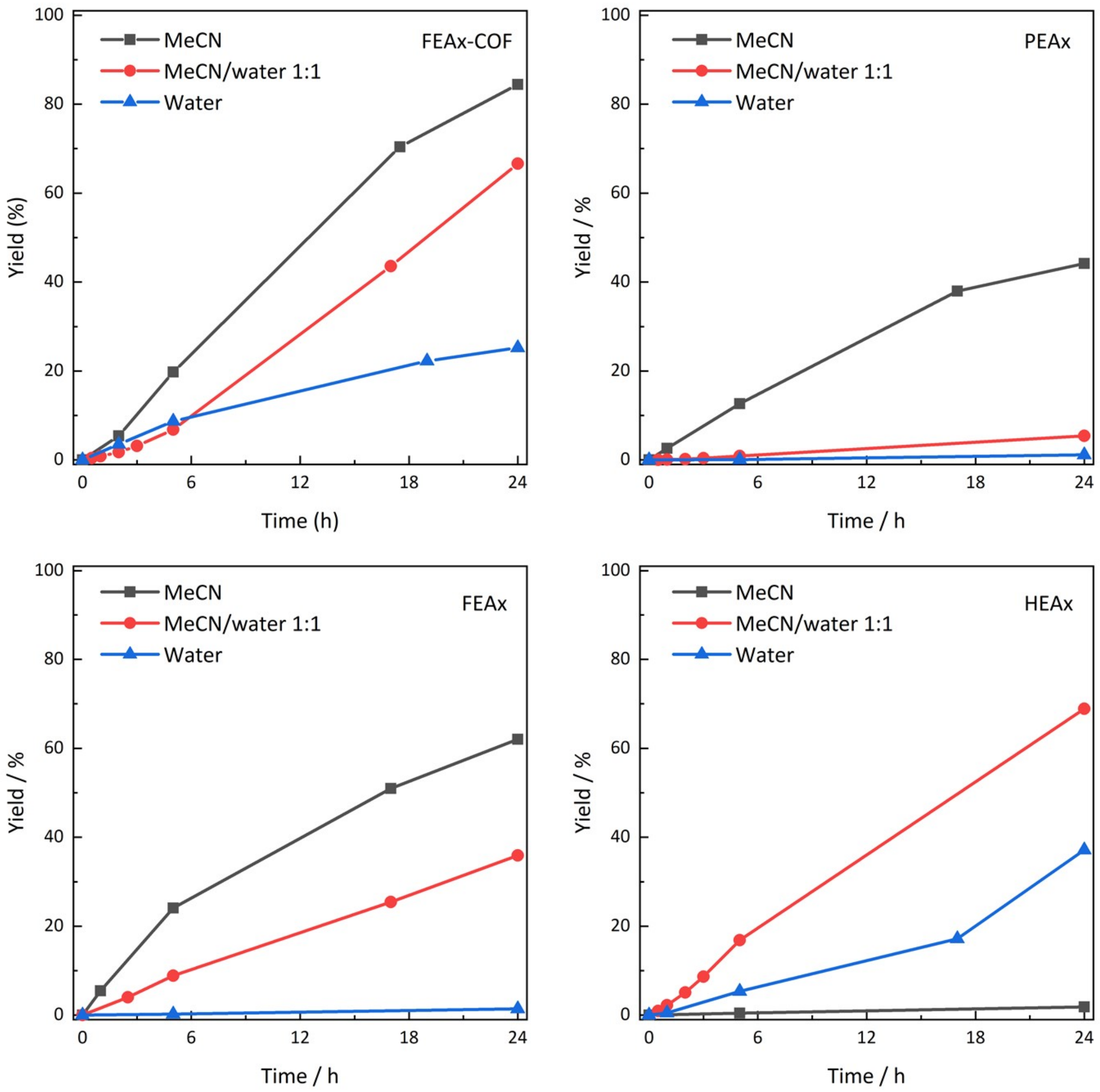

Figure S 22: Comparison of photocatalytic yields of FEAx COF and the model compounds FEAx, PEAx, and HEAx in various solvents. Reaction conditions: 20 mM MBA in 1 mL solvent, 2 mM molecular alloxazine or 1.5 mg FEAx-COF, O_2, 463 nm LEDs, 45 °C.

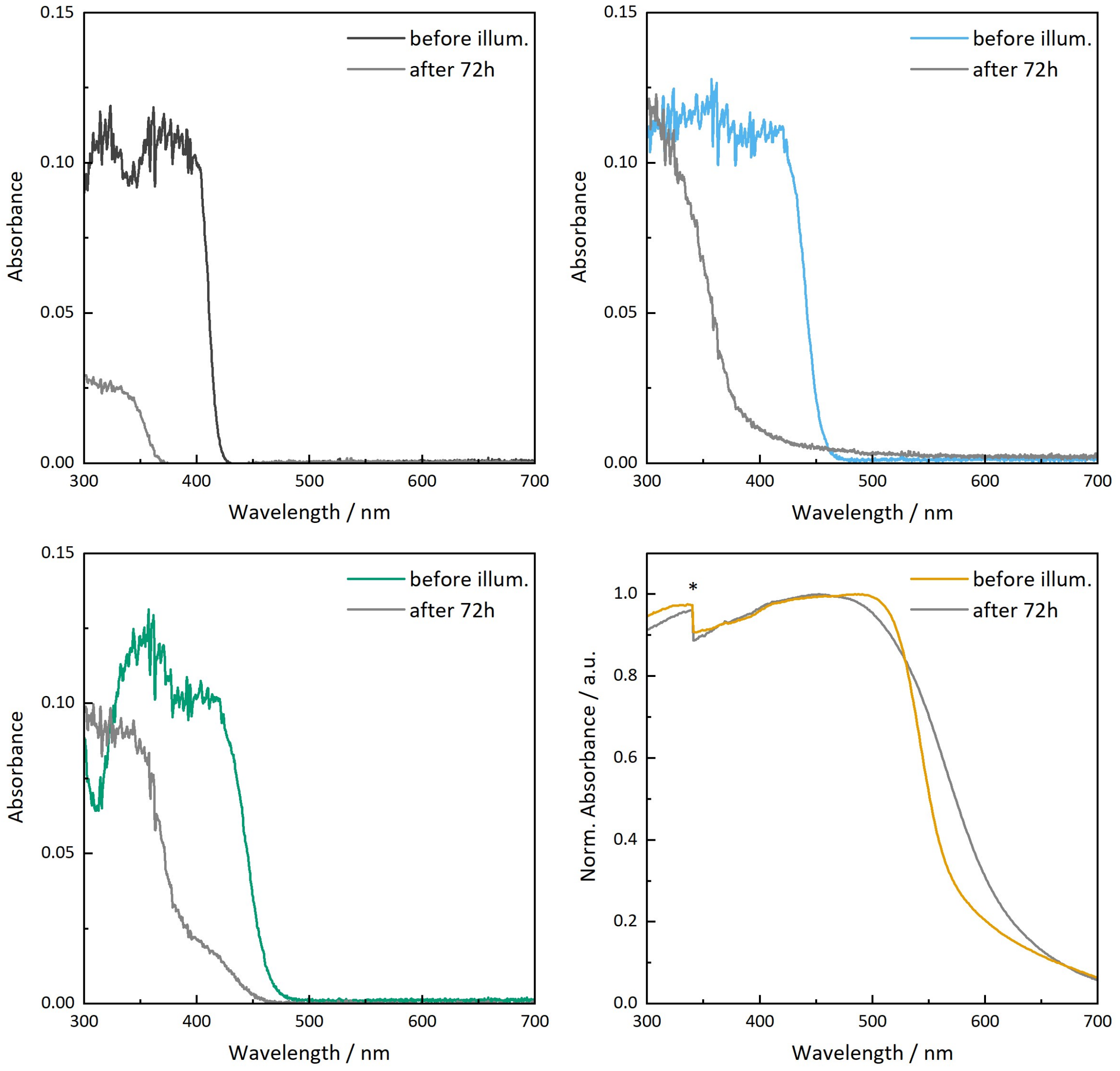

Figure S 23: UV-Vis spectra of HEAx (dark grey), PEAx (green), FEAx (blue), and FEAx-COF (orange) before and after illumination with violet LEDs for 72 h. Reaction conditions: 2 mM molecular alloxazine or 1.5 mg FEAx-COF in MeCN (1 mL), O_2, RT. Spectra of molecular alloxazines measured as 0.2 mM solutions (in MeCN) on a Varian Cary 50 spectrophotometer, FEAx-COF measured after isolation as a solid. Asterisk indicates lamp change during solid state UV-Vis measurement. Note that the data for FEAx-COF is normalized to account for potentially varying amounts of probed powder.

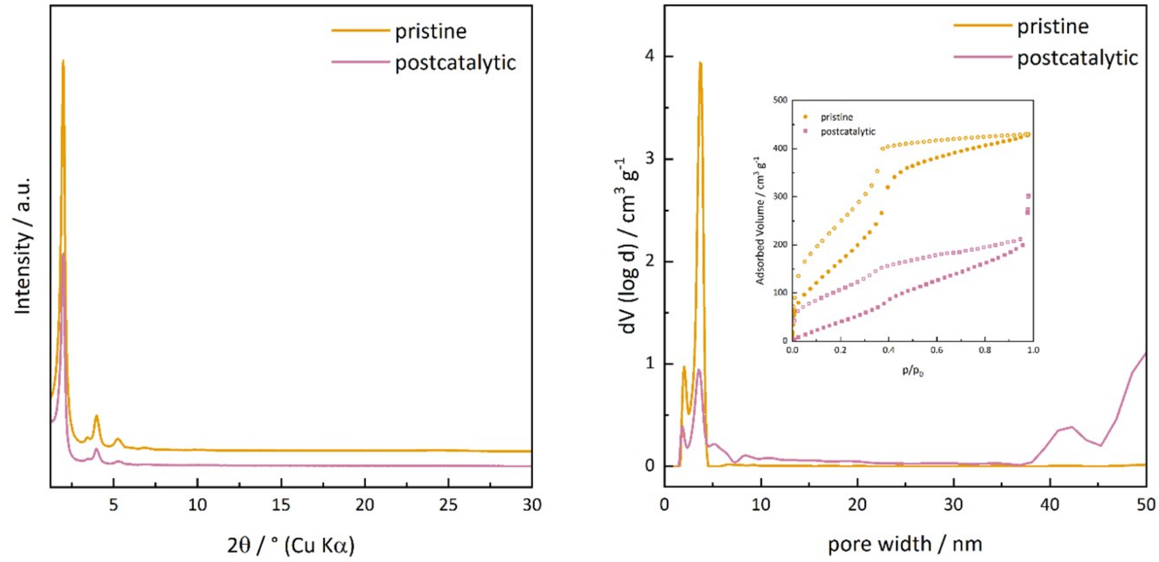

Figure S 24: Post-catalytic characterization of FEAx-COF *via* XRPD (left) and pore size distribution (right) after reaction in MeCN/water with 463 nm irradiation for 24 h. Inset shows underlying sorption isotherms. The BET surface area of this batch of FEAx-COF before and after photocatalysis was determined to be 677.4 and 238.5 $m^2\,g^{-1}$, respectively.

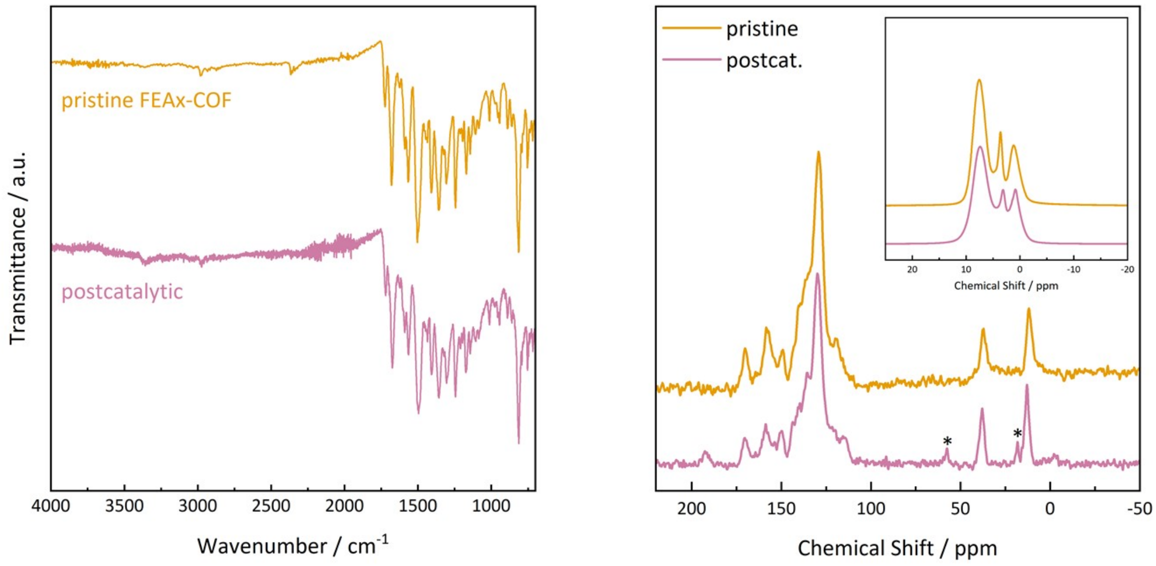

Figure S 25: Post-catalytic characterization of FEAx-COF *via* FTIR (left) and ^{13}C ssNMR (right). Inset shows ^{1}H ssNMR spectra. Asterisks indicate signals of residual ethanol from the supercritical drying procedure.

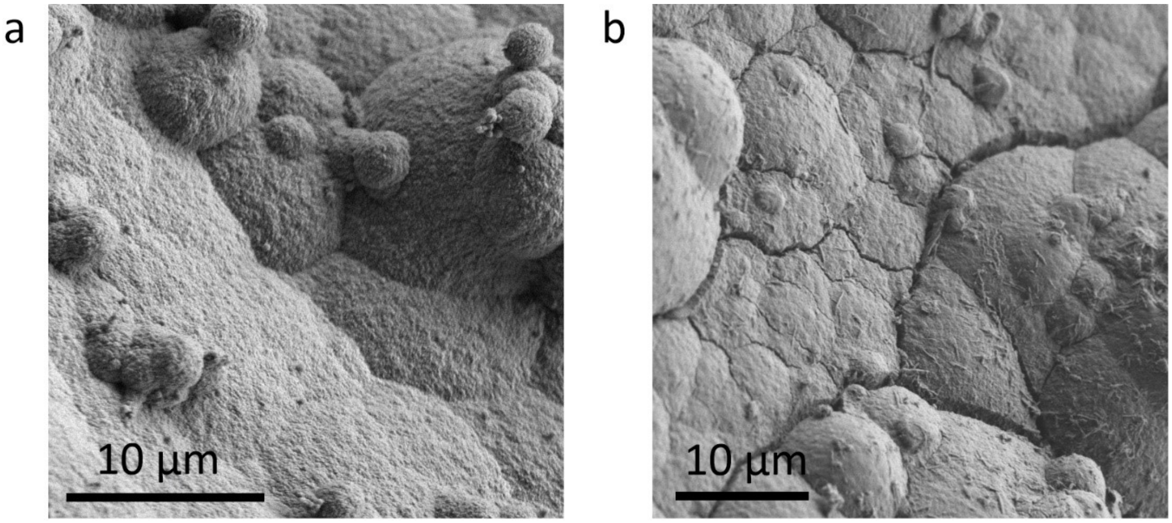

Figure S 26: SEM images of FEAx before (a) and after (b) photocatalysis.

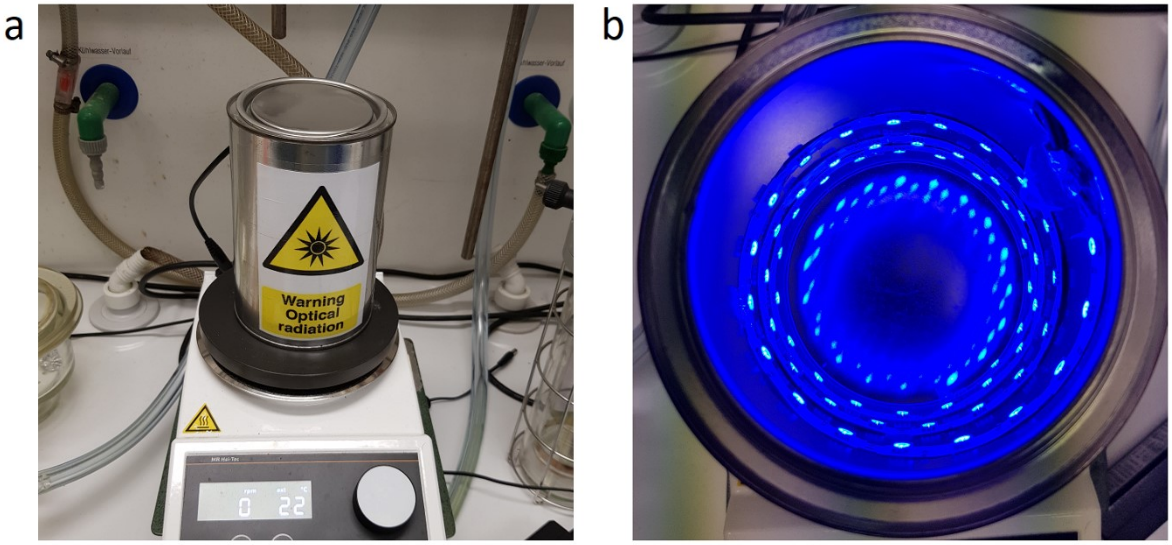

Figure S 27: Photograph of a photoreactor used for the photooxidation reactions (a). Top view into the opened reactor with 463 nm LEDs (b).

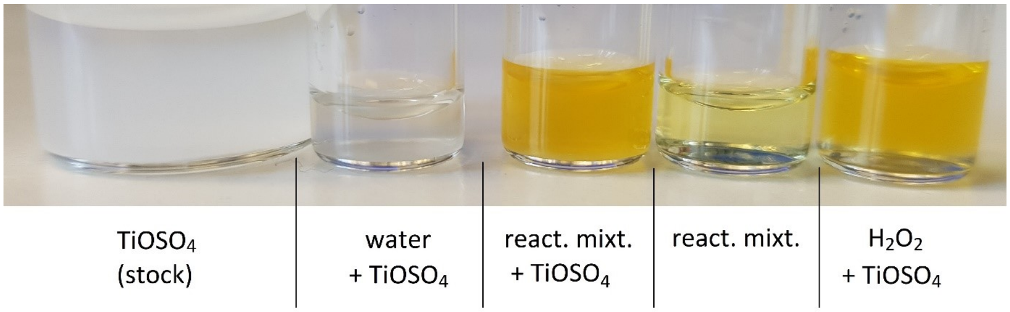

Figure S 28: Photograph of peroxide detection experiments with titanyl sulfate.

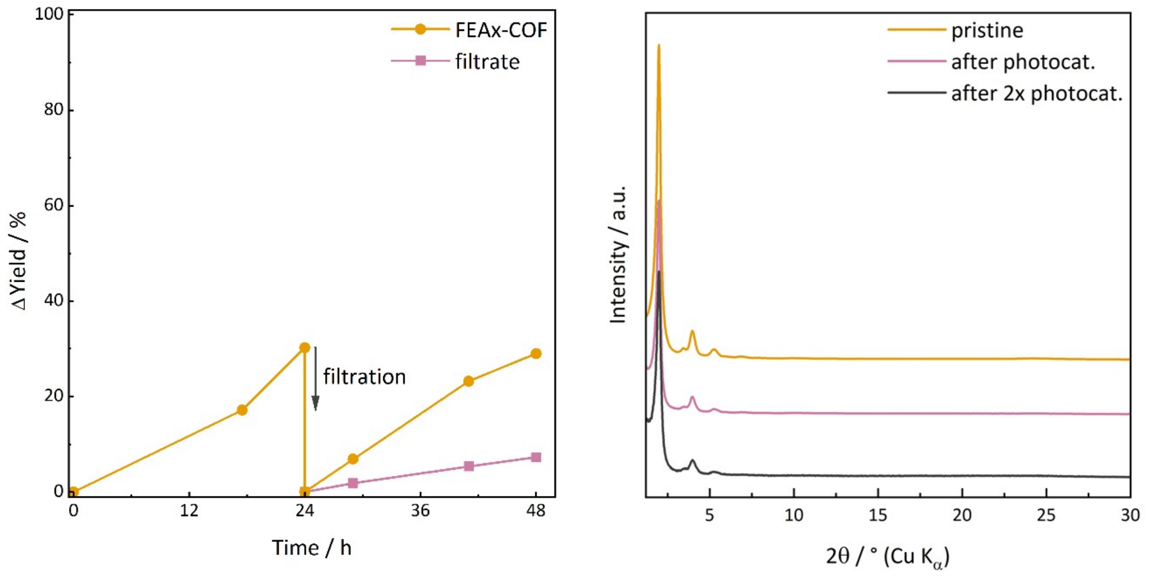

Figure S 29: Left: Filtration and recycling photocatalysis experiments with FEAx-COF. Reaction conditions: 20 mM MBA in MeCN/water (1:1, 1 mL), 1.5 mg FEAx-COF, O_2, 463 nm LEDs, RT. After 24 h, the COF was separated by centrifugation, washed, and reused for photocatalysis under identical conditions. The selectivity was 98.4% and 98.8% in the first and second run, respectively. The filtrate was illuminated under the same conditions, showing only little residual activity which we assign to residual nanoparticular COF that could not be centrifuged off. For better visualization, the yield for the filtrate is corrected by the amount of product already formed before filtration. Right: XRPD patterns for FEAx-COF before and after consecutive photocatalysis experiments.

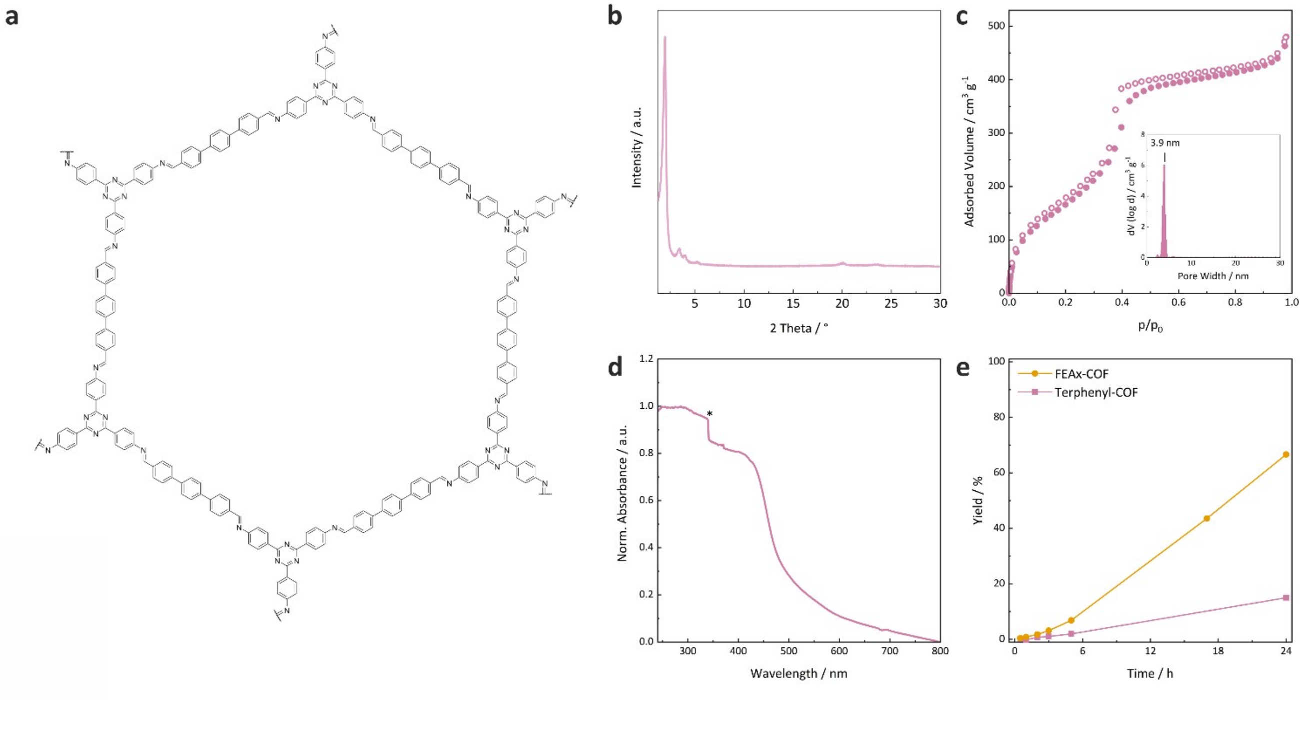

Figure S 30: (a) Molecular structure of Terphenyl-COF. (b) XRPD pattern of Terphenyl-COF. (c) Argon sorption isotherm of Terphenyl-COF at 87 K. Filled and open symbols represent the adsorption and the desorption branches, respectively. The inset shows the pore size distribution obtained from a QSDFT kernel for cylindrical pores. The BET surface area was determined to be 645.9 m^2 g^{-1}. (d) Solid-state UV-Vis spectrum of Terphenyl-COF. Asterisk indicates lamp change. (e) Photocatalytic efficacy of Terphenyl-COF compared to FEAx-COF in the oxidation of MBA. Reaction conditions: 20 mM MBA in MeCN/water (1:1, 1 mL), 1.5 mg COF, 463 nm LEDs, O_2. Yield determined via HPLC.

Table S 3: Photocatalytic oxidation of benzylic alcohols and other substrates by FEAx-COF.

Entry	Substrate	Product	Yield / %	E_{ox} / V [a]	Ref.
	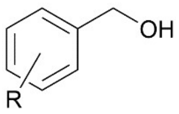	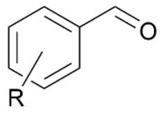			
1	R = 4-NO_2		traces	2.84	[24]
2	R = 4-tBu		3	2.06	[25]
3	R = 4-H		0	1.94	[24]
4	R = 4-Me		2	1.84	[24]
5	R = 4-OMe		17[b]	1.48[c]	[26]
6	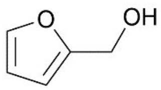	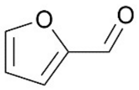	0	1.73	[27]
7	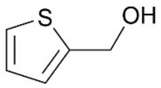	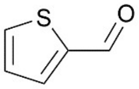	16	0.72[d]	[28]
8	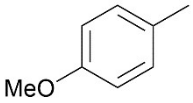	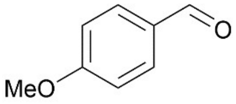	15[e]	1.35-1.42	[29]
9	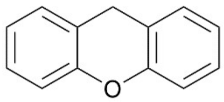	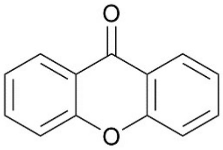	14	1.51	[30]
10	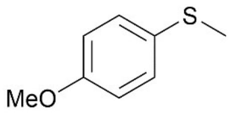	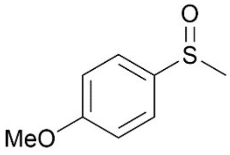	12	1.13	[30]

Reaction conditions: 250 mM substrate, 1.5 mg FEAx-COF, 463 nm LEDs, water/MeCN (1 mL, 1:1), 45 °C, O_2. Yield after 24 h determined *via* NMR with internal standard [a] *vs*. SCE. [b] Yield after 24 h determined *via* HPLC. [c] 1.52 V *vs*. Ag/AgCl. [d] 0.76 V *vs*. Ag/AgCl. [e] Side product MBAcid formed.

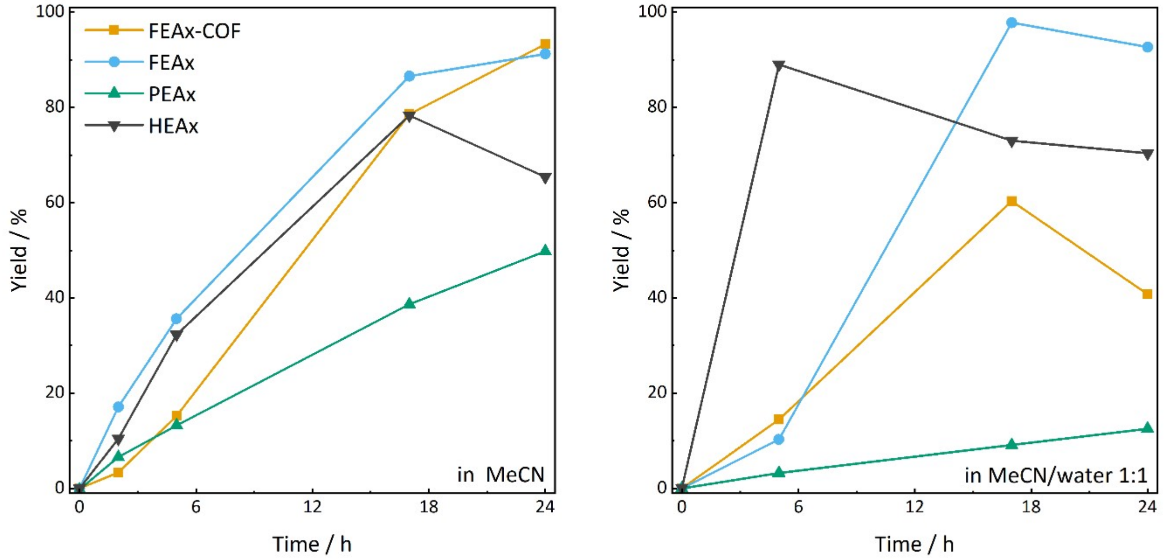

Figure S 31: Comparison of photocatalytic yields of FEAx COF and the model compounds FEAx, PEAx, and HEAx in acetonitrile (left) and acetonitrile /water 1:1 (right). Reaction conditions: 20 mM MBA in 1 mL solvent, 2 mM molecular alloxazine or 1.5 mg FEAx-COF, O_2, 404 nm LEDs, rt. Decreasing yields with progressing reaction time are due to overoxidation of MBAld to MBAacid.

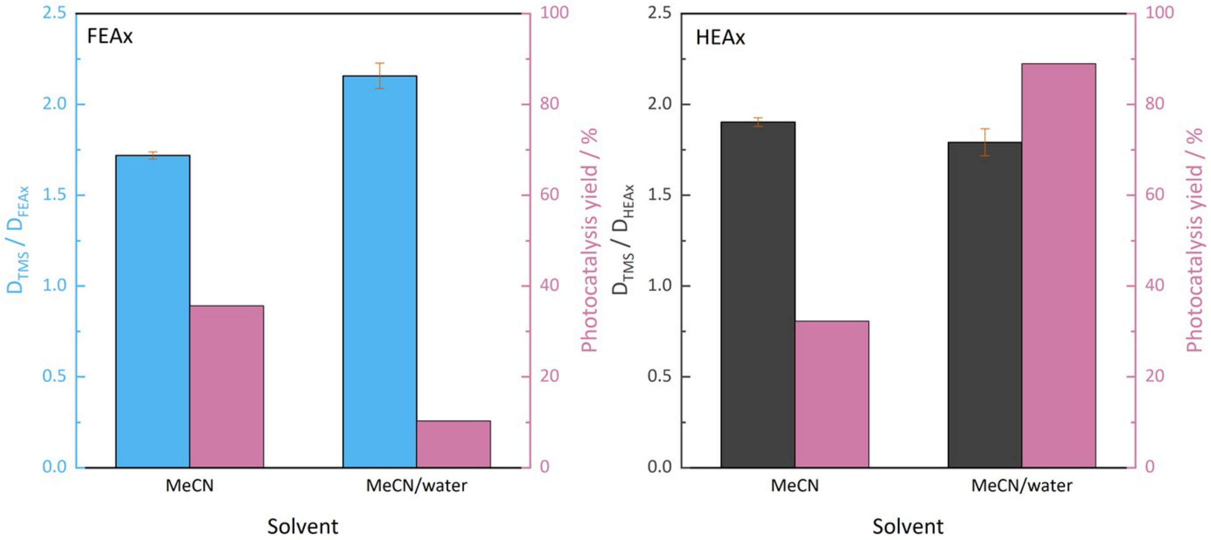

Figure S 32: Relative diffusion coefficients and photocatalytic activity in different solvents (c = 2 mM) for FEAX (left) and HEAX (right). Photocatalysis reaction conditions: 20 mM MBA (1 mL solvent), 1.5 mg COF, 404 nm LEDs, O_2, rt. Yield determined *via* HPLC after 5 h, since longer irradiation with 404 nm LEDs in MeCN/water leads to overoxidation (see Figure S21). Error bars represent the standard deviation of at least three measurements.

The relative diffusion coefficient is proportional to the hydrodynamic radius of the diffusing species. An increase in D_{TMS}/D_M between the different solvents is thus caused by an increase in aggregate size. While HEAX deaggregates in MeCN/water 1:1, following the reported trends for similar flavins,[31] FEAX shows an opposite trend with more pronounced aggregation in the aqueous solvent mixture. It can be seen that the photocatalytic activity is higher for the respective less aggregated species.

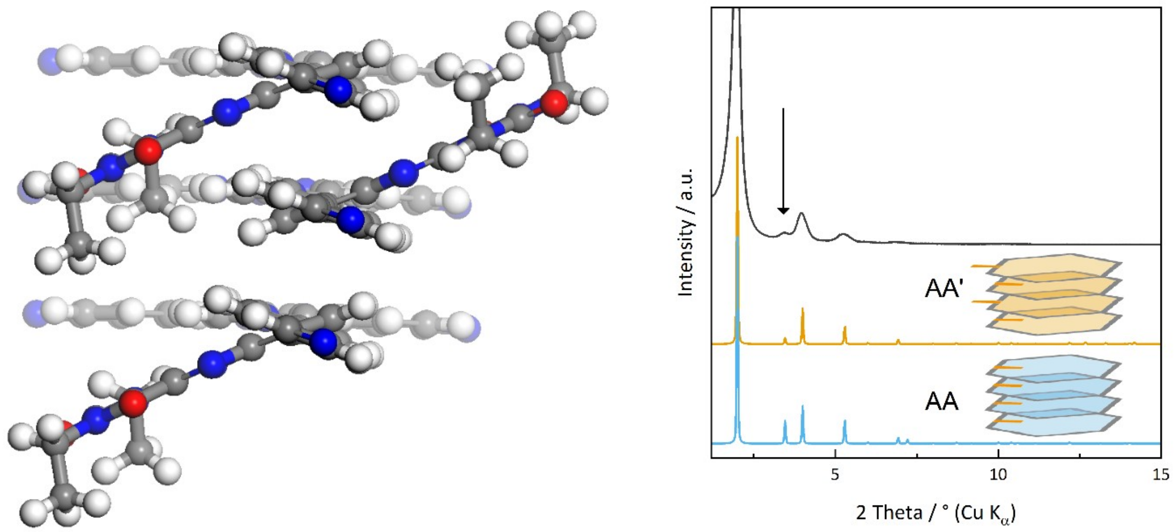

Figure S 33: Schematic representation of alloxazine orientation in FEAx-COF (left) and simulated XRPD patterns for FEAx-COF with different alloxazine stacking modes compared to experimental data (right). Alloxazine orientation visualized as orange lines.

The structural model for FEAx-COF that best fits the experimental XRPD data consists of COF layers with alternating orientation of the alloxazine core (AA', orange model, Figure S32). When all alloxazines are stacked on top of each other (AA, blue model), the simulated pattern shows varying intensities especially at 3.93° (200), which is not represented in the experimental data.

Quantum Chemical Calculations

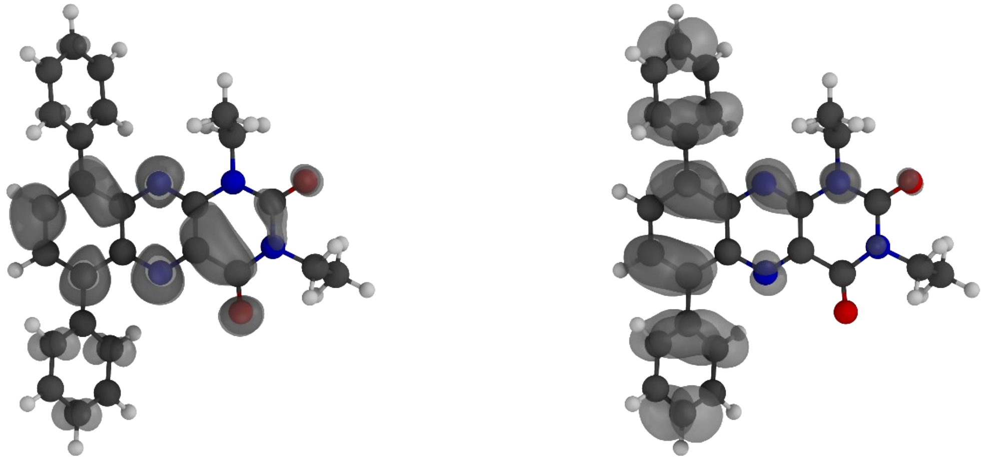

Figure S 34: Calculated spin densities for the PEAx model compound, radical anion (left) and radical cation (right).

Table S 4: Calculated vertical radical stabilization energies, obtained as total energy differences between radical anionic (Vertical Radical Anion Stabilization Energy, VRASE), radical cationic (Vertical Radical Cation Stabilization Energy, VRCSE), and neutral states.

		Radical Cation	Neutral	Radical Anion		
VRCSE [kcal/mol]	VRCSE [H]	Total Energy [H]	Total Energy [H]	Total Energy [H]	VRASE [H]	VRASE [kcal/mol]
173.58	0.276622	-1372.213663	-1372.490285	-1372.545593	-0.055308	-34.71

Table S 5: Calculated Total Energies and Reaction Enthalpies as corresponding differences for investigated model systems on PBE0-D3/def2-TZVP level of theory. Solvation effects have been considered using the implicit solvation model COSMO with a value of 36.64 as the dielectric constant to represent acetonitrile.[32]

	Gas Phase	Acetonitrile	Delta Total Energy		
	Total Energy [H]	Total Energy [H]	[H]	[kcal/mol]	[kJ/mol]
PEAx	-1372.490285	-1372.512292	-0.022007	-13.81	-57.78
MBA	-460.947382	-460.961837	-0.014455	-9.07	-37.95
	Reaction Enthalpy (Gas Phase)		0.217118	136.24	570.04
	Reaction Enthalpy (Acetonitrile)		0.199992	125.50	525.08
PEAx-RA	-1372.553223	-1372.629172	-0.075949	-47.66	-199.40
MBA-RK	-460.667326	-460.644965	0.022361	14.03	58.71
	Reaction Enthalpy (Gas Phase)		-0.128895	-80.88	-338.41
	Reaction Enthalpy (Acetonitrile)		-0.114866	-72.08	-301.58
PEAx-H-R	-1373.069373	-1373.097628	-0.028255	-17.73	-74.18
MBA-R	-460.280071	-460.291375	-0.011303	-7.09	-29.68

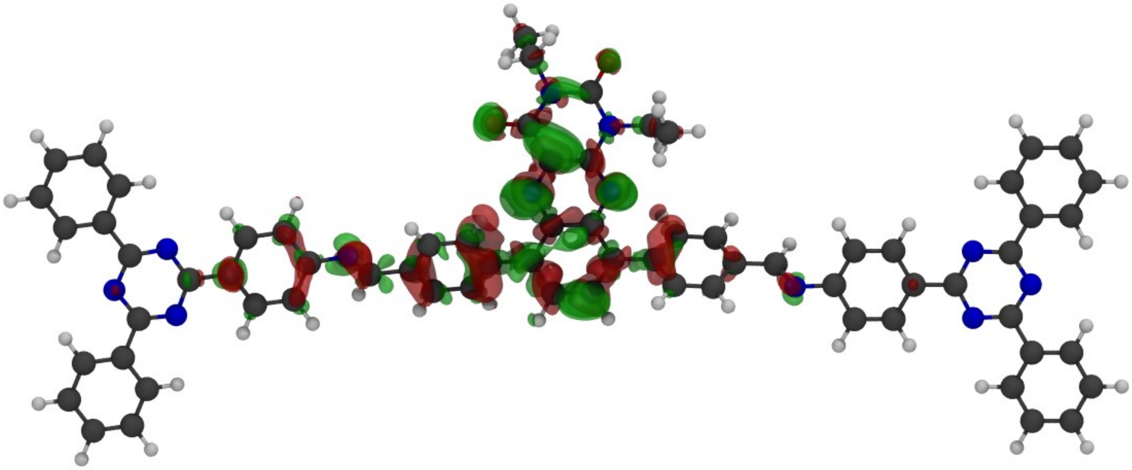

Figure S 35: Calculated difference density for the lowest vertical excitation for the FEAx-COF pore edge model, obtained on the TD-PBE0-D3/def2-TZVP level of theory. Red isosurfaces depict regions with lower electron density in the excited state whereas green isosurfaces represent higher electron density in the excited state, both in comparison to the electron density of the ground state. The extent of the isosurfaces shows a coverage along the COF pore edge and visualize the conjugation of the alloxazine cores in FEAx-COF.

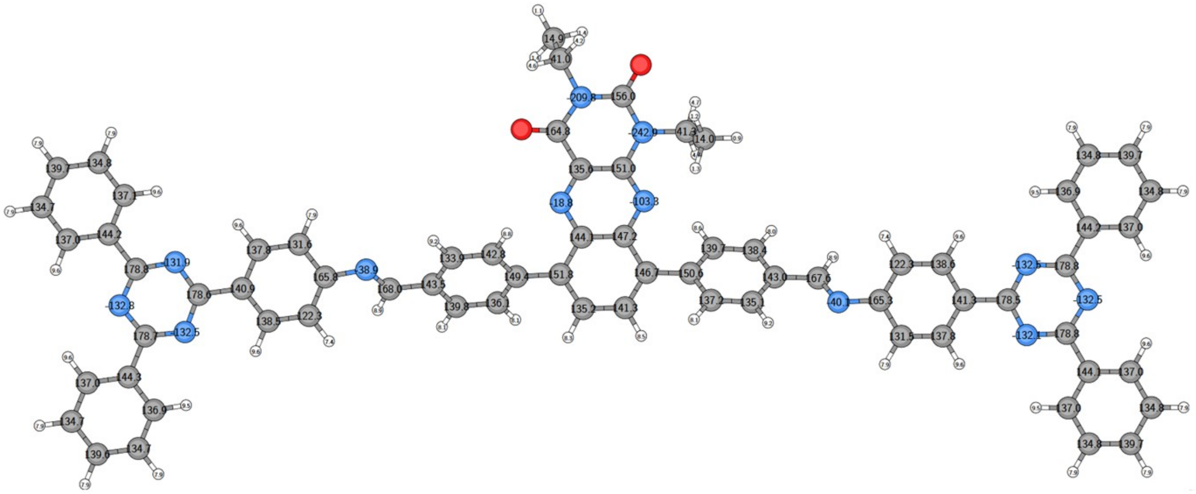

Figure S 36: Calculated NMR chemical shifts for the FEAx-COF pore edge model, obtained at the B97-2/pcsSeg-2//PBE0-D3/def2-TZVP level of theory.

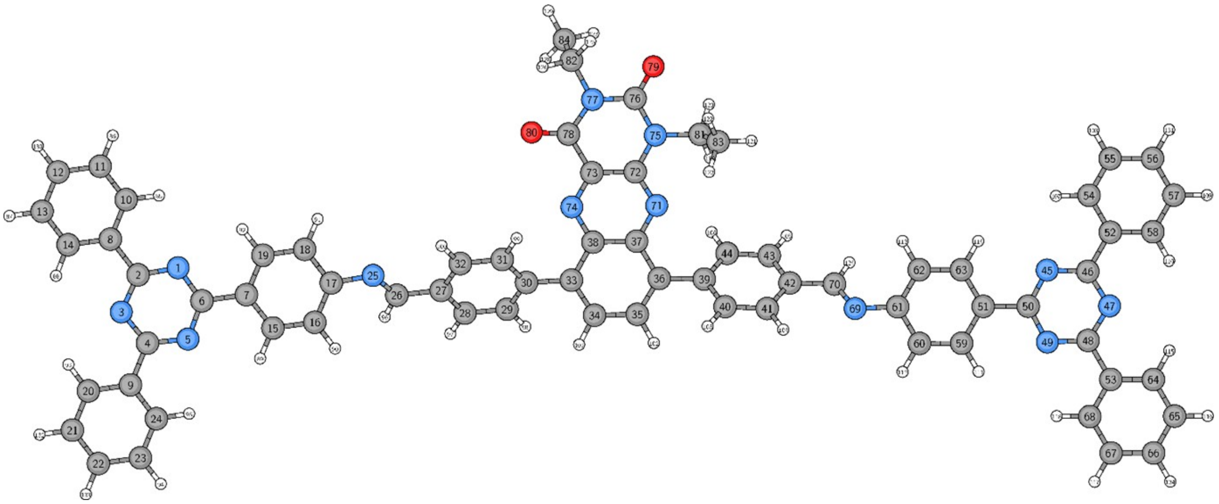

Figure S 37: Atom labels for the FEAx-COF pore edge model.

Table S 6: Calculated NMR chemical shifts for the FEAx-COF pore edge model, obtained at the B97-2/pcsSeg-2//PBE0-D3/def2-TZVP level of theory.

Number	Atom	NMR Chemical Shielding [ppm]	NMR Chemical Shift [ppm]
1	N	-11.35	-131.9
2	C	6.51	178.8
3	N	-10.44	-132.8
4	C	6.62	178.7
5	N	-10.75	-132.5
6	C	6.70	178.6
7	C	44.41	140.9
8	C	41.11	144.2
9	C	41.00	144.3
10	C	48.23	137.1
11	C	50.50	134.8
12	C	45.65	139.7
13	C	50.63	134.7
14	C	48.36	137.0
15	C	46.77	138.5
16	C	62.98	122.3
17	C	19.55	165.8
18	C	53.70	131.6
19	C	47.52	137.8
20	C	48.30	137.0
21	C	50.58	134.7
22	C	45.74	139.6
23	C	50.61	134.7
24	C	48.40	136.9
25	N	-104.32	-38.9
26	C	17.29	168.0
27	C	41.79	143.5
28	C	45.55	139.8
29	C	49.18	136.1

Number	Atom	NMR Chemical Shielding [ppm]	NMR Chemical Shift [ppm]
30	C	35.88	149.4
31	C	42.47	142.8
32	C	51.36	133.9
33	C	33.54	151.8
34	C	50.12	135.2
35	C	44.05	141.3
36	C	38.65	146.7
37	C	38.10	147.2
38	C	41.17	144.1
39	C	34.75	150.6
40	C	48.10	137.2
41	C	50.19	135.1
42	C	42.27	143.0
43	C	46.88	138.4
44	C	45.62	139.7
45	N	-10.77	-132.5
46	C	6.54	178.8
47	N	-10.75	-132.5
48	C	6.47	178.8
49	N	-11.20	-132.1
50	C	6.85	178.5
51	C	44.04	141.3
52	C	41.13	144.2
53	C	41.18	144.1
54	C	48.42	136.9
55	C	50.56	134.8
56	C	45.60	139.7
57	C	50.51	134.8
58	C	48.30	137.0
59	C	47.53	137.8
60	C	53.82	131.5
61	C	19.97	165.3
62	C	62.99	122.3
63	C	46.70	138.6
64	C	48.32	137.0
65	C	50.55	134.8
66	C	45.58	139.7
67	C	50.51	134.8
68	C	48.31	137.0
69	N	-103.11	-40.1
70	C	17.72	167.6
71	N	-39.98	-103.3
72	C	34.33	151.0
73	C	49.72	135.6
74	N	-124.46	-18.8
75	N	99.64	-242.9

Number	Atom	NMR Chemical Shielding [ppm]	NMR Chemical Shift [ppm]
76	C	29.35	156.0
77	N	66.57	-209.8
78	C	20.47	164.8
81	C	144.02	41.3
82	C	144.31	41.0
83	C	171.34	14.0
84	C	170.41	14.9
85	H	22.07	9.6
86	H	23.73	7.9
87	H	23.78	7.9
88	H	22.08	9.6
89	H	22.02	9.6
90	H	24.21	7.4
91	H	23.72	7.9
92	H	22.02	9.6
93	H	22.07	9.6
94	H	23.74	7.9
95	H	22.08	9.5
96	H	22.75	8.9
97	H	23.58	8.1
98	H	23.56	8.1
99	H	22.80	8.8
100	H	22.43	9.2
101	H	23.30	8.3
102	H	23.17	8.5
103	H	23.58	8.1
104	H	22.44	9.2
105	H	23.59	8.0
106	H	23.06	8.6
107	H	22.09	9.5
108	H	23.73	7.9
109	H	23.77	7.9
110	H	22.07	9.6
111	H	22.02	9.6
112	H	23.77	7.9
113	H	24.20	7.4
114	H	22.01	9.6
115	H	22.08	9.6
116	H	23.77	7.9
117	H	23.73	7.9
118	H	22.08	9.5
119	H	26.94	4.7
120	H	26.96	4.7
121	H	30.74	0.9
122	H	30.46	1.2
123	H	30.33	1.3

Number	Atom	NMR Chemical Shielding [ppm]	NMR Chemical Shift [ppm]
124	H	22.73	8.9
125	H	27.42	4.2
126	H	27.08	4.6
127	H	30.20	1.4
128	H	30.22	1.4
129	H	30.52	1.1
130	H	23.74	7.9
131	H	23.73	7.9
132	H	23.78	7.9
133	H	23.74	7.9
134	H	23.74	7.9

Crystal Structures

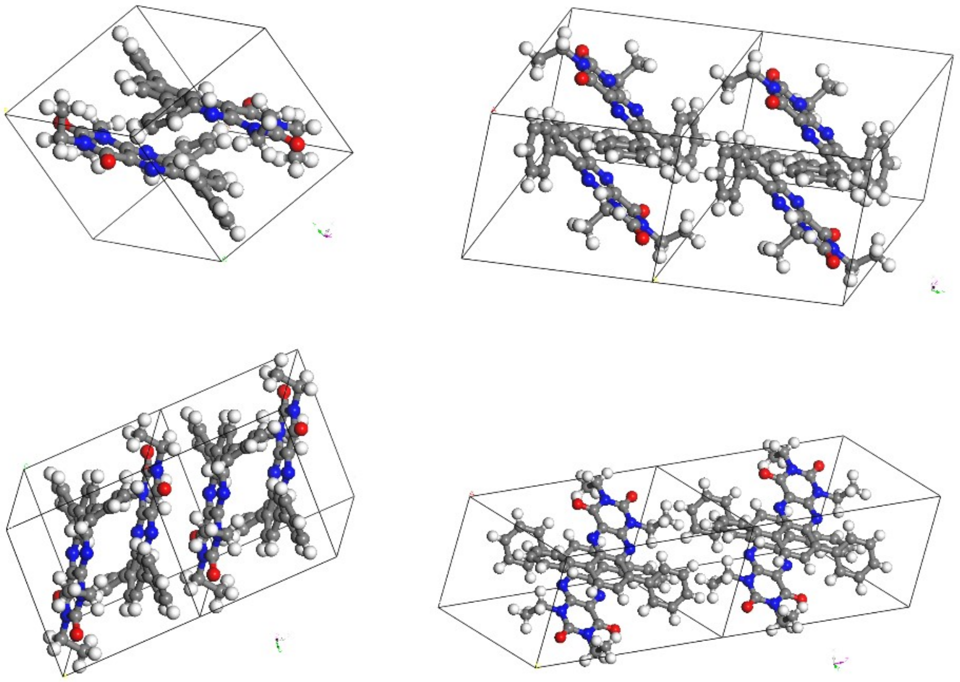

Figure S 38: Crystal structure of PEAx.

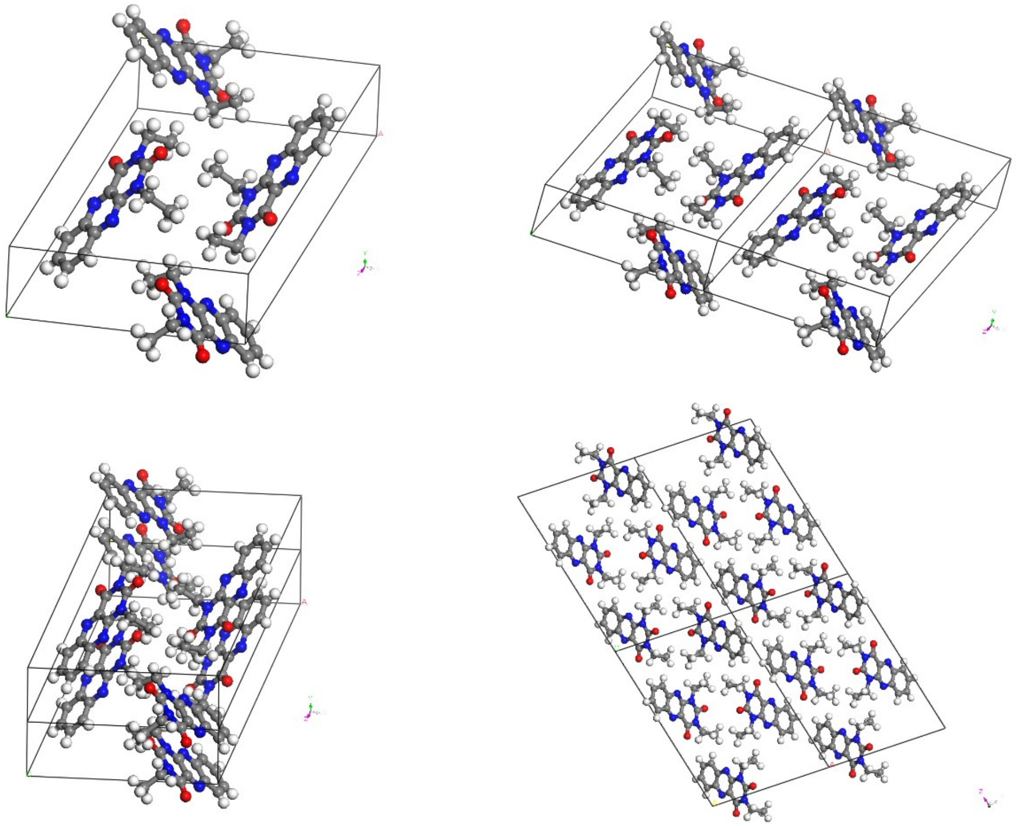

Figure S 39: Crystal structure of HEAx.

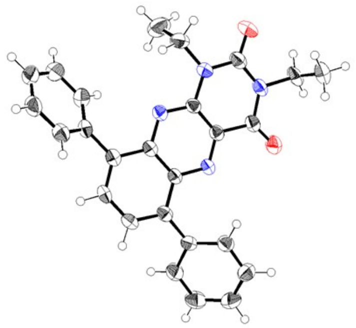

Figure S 40: Molecular structure of PEAx with thermal ellipsoids (50% probability).

Table S 7: Crystallographic data and structure refinement for PEAx.

Identification code	STB226_P-1
Empirical formula	$C_{26}H_{22}N_4O_2$
Formula weight	422.47
Crystal Size/mm	0.030 x 0.005 x 0.005
Crystal color	Yellow
Temperature/K	298.7
Crystal system	triclinic
Space group	P-1
a/Å	8.4529(6)
b/Å	10.7933(6)
c/Å	13.3520(8)
α/°	67.316(2)
β/°	71.863(3)
γ/°	87.153(3)
Volume/Å^3	1064.65(12)
Z	2
ρ_{calc}g/cm^3	1.318
μ/mm^{-1}	0.086
F(000)	444.0
Radiation	MoKα (λ = 0.71073)
2Θ range for data collection/°	5.088 to 55
Index ranges	-10 ≤ h ≤ 10, -14 ≤ k ≤ 13, -17 ≤ l ≤ 17
Reflections collected	13350
Independent reflections	4827 [R_{int} = 0.0539, R_{sigma} = 0.0712]
Data/restraints/parameters	4827/0/291
Goodness-of-fit on F^2	1.069
Final R indexes [I>=2σ (I)]	R_1 = 0.0702, wR_2 = 0.1419
Final R indexes [all data]	R_1 = 0.1381, wR_2 = 0.1691
Largest diff. peak/hole / e Å^{-3}	0.21/-0.21

Table S 8: Fractional Atomic Coordinates (×10[4]) and Equivalent Isotropic Displacement Parameters (Å[2]×10[3]) for PEAx. U_{eq} is defined as 1/3 of the trace of the orthogonalised U_{IJ} tensor.

Atom	x	y	z	U_{eq}
O001	9761(3)	2964(2)	4267.4(17)	52.7(6)
N002	6506(3)	5463(2)	6316.8(17)	33.6(5)
N003	7905(2)	5167(2)	4229.2(17)	32.7(5)
N004	9237(3)	2286(2)	6180.6(19)	40.1(6)
O005	8613(3)	1536(2)	8109.2(18)	65.6(7)
N006	7545(3)	3468(2)	7232.0(18)	39.3(6)
C007	6943(3)	6202(2)	4280(2)	31.4(6)
C008	7363(3)	4438(2)	6239(2)	33.3(6)
C009	6284(3)	6366(2)	5339(2)	31.8(6)
C00A	8111(3)	4312(2)	5183(2)	32.9(6)
C00B	5344(3)	7491(3)	5399(2)	34.4(6)
C00C	6622(3)	7120(2)	3266(2)	34.3(6)
C00D	4676(3)	7720(2)	6481(2)	37.1(6)
C00E	7274(3)	7004(3)	2140(2)	36.1(6)
C00F	7366(3)	5772(3)	2028(2)	40.4(7)
C00G	3040(3)	8066(3)	6806(2)	44.6(7)
C00H	5059(3)	8343(3)	4412(2)	39.2(7)
C00I	9117(3)	3158(3)	5132(2)	37.3(6)
C00J	5661(3)	8156(3)	3381(2)	41.5(7)
C00K	8472(4)	2383(3)	7235(3)	43.8(7)
C00L	2397(4)	8301(3)	7790(3)	52.1(8)
C00M	7884(4)	5716(3)	958(2)	48.3(7)
C00N	5632(4)	7616(3)	7181(2)	44.2(7)
C00O	10164(4)	1079(3)	6214(3)	47.2(7)
C00P	6788(4)	3613(3)	8337(2)	48.7(8)
C00Q	3360(4)	8203(3)	8471(3)	55.1(9)
C00R	8324(4)	6878(3)	-16(3)	56.9(9)
C00S	4983(4)	7859(3)	8166(3)	54.5(8)
C00T	7759(4)	8170(3)	1139(2)	51.9(8)
C00U	8996(4)	-102(3)	6484(3)	54.9(8)
C00V	8269(4)	8104(3)	76(3)	62.5(9)
C00W	7965(5)	4373(4)	8575(3)	74.2(11)

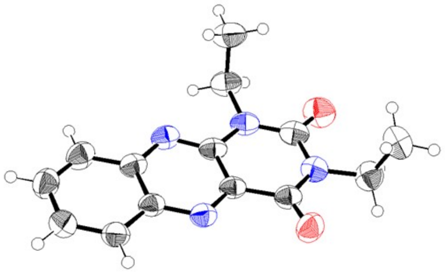

Figure S 41: Molecular structure of HEAx with thermal ellipsoids (50% probability).

Table S 9: Crystallographic data and structure refinement for HEAx.

Identification code	STB228-P2 1 c
Empirical formula	$C_{14}H_{14}N_4O_2$
Formula weight	270.29
Crystal Size/mm	0.300 x 0.060 x 0.040
Crystal color	Yellow
Temperature/K	298.6
Crystal system	monoclinic
Space group	$P2_1/c$
a/Å	13.3313(5)
b/Å	5.0391(2)
c/Å	19.8573(7)
α/°	90
β/°	103.665(2)
γ/°	90
Volume/Å^3	1296.21(8)
Z	4
ρ_{calc}g/cm^3	1.385
μ/mm^{-1}	0.097
F(000)	568.0
Radiation	MoKα (λ = 0.71073)
2Θ range for data collection/°	8.29 to 58.816
Index ranges	-18 ≤ h ≤ 18, -6 ≤ k ≤ 6, -27 ≤ l ≤ 27
Reflections collected	23975
Independent reflections	3523 [R_{int} = 0.0505, R_{sigma} = 0.0358]
Data/restraints/parameters	3523/0/183
Goodness-of-fit on F^2	1.070
Final R indexes [I>=2σ (I)]	R_1 = 0.0615, wR_2 = 0.1510
Final R indexes [all data]	R_1 = 0.0976, wR_2 = 0.1683
Largest diff. peak/hole / e Å^{-3}	0.20/-0.14

Table S 10: Fractional Atomic Coordinates (×10^4) and Equivalent Isotropic Displacement Parameters (Å^2×10^3) for HEAx. U_{eq} is defined as 1/3 of the trace of the orthogonalised U_{IJ} tensor.

Atom	x	y	z	U(eq)
O001	8664.7(11)	4816(3)	5798.0(7)	61.2(4)

Atom	x	y	z	U(eq)
O002	6242.7(13)	-925(3)	4641.0(9)	72.4(5)
N003	7473.9(12)	5255(3)	3343.9(8)	48.3(4)
N004	8858.5(11)	7063(3)	4562.8(8)	44.6(4)
N005	7483.7(12)	1886(3)	5219.1(9)	49.8(4)
N006	6831.4(12)	2164(3)	4002.0(9)	49.4(4)
C007	8160.0(14)	3991(4)	5248.0(10)	46.6(4)
C008	8199.6(13)	5134(4)	4570.8(9)	42.2(4)
C009	8171.8(14)	7202(4)	3322.8(9)	46.0(4)
C00A	9566.4(14)	10195(4)	3896.1(10)	50.5(5)
C00B	8869.4(13)	8121(4)	3936.7(9)	43.3(4)
C00C	7503.1(13)	4221(4)	3954.1(9)	43.8(4)
C00D	9571.0(16)	11289(5)	3269.9(11)	55.3(5)
C00E	6813.5(15)	922(4)	4615.3(11)	52.6(5)
C00F	6628(2)	2214(5)	6187.6(13)	71.6(7)
C00G	6051.3(16)	1346(4)	3379.4(11)	57.5(5)
C00H	8884.7(17)	10374(5)	2661.0(11)	59.6(5)
C00I	8204.9(17)	8395(5)	2686.8(10)	57.8(5)
C00J	7392.3(18)	718(4)	5881.1(12)	60.4(5)
C00K	5071.7(17)	2913(5)	3305.5(14)	71.6(7)

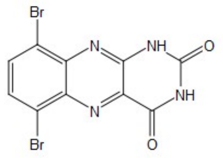

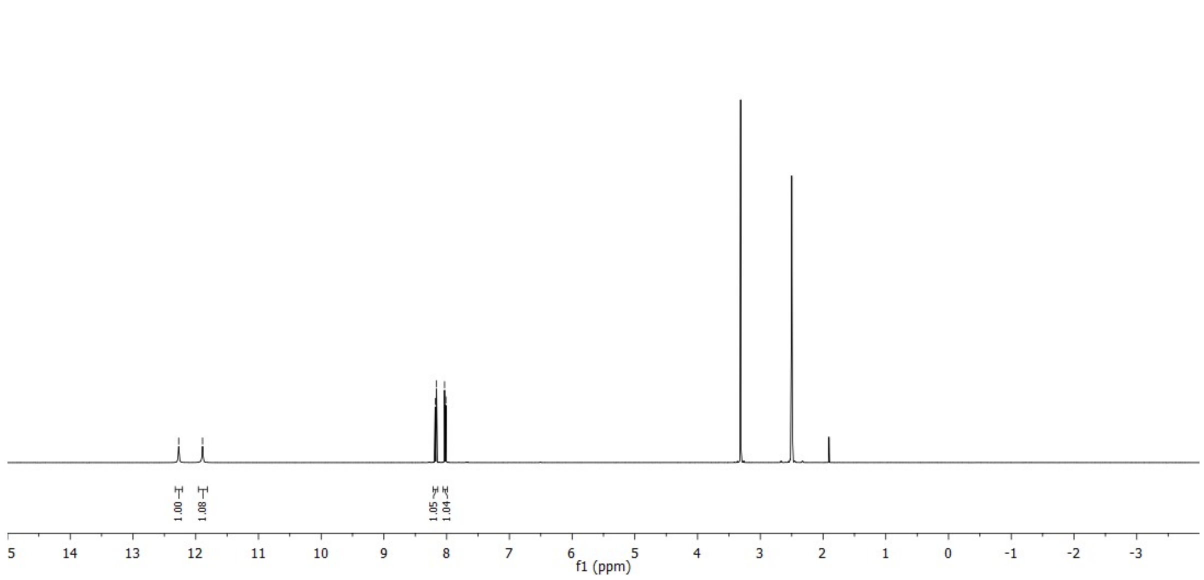

Figure S 42: ^{1}H-NMR spectrum of 6,9-dibromoalloxazine **S2** (DMSO-d_6, 400 MHz).

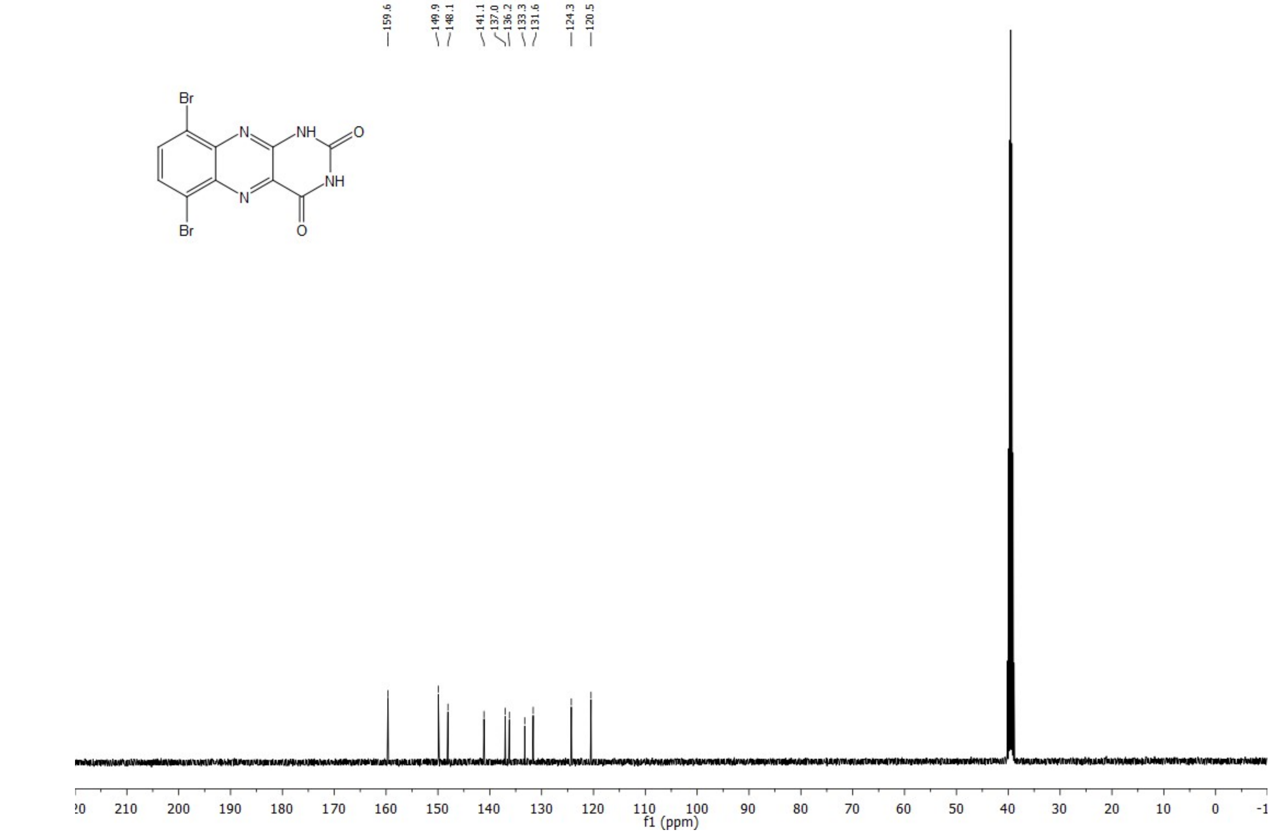

Figure S 43: ^{13}C-NMR spectrum of 6,9-dibromoalloxazine **S2** (DMSO-d_6, 101 MHz).

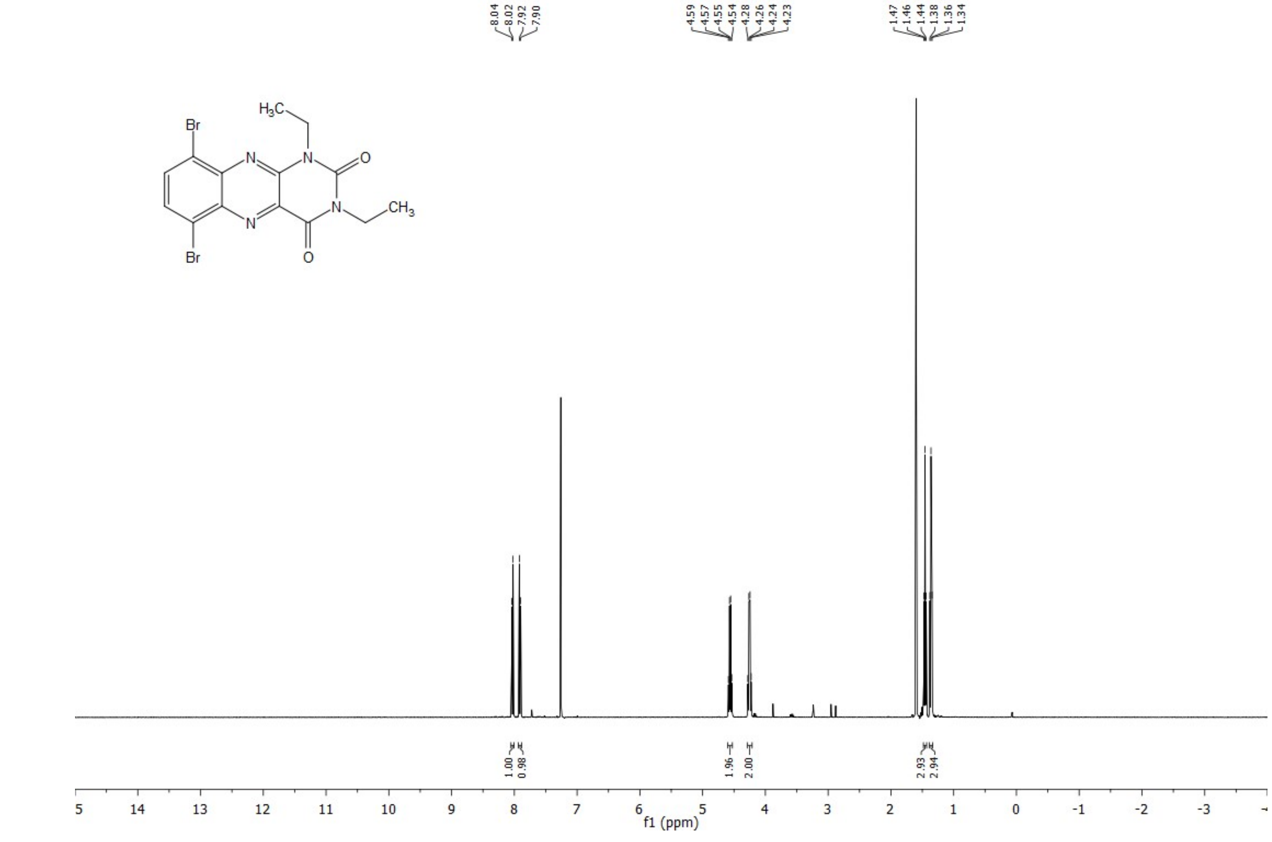

Figure S 44: ^{1}H-NMR spectrum of 1,3-diethyl-6,9-dibromoalloxazine **S3** ($CDCl_3$, 400 MHz).

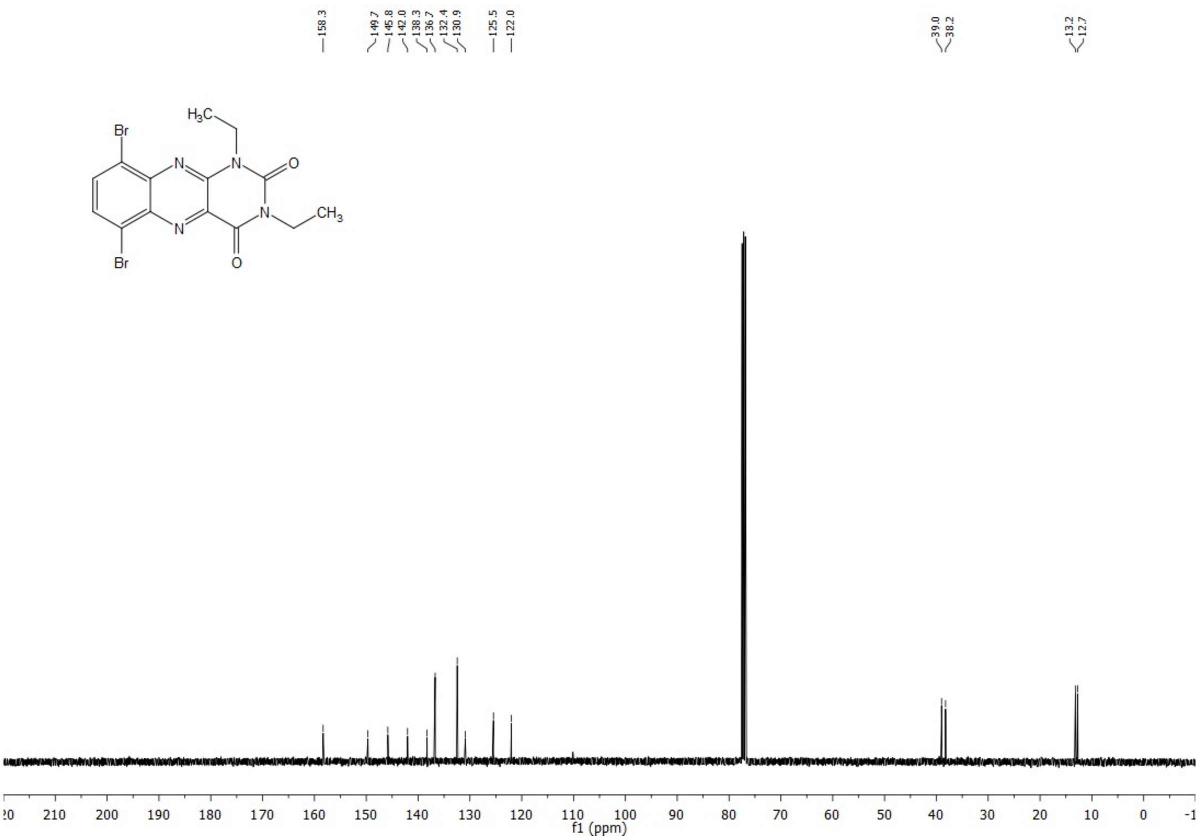

Figure S 45: ^{13}C-NMR spectrum of 1,3-diethyl-6,9-dibromoalloxazine **S3** ($CDCl_3$, 101 MHz).

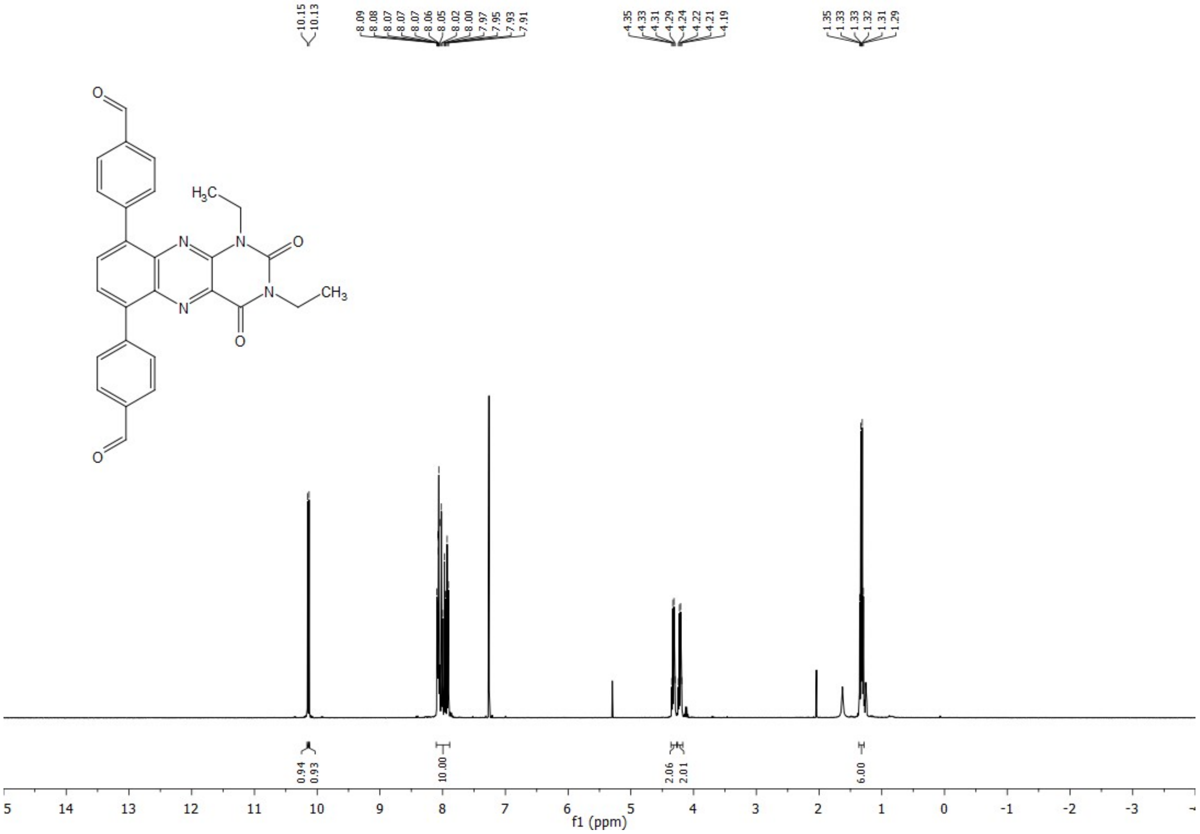

Figure S 46: ^{1}H-NMR spectrum of 1,3-diethyl-6,9-bis-(4-formylphenyl)-alloxazine FEAx ($CDCl_3$, 400 MHz).

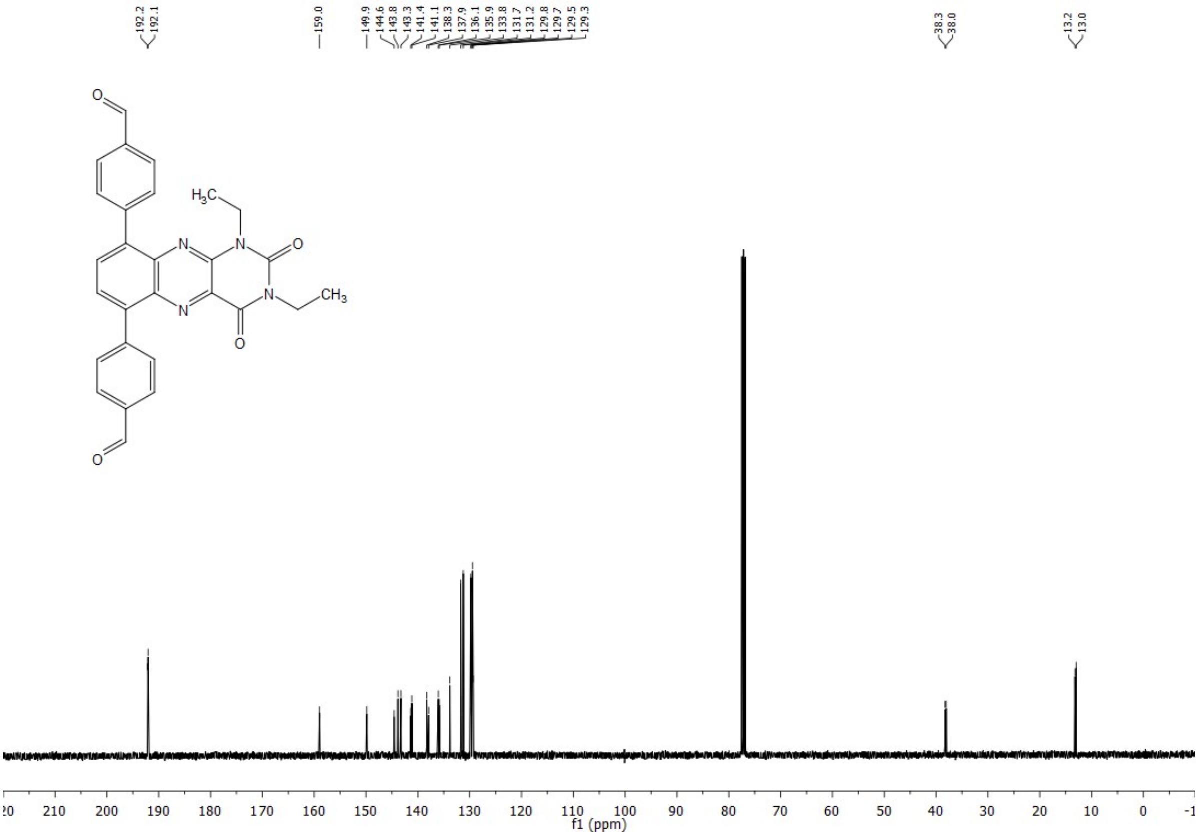

Figure S 47: ^{13}C-NMR spectrum of 1,3-diethyl-6,9-bis-(4-formylphenyl)-alloxazine FEAx ($CDCl_3$, 101 MHz).

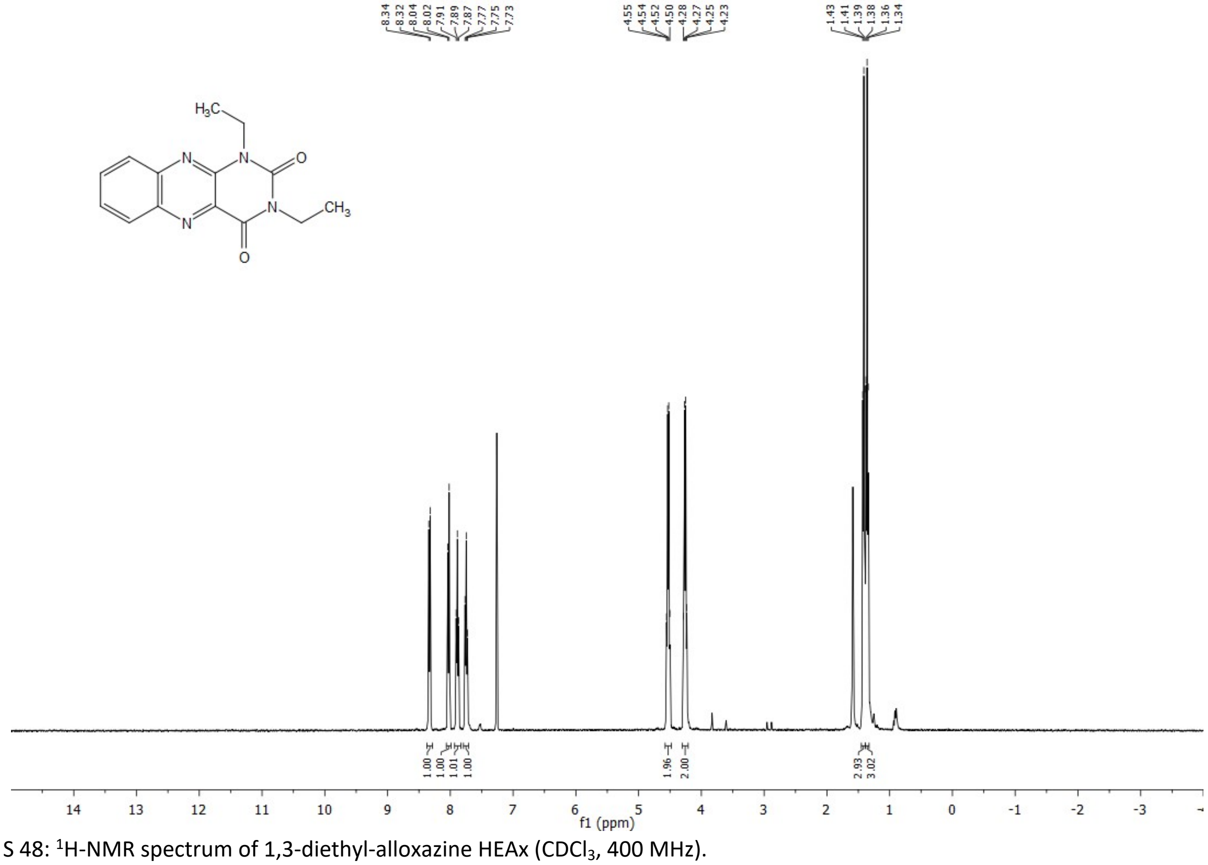

Figure S 48: ^{1}H-NMR spectrum of 1,3-diethyl-alloxazine HEAx ($CDCl_3$, 400 MHz).

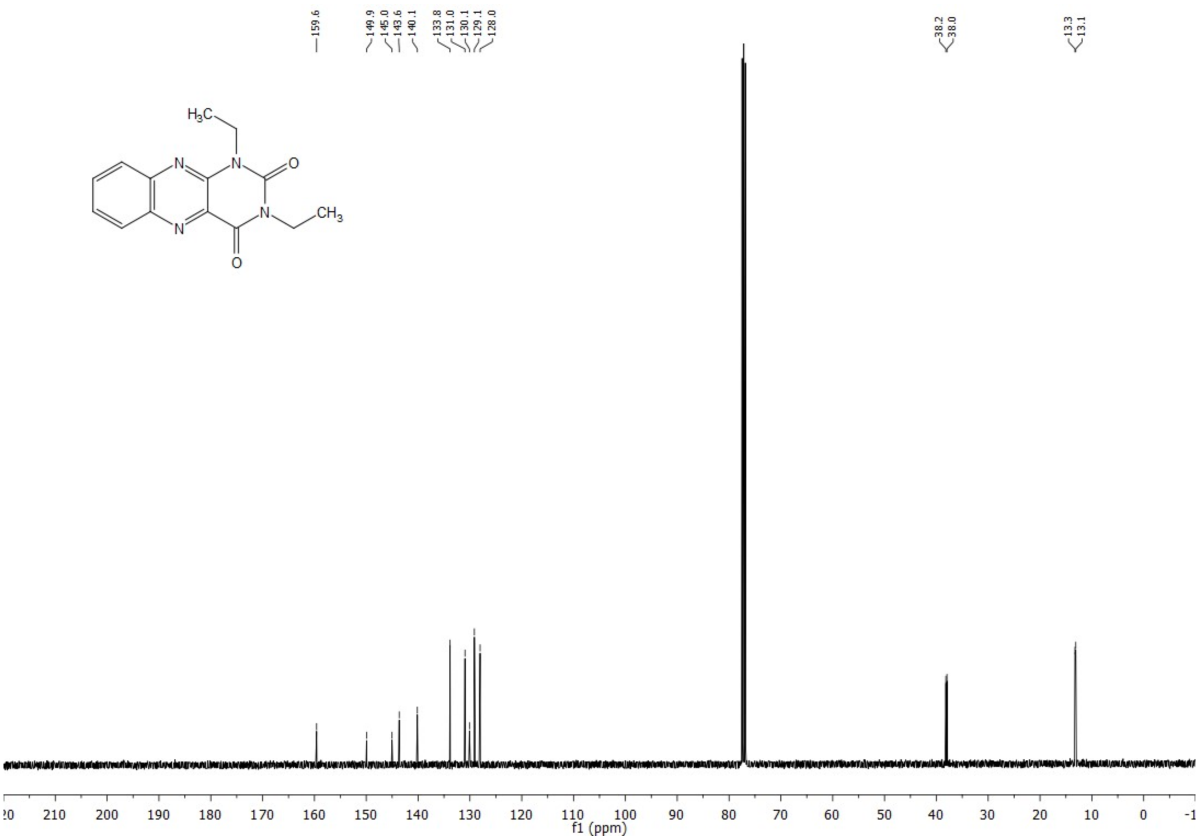

Figure S 49: ^{13}C-NMR spectrum of 1,3-diethyl-alloxazine HEAx ($CDCl_3$, 101 MHz).

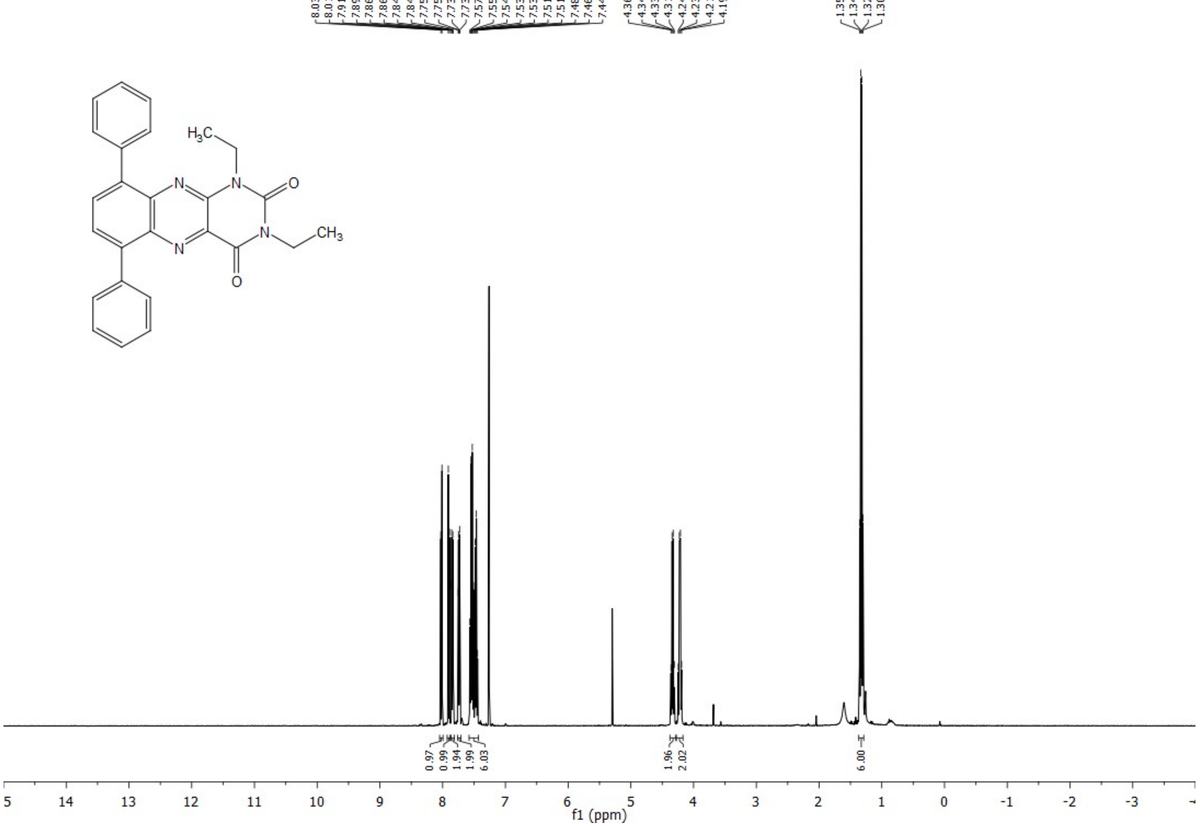

Figure S 50: ^{1}H-NMR spectrum of 1,3-diethyl-6,9-diphenyl-alloxazine PEAx ($CDCl_3$, 400 MHz).

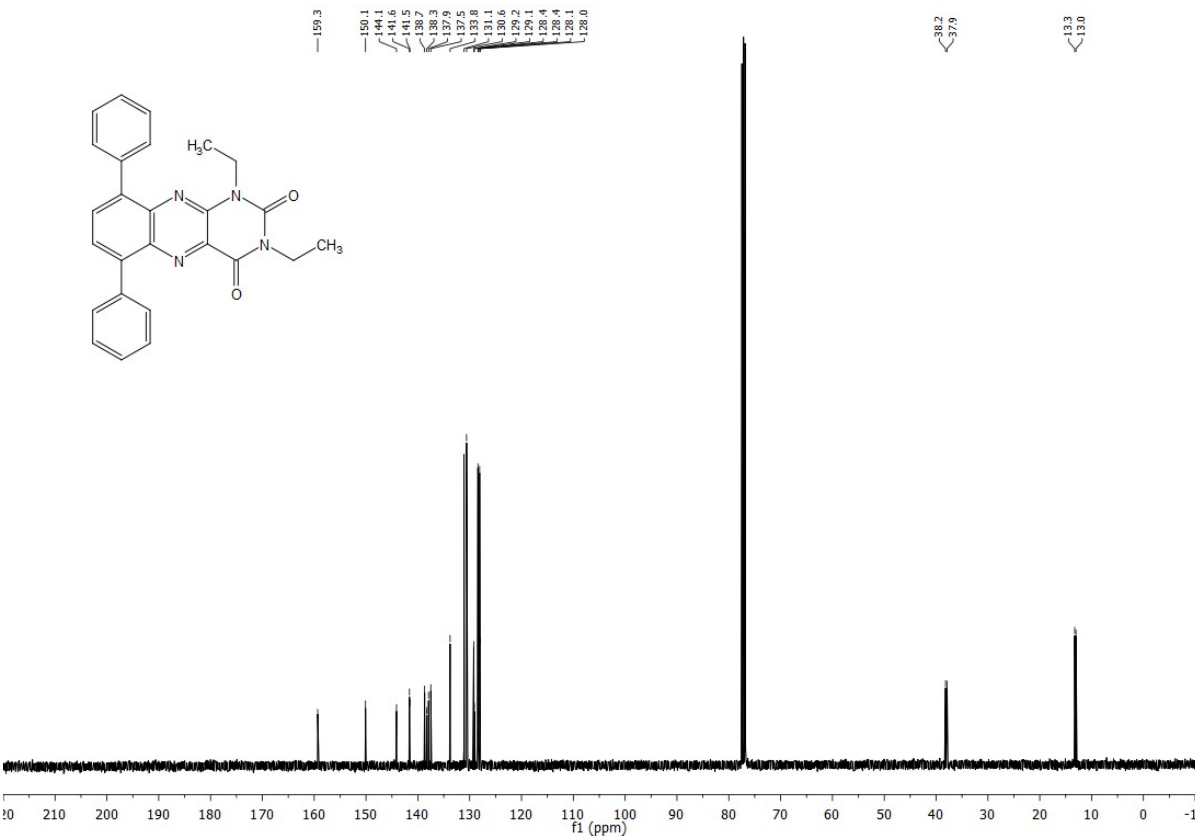

Figure S 51: ^{13}C-NMR spectrum of 1,3-diethyl-6,9-diphenyl-alloxazine PEAx ($CDCl_3$, 101 MHz).

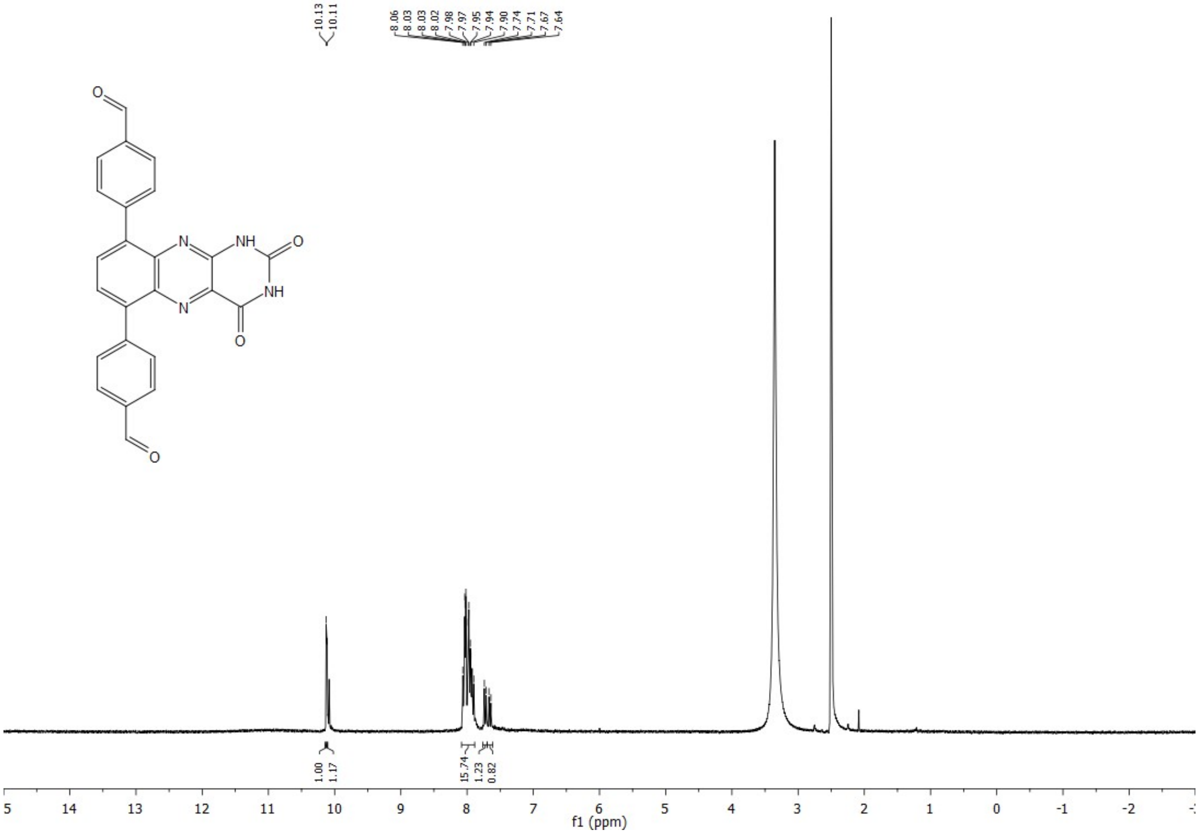

Figure S 52: ^{1}H-NMR spectrum of 6,9-bis-(4-formylphenyl)-alloxazine (FAx) (DMSO-d_6, 270 MHz).

References

1 G. M. Sheldrick, *Acta Crystallogr., Sect. A*, 2015, **71**, 3–8.
2 G. M. Sheldrick, *Acta Crystallogr., Sect. C*, 2015, **71**, 3–8.
3 O. V. Dolomanov, L. J. Bourhis, J. A. K. Howard and H. Puschmann, *J. Appl. Crystallogr.*, 2009, **42**, 339.
4 A. Macchioni, G. Ciancaleoni, C. Zuccaccia and D. Zuccaccia, *Chem. Soc. Rev.*, 2008, **37**, 479–489.
5 J. E. Tanner, *J. Chem. Phys.*, 1970, **52**, 2523–2526.
6 C. Adamo and V. Barone, *J. Chem. Phys.*, 1999, **110**, 6158–6170.
7 M. Ernzerhof and G. E. Scuseria, *J. Chem. Phys.*, 1999, **110**, 5029–5036.
8 S. Grimme, J. Antony, S. Ehrlich and H. Krieg, *J. Chem. Phys.*, 2010, **132**, 154104.
9 A. Schäfer, C. Huber and R. Ahlrichs, *J. Chem. Phys.*, 1994, **100**, 5829–5835.
10 R. Ahlrichs, M. Baer, M. Haeser, H. Horn and C. Koelmel, *Chem. Phys. Lett.*, 1989, **162**, 165–169.
11 P. J. Wilson, T. J. Bradley and D. J. Tozer, *J. Chem. Phys.*, 2001, **115**, 9233–9242.
12 J. Kussmann and C. Ochsenfeld, *J. Chem. Theory Comput.*, 2015, **11**, 918–922.
13 J. Kussmann and C. Ochsenfeld, *J. Chem. Phys.*, 2013, **138**, 134114.
14 N. G. Connelly and W. E. Geiger, *Chem. Rev.*, 1996, **96**, 877–910.
15 B. P. Biswal, H. A. Vignolo-González, T. Banerjee, L. Grunenberg, G. Savasci, K. Gottschling, J. Nuss, C. Ochsenfeld and B. V. Lotsch, *J. Am. Chem. Soc.*, 2019, **141**, 11082–11092.
16 C. M. Cardona, W. Li, A. E. Kaifer, D. Stockdale and G. C. Bazan, *Adv. Mater.*, 2011, **23**, 2367–2371.
17 R. Gomes, P. Bhanja and A. Bhaumik, *Chem. Commun.*, 2015, **51**, 10050–10053.
18 L. Liu, D.-J. Hong and M. Lee, *Langmuir*, 2009, **25**, 5061–5067.
19 J. Richtar, P. Heinrichova, D. H. Apaydin, V. Schmiedova, C. Yumusak, A. Kovalenko, M. Weiter, N. S. Sariciftci and J. Krajcovic, *Molecules*, 2018, **23**, 2271.
20 R. L. Greenaway, V. Santolini, M. J. Bennison, B. M. Alston, C. J. Pugh, M. A. Little, M. Miklitz, E. G. B. Eden-Rump, R. Clowes, A. Shakil, H. J. Cuthbertson, H. Armstrong, M. E. Briggs, K. E. Jelfs and A. I. Cooper, *Nat. Commun.*, 2018, **9**, 2849.
21 B. Atkinson and Di M., *Trans. Faraday Soc.*, 1958, **54**, 1331–1339.
22 C. Ayed, W. Huang and K. A. I. Zhang, *Front. Chem. Sci. Eng.*, 2020, **11**, 31.
23 P. K. Walia, M. Sharma, M. Kumar and V. Bhalla, *RSC Adv.*, 2019, **9**, 36198–36203.
24 S. Fukuzumi and S. Kuroda, *Res. Chem. Intermed.*, 1999, **25**, 789–811.
25 S. Higashimoto, N. Suetsugu, M. Azuma, H. Ohue and Y. Sakata, *J. Catal.*, 2010, **274**, 76–83.
26 C. Zeng, N. Zhang, C. M. Lam and R. D. Little, *Org. Lett.*, 2012, **14**, 1314–1317.
27 H. G. Roth, N. A. Romero and D. A. Nicewicz, *Synlett*, 2016, **27**, 714–723.
28 J.-H. Hong, Y.-K. Hwang, J.-Y. Hong, H.-J. Kim, S.-J. Kim, Y. S. Won and S. Huh, *Chem. Commun.*, 2011, **47**, 6963–6965.
29 A. Attour, S. Rode, T. Bystron, M. Matlosz and F. Lapicque, *J. Appl. Electrochem.*, 2007, **37**, 861–870.
30 X. Lu, X.-X. Li, Y.-M. Lee, Y. Jang, M. S. Seo, S. Hong, K.-B. Cho, S. Fukuzumi and W. Nam, *J. Am. Chem. Soc.*, 2020, **142**, 3891–3904.
31 J. Dadová, S. Kümmel, C. Feldmeier, J. Cibulková, R. Pažout, J. Maixner, R. M. Gschwind, B. König and R. Cibulka, *Chem. Eur. J.*, 2013, **19**, 1066–1075.
32 A. Klamt and G. Schüürmann, *J. Chem. Soc., Perkin Trans. 2*, 1993, 799–805.
